# Supplementary material for: Short-term impacts of Universal Basic Income on population mental health inequalities in the UK: A microsimulation modelling study
Source: PLoS Med. 2024 Mar 4;21(3):e1004358. doi: 10.1371/journal.pmed.1004358 (PMC10947674; doi:10.1371/journal.pmed.1004358)
Supplement: S1 Appendix — Table A: Key model assumptions of UKMOD and SimPaths. Table B: Effect estimates for use in Step 2 of SimPaths causal mental health module. Table C: All individual benefits retained and/or suspended in each UBI scenario. Table D: Alternative effect estimates for use in Step 2 of SimPaths causal mental health module during sensitivity analyses. Figure A: Internal validation graphs from the SimPaths GUI contrasting predicted outcomes with observed Understanding Society data from 2011–2017 (yo = years old). Figure B: Cumulative mean prevalence of common mental disorder and poverty by number of model iteratio. Figure C: Prevalence of common mental disorder (CMD) in SimPaths versus the Health Survey for England from 2012–2018. Table E: Population-level economic impacts of Universal Basic Income (UBI) policies modelled in UKMOD. Figure D: Gainers and losers by household income decile (before housing costs) ranging from low to high, with Partial UBI compared with baseline tax/benefit policies in 2023 (Scenario 2). Figure E: Gainers and losers by household income decile (before housing costs) ranging from low to high, with Full UBI compared with baseline tax/benefit policies in 2023 (Scenario 3). Table F: Median income, prevalence of poverty, employment rate, and mean hours worked in baseline scenario and three simulated Universal Basic Income (UBI) scenarios from 2022–2026 (95% uncertainty intervals). Table G: Estimated prevalence of common mental disorders (CMD) and mental health inequalities in baseline scenario and three simulated Universal Basic Income (UBI) scenarios from 2022–2026 (95% uncertainty intervals). Figure G: Estimated relative (left panel) and slope (right panel) indices of inequality by education for common mental disorder (CMD) in modelled Universal Basic Income (UBI) policies from 2022–2026. Table H: Estimated prevalence of common mental disorders (%) in baseline scenario and three simulated Universal Basic Income (UBI) scenarios from 2022–2026 stratifie [file pmed.1004358.s001.pdf]

Short-term impacts of Universal Basic Income on population mental health inequalities in the UK: A microsimulation modelling study - Supplementary Appendix

Rachel M Thomson, Daniel Kopasker, Patryk Bronka, Matteo Richiardi, Vladimir Khodygo, Andrew J Baxter, Erik Igelström, Anna Pearce, Alastair H Leyland, S Vittal Katikireddi

Table of Contents

Technical details of UKMOD and SimPaths 2
Additional detail on Universal Basic Income (UBI) interventions modelled 6
Additional detail on sensitivity analyses 10
Model validation 12
Fiscal and distributive impacts of UBI policies modelled 16
Additional results from primary analysis 19
Results from structural sensitivity analyses 25
Results from analytical sensitivity analysis 40
Results for secondary outcome (GHQ Likert score) 43
References 48

Technical details of UKMOD and SimPaths

The microsimulation modelling presented in this paper is made up of two discrete components:

1. A static tax-benefit microsimulation model (UKMOD [1], University of Essex)
2. A dynamic, stochastic, discrete-time microsimulation model (SimPaths [2], University of Essex)

The Health Equity and its Economic Determinants (HEED) project, funded by the European Research Council, aims to use dynamic microsimulation to assess potential impacts of European taxation and social security policies on population health and health inequalities in the short, medium and long term [3]. While the final models iterated through the HEED work will include multiple European countries (each with a synthetic population) and multiple health outcomes including mortality, at present this proof-of-principle version focuses on mental health outcomes in the UK. Development work is ongoing, and a broad range of collaborators have contributed to the current working version of the model.

The key assumptions of UKMOD and SimPaths in relation to this work are illustrated in Table A.

<u>Table A: Key model assumptions of UKMOD and SimPaths</u>

	Structural	Theoretical
**UKMOD**	No macroeconomic/second order impacts of the policy intervention(s)	Outstanding costs of intervention(s) can be met by policy levers outside income tax and national insurance
	No microeconomic (behavioural) impacts included within UKMOD	
**SimPaths**	Conditional independence assumption: all processes are modelled as independent, though they are based on lagged variables determined by other processes	Primary effects/causal pathways of importance from intervention to outcome occur via income/poverty and employment status
	Partial equilibrium model of labour supply i.e., models labour supply (worker side of the market) but not labour demand (firm side of the market). Wage structure is considered constant.	Mental health effects of poverty and employment transitions resulting from the intervention are equivalent to those experienced organically (in observational data)
	No interactions between individuals within the model, other than the impacts of household/benefit-level decisions on individuals within that unit	Fundamental rules of individuals' decision-making regarding utility, value of leisure time, and labour supply do not differ between intervention(s) and baseline
	Processes are ordered as in Figure 1	

<u>Overview of UKMOD</u>

UKMOD is used to model the effects of tax-benefit policies (in our case, Universal Basic Income) on household incomes for a representative sample of the UK population, drawn from the Family Resources Survey. It originates from the UK component of EUROMOD (a long-standing EU-wide tax-benefit microsimulation model currently maintained by the European Commission [4]) and was first released in 2019. The model includes a representation of the UK tax-benefit system, including income tax and social security policies applied at the UK level and within the devolved administrations (Northern Ireland, Scotland, and Wales) [5]. Researchers using the model therefore have the ability to modify existing policies (in terms of both eligibility and generosity), suspend existing policies (or individual elements of these), and to create novel policies. Model components

are available for download via the University of Essex's Centre for Microsimulation and Policy Analysis (CeMPA), who run regular courses on its use [6].

As a static model, UKMOD's population and their behaviours are fixed when analysing the effect of policies, meaning that the output for each modelled policy represents its deterministic effect on the input population (also known as morning-after effects). UKMOD has undergone extensive validation, and is revalidated prior to each new public release. The version used for this analysis was UKMOD B1.03, updated in December 2022 to reflect all of the UK and devolved Governments' intended fiscal policies announced prior to that date.

Overview of SimPaths

SimPaths is a microsimulation model of individual and household life course events which was initially created to study the effects of economic insecurity over time; in a previous iteration it was known as LABSim [7]. It was developed by CeMPA using JAS-mine, an open-source, object-oriented Java-based platform specifically designed for discrete-event simulations including microsimulation [8]. It is written to allow easy integration with outputs from UKMOD and/or EUROMOD, and can currently be run for both the UK and Italy. An open-source version of the model is available on GitHub: https://github.com/centreformicrosimulation/SimPaths.

As noted in the main paper, the econometric component of the model includes six modules simulating the life course of a representative set of individuals across a single year: Demography, Education, Health, Household composition, Non-labour market income, and Labour supply. Modifications have been made to facilitate simulation of the COVID-19 period, though these were not utilised in our analyses as our modelling does not include the affected years.

The complete technical details of SimPaths are provided in a CeMPA working paper [2]. We therefore do not replicate this description in the Appendix, but instead provide an overview of the additional factors most pertinent to understanding the modelling results we present in our paper: an in-depth description of the causal mental health module used for these analyses (in this section), and an outline of the modifications made to facilitate the sensitivity analyses (page 10).

SimPaths' causal mental health module

The HEED project added a new causal mental health module to SimPaths, using causal epidemiology methods to estimate the effects of economic transitions on mental health. The outcome used is the General Health Questionnaire (GHQ-12), a commonly-used epidemiological survey tool which measures symptoms of psychological distress [9]. It is included in the model as both a continuous measure (GHQ-36, sometimes referred to as GHQ Likert) and as a more easily interpretable binary measure of whether an individual is likely to experience a common mental disorder (CMD). Since this second measure is the primary outcome in our study, it will be the focus of this Appendix.

The module is separated into two discrete parts:

- A Step 1 prediction process, which estimates an individual's provisional likelihood of experiencing CMD based on sociodemographic characteristics.
- A Step 2 re-estimation process, where the previous predicted likelihood is modified based on the economic transitions an individual has experienced in that simulated year.

Initial prediction: Individuals aged 16 years and older are assigned a probability of experiencing CMD determined by a logit model conditional on age, age squared, gender, education, housing tenure, marital status, lagged number of children, lagged physical health, region, lagged income quintile, and

lagged CMD status. These initial prediction variables were selected based on existing literature and a directed acyclic graph (DAG) of the likely causal framework, which has been published previously [10]. The regression coefficients used to parameterise the Step 1 predictions were calculated using data from waves 1-10 of Understanding Society. For the GHQ Likert score, the same process is followed using linear regression.

Causal effect estimation: Individuals aged 25-64 years have their Step 1 likelihood of CMD modified according to which economic transitions they have experienced between the preceding year of the simulation and the current year. Possible exposures are:

- Employed to Unemployed* (reference Employed to Employed)
- Unemployed to Employed (reference Employed to Unemployed)
- Persistently Unemployed (unemployed in both years; reference Employed to Unemployed)
- Non-Poverty to Poverty (reference Non-Poverty to Non-Poverty)
- Poverty to Non-Poverty (reference Non-Poverty to Poverty)
- Persistent Poverty (in poverty in both years; reference Non-Poverty to Poverty)
- Real income change (applied to the continuous income change experienced between years)
- Real income decrease (dummy variable indicating a real income change was a decrement)

** 'Unemployed' within SimPaths Step 2 includes all those not employed and not a student, retired, or long-term sick or disabled*

As in Step 1, a logit model determines a probability of experiencing CMD based on the cumulative impact of all economic transitions experienced by an individual. This second probability is then added to the probability from Step 1 to modify an individual's overall likelihood accordingly, giving a finalised likelihood of CMD which takes into account both preliminary demographics and their recent experience of economic transitions. For the GHQ Likert score, the same process is followed using linear regression.

In the version of SimPaths used for this analysis, effect estimates for transitions between economic states were calculated using double-robust marginal structural modelling, a causally-informed statistical approach using inverse probability of treatment weights based on DAGs. We have published the full specification of these models in detail elsewhere [10, 11]. Based on peer review feedback, we re-modelled the causal effects of employment transitions using the International Labour Organization definitions for employed/unemployed for this analysis [12], in contrast with the published paper where exposure was any form of paid work [11]. This also better aligns with systematic review estimates used in our sensitivity analysis [13]. As the mental health effects of economic transitions appear to differ by gender, separate models were estimated for men and women.

Reference categories were selected to most accurately quantify the impact of the new economic transition in comparison to the last transition experienced by the individual in question (i.e., taken together, the estimates allow calculation of how damaging an initial transition into poverty is for mental health, whether one more year of poverty is as damaging as the first year, and to what extent mental health will recover if/when someone exits poverty).

Effect estimates measuring the small additional impact of real income change and real income decreases were calculated using g-computation, a similar causally-informed statistical method better suited to use with continuous exposure measures, using the same DAG.

Table B below shows the value of all effect estimates used in the Step 2 process.

Table B: Effect estimates for use in Step 2 of SimPaths causal mental health module

Exposure	β coefficient (log odds)	Variance
**MEN**		
Employed to Unemployed	1.103	0.010204
Unemployed to Employed	-1.289	0.030209
Persistent Unemployed	-0.478	0.014519
Non-Poverty to Poverty	0.123	0.004278
Poverty to Non-Poverty	-0.137	0.005879
Persistent Poverty	-0.030	0.004628
Real Income Change	-0.004	1.12E-05
Real Income Decrease	0.004	1.16E-05
**WOMEN**		
Employed to Unemployed	0.693	0.007378
Unemployed to Employed	-1.217	0.019243
Persistent Unemployed	-0.401	0.012337
Non-Poverty to Poverty	0.240	0.003431
Poverty to Non-Poverty	-0.173	0.003866
Persistent Poverty	-0.081	0.003505
Real Income Change	-0.012	1.44E-05
Real Income Decrease	0.005	1.21E-05

Outcome is likelihood of common mental disorder. All effect estimates were calculated using Understanding Society data. Effect sizes for poverty and employment state transitions were calculated using marginal structural modelling as outlined in Thomson et al. 2022. Real income change effect estimates were calculated using g-computation.

Additional detail on Universal Basic Income (UBI) interventions modelled

A workshop was held with the project's Advisory Group to explore their preferences in relation to the UBI interventions that would be modelled in May 2022. The four questions considered by the group were:

- What **level** should the UBI be set at?
- What counts as '**universal**'?
- Should any **existing means-tested benefits** be retained in addition to the UBI?
- How should the UBI be **funded**?

On **levels**, there was consensus on ideally testing at least two different options e.g., a full versus a partial UBI. To achieve policy relevance, there was strong support for trying to tie in the UBI levels tested to what was happening in the real world. Suggestions included selecting a UBI based on the Minimum Income Standard from JRF, testing a version of the Welsh UBI pilot for caregivers, or using established thresholds such as the poverty level or levels associated with absolute destitution. The group were in favour of utilising existing research/development work where possible, though supported the idea of creating a model from scratch if this was most suitable to meet the rest of their criteria.

On **universality**, there was agreement that all population groups including children, adults, and the elderly should receive some form of UBI in all schemes modelled, to ensure they were truly universal and met the definition of a UBI.

On **existing benefits**, there was considered discussion around the fact that retention of any means-tested benefits would compromise some of the simplicity arguments in support of UBI. However, this had to be balanced against a wish to ensure no population group systematically 'lost out' from a UBI. Therefore, the group felt that it would be helpful to model a UBI scheme that ensured all groups likely to have residual need beyond the UBI value had their incomes maintained above this level.

On **funding**, there was discussion about the need to be pragmatic and come up with a single 'sensible' scenario for funding that allowed a reasonable interpretation of effects on mental health inequalities, rather than being drawn into the considerable debate on the 'best' way to fund UBI. The preferred option was to model some politically feasible income tax policy changes, but allow for a deficit which would need to be covered from other sources (suggestions in existing literature include the creation of novel wealth, automation, or carbon taxes; clearly the feasibility of this, while outside the scope of our work, would need to be explored in detail prior to implementation).

From the above preferences, the three UBI scenarios modelled in the final analysis were designed and implemented within UKMOD, based on the recommendations of the Citizens' Basic Income Feasibility Study Steering Group [14]:

1. A Partial UBI set at the level of existing unemployment benefits with retention of means-tested benefits for disability, housing, childcare, and limited capability for work.
2. A Full UBI meeting the Minimum Income Standard for living in the UK.
3. A Full+ UBI meeting the Minimum Income Standard with retention of means-tested benefits for disability, housing, childcare, and limited capability for work.

As the recommendations were published in 2020, the suggested UBI amounts were manually uprated prior to implementation in UKMOD. For the Partial UBI, this was achieved using the Bank of England inflation calculator (https://www.bankofengland.co.uk/monetary-policy/inflation/inflation-

calculator), uprating initial values to those for January 2023. For the Full and Full+ UBI, as the recommendations had been based on the 2018 Minimum Income Standard, these were uprated to meet the most recently published 2022 Minimum Income Standard [15]. Full details of the individual benefits retained and suspended in each UBI scenario are shown in Table C.

Table C: All individual benefits retained and/or suspended in each UBI scenario

Policy Scenario	Benefits Retained	Benefits Suspended
**Partial UBI**	Attendance allowance<br>DLA & PIP (+ mobility allowance)<br>Incapacity benefit<br>Contributory ESA<br>Income related ESA<br>Industrial injuries pension<br>Occupational pension<br>Severe disablement allowance<br>Statutory sick pay<br>War pension<br>Maternity pay/allowance<br>Paternity pay<br>Housing benefit<br>Council tax benefit<br>Sure Start maternity grant<br>Best Start grant (Scotland)<br>Student payments<br>Student loan<br>Scottish child winter heating assistance<br>Winter fuel allowance<br>Cost of living payment<br>Best Start Foods (Scotland)<br>Free school meals (Scotland)<br>School clothing grant (Scotland)<br>Discretionary housing payments (Scotland)<br><br>**Universal Credit components:**<br>Additional amount for disabled child<br>Limited capacity for work and work-related activity<br>Carer element<br>Childcare costs element	Invalid carer's allowance<br>Scottish Carer's allowance supplement<br>State pension<br>Training allowance<br>Widow's pension<br>Working Tax Credit<br>Child tax credit<br>Income support<br>Pension credit<br>Income-based JSA<br>Child benefit<br>Scottish Child Payment<br>Scottish Child Payment bridging payments<br>Unemployment benefit (JSA)<br><br>**Universal Credit components**:<br><br>Standard allowances for adults<br>Family element<br>Child element<br><br>Benefit cap
**Full UBI**	Occupational pension<br>Statutory sick pay<br>Statutory maternity pay/allowance<br>Student payments<br>Student loan	Attendance allowance<br>Disability living allowance + mobility allowance<br>PIP living allowance<br>PIP mobility<br>Incapacity benefit<br>Contributory ESA<br>Industrial injuries pension<br>Invalid carer's allowance<br>Scottish Carer's allowance supplement<br>State pension

<table>
<tr><td></td><td></td><td>Severe disablement allowance
Training allowance
War pension
Widow’s pension
Working Tax Credit
Child tax credit
Income support
Income related ESA
Pension credit
Housing benefit
Council tax benefit
Income-based JSA
Universal Credit
Sure Start maternity grant
Best Start grant (Scotland)
Scottish Child Payment
Scottish Child Payment bridging payments
Scottish Child winter heating assistance
Winter fuel allowance
Child benefit
Unemployment benefit (JSA)
Cost of living payment
Best Start Foods (Scotland)
Free school meals (Scotland)
School clothing grant (Scotland)
Discretionary housing payments (Scotland)

Universal Credit components:

Standard allowances for adults
Family element
Child element
Additional amount for disabled child
Limited capacity for work and work-related activity
Carer element
Childcare costs element

Benefit cap</td></tr>
<tr><td>Full+ UBI</td><td>Attendance allowance
DLA & PIP (+ mobility allowance)
Incapacity benefit
Contributory ESA
Income related ESA
Industrial injuries pension
Occupational pension
Severe disablement allowance
Statutory sick pay
War pension
Maternity pay/allowance
Paternity pay</td><td>Invalid carer’s allowance
Scottish Carer’s allowance supplement
State pension
Training allowance
Widow’s pension
Working Tax Credit
Child tax credit
Income support
Pension credit
Income-based JSA
Child benefit
Scottish Child Payment</td></tr>
</table>

	Housing benefit<br>Council tax benefit<br>Sure Start maternity grant<br>Best Start grant (Scotland)<br>Student payments<br>Student loan<br>Scottish child winter heating assistance<br>Winter fuel allowance<br>Cost of living payment<br>Best Start Foods (Scotland)<br>Free school meals (Scotland)<br>School clothing grant (Scotland)<br>Discretionary housing payments (Scotland)<br><br>**Universal Credit components:**<br>Additional amount for disabled child<br>Limited capacity for work and work-related activity<br>Carer element<br>Childcare costs element	Scottish Child Payment bridging payments<br>Unemployment benefit (JSA)<br><br>**Universal Credit components**:<br><br>Standard allowances for adults<br>Family element<br>Child element<br><br>Benefit cap

DLA = Disability Living Allowance. PIP = Personal Independence Payment. ESA = Employment and Support Allowance. JSA = Jobseekers Allowance.

Additional detail on sensitivity analyses

As described in the paper, three sensitivity analyses (SAs) were conducted:

1. A structural sensitivity analysis altering labour supply utility assumptions under Full UBI.
2. A structural sensitivity analysis altering unemployment/mental health effects under Full UBI.
3. An analytical sensitivity analysis using causal estimates sourced from systematic reviews.

SA 1 was intended to model a situation where individuals did not respond to receipt of an unearned Full UBI by reducing working hours (as they would be predicted to do in response to large changes in earned income/salary), based on empirical evidence that the employment effects of UBI may be small [16]. To implement this within SimPaths, the model code was altered to allow two versions of the Labour Supply module to be used for Full UBI scenarios: the original, to be used pre-intervention (in 2022), and a modified version to be used post-intervention (from 2023 onwards). In the modified version of the Labour Supply module, the utility values used to inform decisions of simulated individuals about the number of hours they should work were manually altered so that employment rates for the whole population, and for men and women separately, were comparable to the baseline/non-intervention scenarios.

To avoid introducing additional unevidenced assumptions, only the coefficient for the utility an individual assigned to their leisure time was altered. The original empirically derived utility coefficients differ between men and women e.g., -1.68 for single men versus -0.62 for single women. Therefore, there were also gender differences in the extent to which these required to be altered for our sensitivity analysis – they were reduced by 0.01/0.014 points for men and 0.0045/0.0075 points for women for Full/Full+ UBI analyses respectively. These values were arrived at iteratively based on repeated analyses and direct comparison of the employment outcomes generated for each new version of the model with the baseline outcomes. Changes were made proportionally for all individuals i.e., the utility value for single men was altered to the same extent as the value for men in couples. While this approach is relatively crude, it nonetheless achieved our primary purpose: to simulate the mental health effects of a UBI which did not result in significantly reduced employment rates or average working hours compared with baseline (see Table I for evidence of efficacy). No other changes were made to the base model as used in the primary analysis.

SA 2 was intended to model a situation where the effects of stopping work on mental health in response to a Full UBI were more akin to the effects of voluntarily exiting the labour market, rather than the effects of unemployment more generally. Additional marginal structural models were therefore run on Understanding Society data to estimate the effect of transitions in and out of economic inactivity (defined as not in employment and not seeking work), following the same estimation strategy used for the primary causal estimates. To implement these changes into SimPaths, the model code was altered to allow two versions of the Mental Health module to be used for Full UBI scenarios: the original, to be used pre-intervention (in 2022), and a modified version to be used post-intervention (from 2023 onwards). In the modified version of the Mental Health module, the regression coefficients used to generate probabilities of CMD in Step 2 of the model were altered as shown in Table D. Compared with those used in the primary analysis, the effects of 'unemployment' transitions were considerably smaller, particularly for men. No other changes were made to the base model as used in the primary analysis.

Table D: Alternative effect estimates for use in Step 2 of SimPaths causal mental health module during sensitivity analyses

Exposure	β coefficient (log odds)	Variance
**MEN – economic inactivity rather than unemployment (Full UBI years only)**		
Employed to Unemployed	0.150	0.035403
Unemployed to Employed	-0.334	0.051369
Persistent Unemployed	-0.063	0.026213
**WOMEN – economic inactivity rather than unemployment (Full UBI years only)**		
Employed to Unemployed	0.170	0.00406
Unemployed to Employed	-0.287	0.006615
Persistent Unemployed	-0.220	0.007319
**MEN & WOMEN – sourced from systematic reviews**		
Employed to Unemployed	0.345	0.007208
Unemployed to Employed	-0.635	0.004597
Persistent Unemployed	-0.145	0.003295
Non-Poverty to Poverty	0.152	0.001798
Poverty to Non-Poverty	-0.152	0.001798

UBI = Universal Basic Income. Effect sizes for economic inactivity transitions were calculated using marginal structural modelling in Understanding Society data as outlined in Thomson et al. 2022. Systematic review sources for all other estimates are outlined in the main text of the Appendix above.

SA 3 was intended to test whether the use of effect estimates from evidence synthesis of existing literature (rather than those from our own epidemiological analyses) would alter our primary findings. Systematic reviews with meta-analysed effect estimates were identified considering the effects of income and poverty on mental health [13], and the effect of unemployment on mental health [17]. For the former review it was only possible to extract a single estimate for poverty transitions, as there was no differentiation made between moves in and out of poverty. Persistent poverty was also not included as an exposure, and therefore the effect for persistent poverty from the original Step 2 process was used. For unemployment, effect estimates were extracted for all required transitions. The estimates of interest were not stratified by gender in either review, so the same estimates were applied to both men and women. All estimates were converted to log odds using the conversion methods outlined in the Cochrane Handbook for Systematic Reviews [18]. To implement these changes into SimPaths, the regression coefficients used to generate probabilities of CMD in Step 2 of the model were altered as shown in Table D for all scenarios in all years. Compared with those used in the primary analysis, the effects of poverty transitions are slightly smaller (particularly for women), and the unemployment effects are considerably smaller (particularly for men). No other changes were made to the base model as used in the primary analysis.

Model validation

The ISPOR-SMDM Good Research Practices in Modelling Task Force state that transparency and validation are key to ensuring trust and confidence in the outputs of healthcare models [19]. Validity encompasses **face validity** (evaluation of the model's assumptions, data and structures), **internal validity** (evaluation of coding etc. within the model), **external validity** (comparison of the model's outputs with real-world findings), and **predictive validity** (comparison of the model's predictions with prospective real-world findings).

For this project (and for HEED more generally), **face validity** has been pursued through the integration of topic and methods experts into the development process, both as part of the Advisory/Steering Groups and in individual correspondence. A peer-reviewed protocol has been published in BMJ Open [3]. Our causal effect estimation strategy has also been peer-reviewed and published in the International Journal of Epidemiology [10] and Psychological Medicine [10, 11]. In addition, we draw on two existing open-access microsimulation models which have undergone long periods of development and are available for use by other researchers considering similar topics [1, 2].

On **internal validity**, developments to SimPaths to facilitate the addition of the causal mental health model began in 2020 following receipt of funding from the Health Foundation [20]. This development process has been iterative, with active code review and sense-checking of inputs and outputs within and across the research team at all stages. The model itself includes extensive internal validity checks within the Graphical User Interface (GUI) using reference data from Understanding Society years 2011-2017, comparing the outcomes predicted by the model with those observed in reality during those years. This is automatically generated for a wide range of economic and health outcomes, and allowed us to demonstrate that our modifications to the model did not alter its predictive validity (Figure A). As shown, we were able to add the GHQ score to this set of checks in the GUI, ensuring that we were approximating levels and trends adequately.

Figure A: Internal validation graphs from the SimPaths GUI contrasting predicted outcomes with observed Understanding Society data from 2011-2017 (yo = years old)

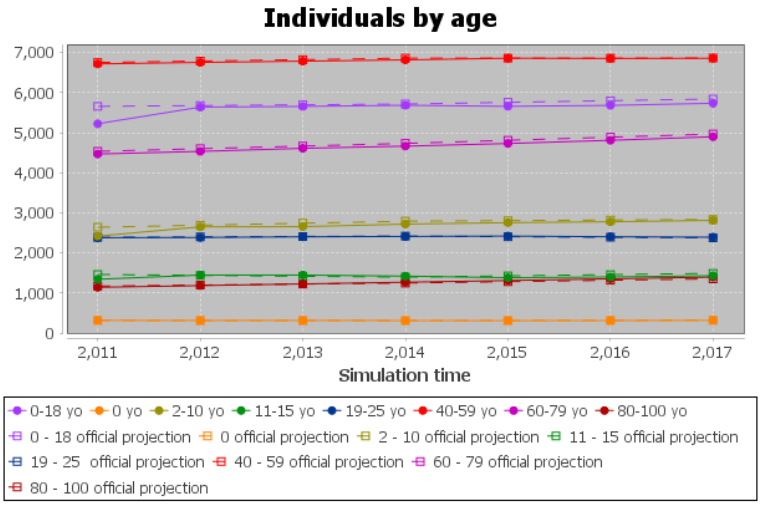

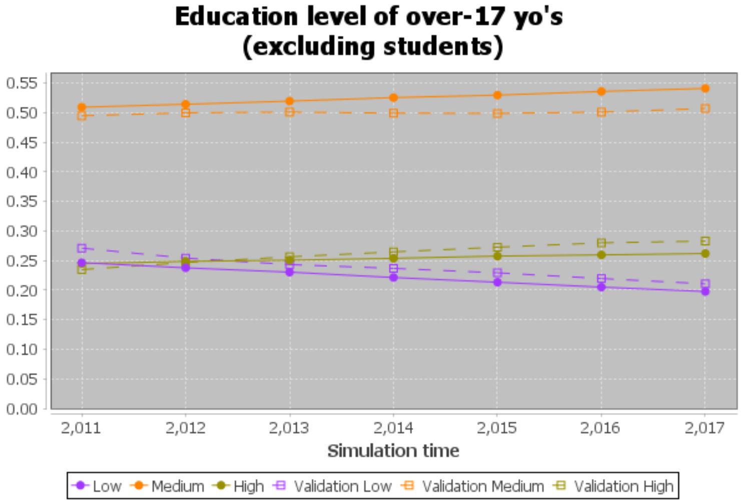

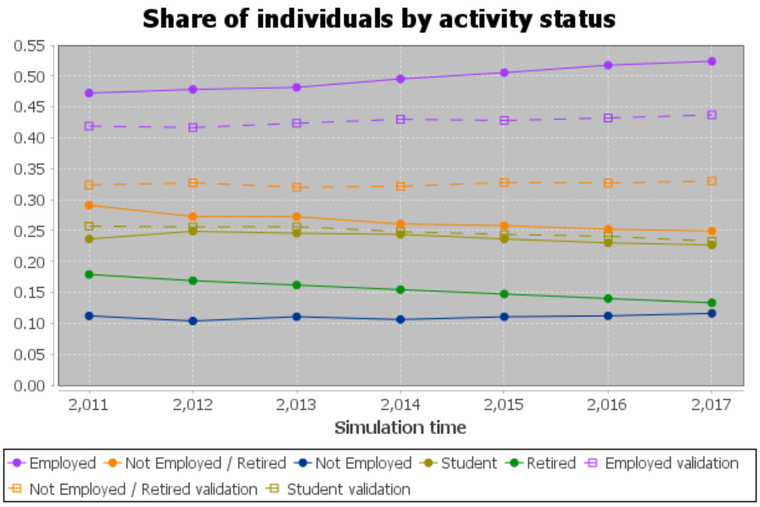
Share of individuals by activity status
Simulation time
Employed
Not Employed / Retired
Not Employed
Student
Retired
Employed validation
Not Employed / Retired validation
Student validation

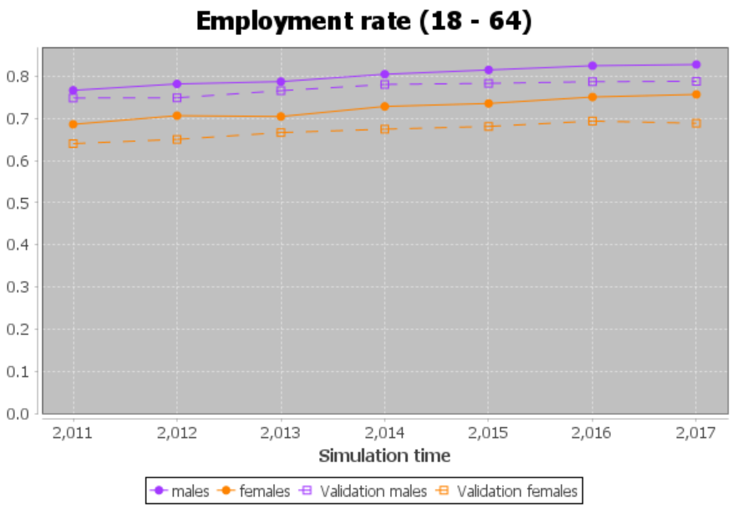
Employment rate (18 - 64)
Simulation time
males
females
Validation males
Validation females

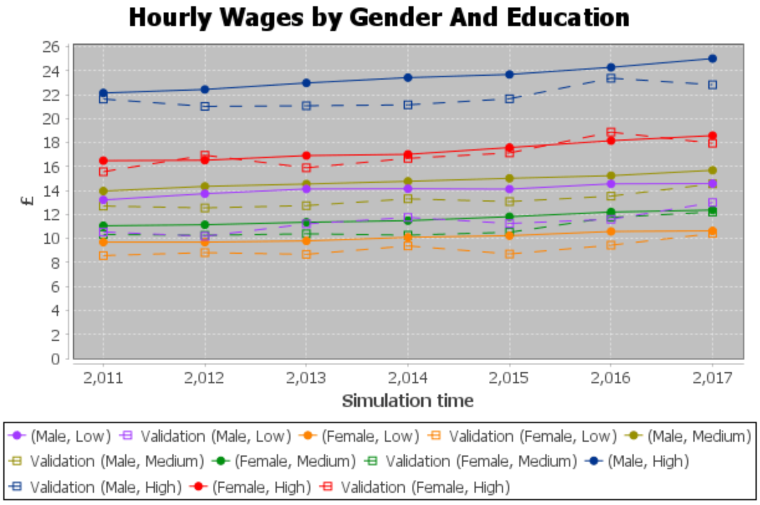
Hourly Wages by Gender And Education
£
Simulation time
(Male, Low)
Validation (Male, Low)
(Female, Low)
Validation (Female, Low)
(Male, Medium)
Validation (Male, Medium)
(Female, Medium)
Validation (Female, Medium)
(Male, High)
Validation (Male, High)
(Female, High)
Validation (Female, High)

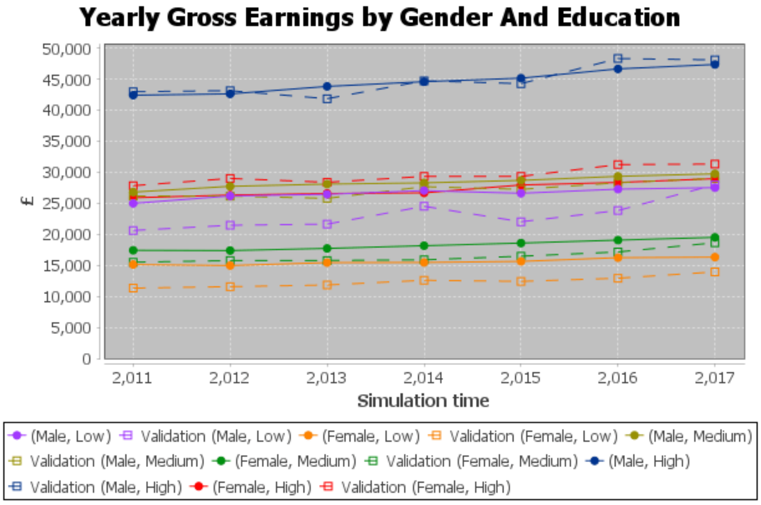
Yearly Gross Earnings by Gender And Education
£
Simulation time
(Male, Low)
Validation (Male, Low)
(Female, Low)
Validation (Female, Low)
(Male, Medium)
Validation (Male, Medium)
(Female, Medium)
Validation (Female, Medium)
(Male, High)
Validation (Male, High)
(Female, High)
Validation (Female, High)

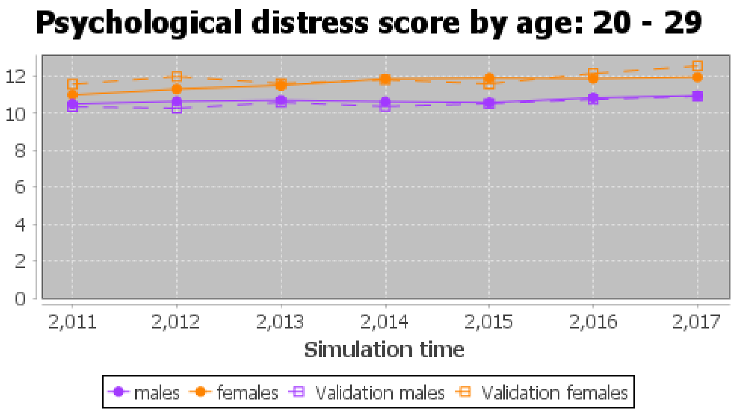
Psychological distress score by age: 20 - 29
Simulation time
males
females
Validation males
Validation females

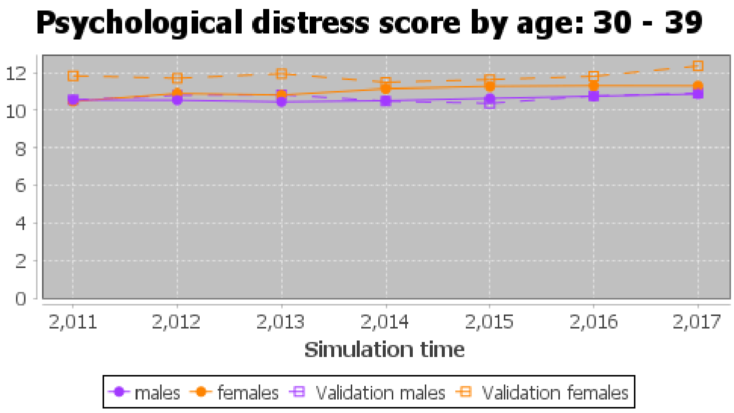
Psychological distress score by age: 30 - 39
Simulation time
males
females
Validation males
Validation females

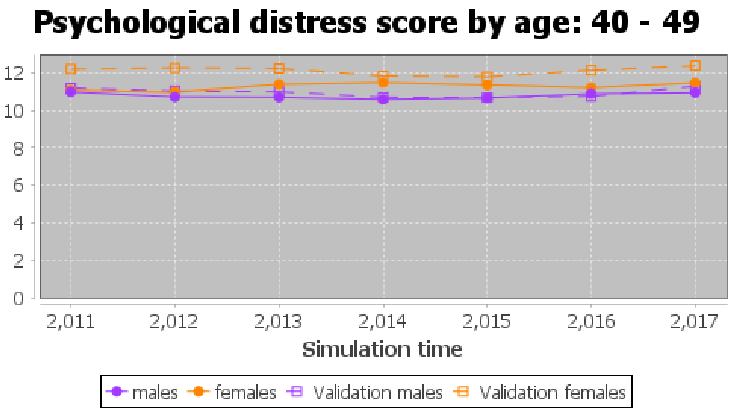
Psychological distress score by age: 40 - 49
Simulation time
males
females
Validation males
Validation females

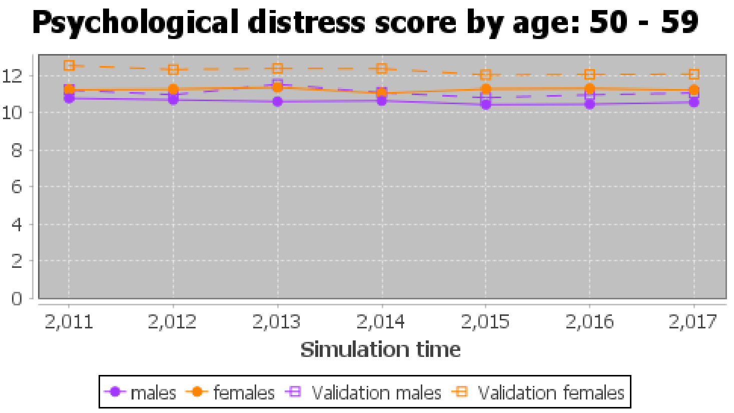
Psychological distress score by age: 50 - 59
Simulation time
males
females
Validation males
Validation females

Based on these internal validation graphs we were satisfied that the model predictions were acceptable, albeit with a small overestimation of the percentage of people in employment, a small overestimation of earnings for those with lowest education, and potentially a slight underestimation of poor mental health for older women.

As a further check on internal validity, we performed additional black box tests of the new SimPaths model to be sure that it functioned as anticipated once complete. This included the use of parameter sweeps in Step 2 of the mental health module (i.e., ensuring that entering artificially inflated or deflated poverty and/or employment causal effect estimates generated the expected direction and magnitude of effect for mental health), and the simulation of extreme artificial tax/benefit scenarios to ensure mental health and employment variables responded as anticipated (e.g., ensuring a UBI payment of £100,000/month resulted in all individuals stopping work with the standard version of the Labour Supply module). We only moved forward with simulation of the UBI scenarios presented in the paper once satisfied that these tests were successful, and reran them if there were any subsequent major changes to the model codebase.

Finally, to be sure that we were running at least the minimum number of simulations likely to appropriately account for parameter and stochastic uncertainty, we examined the variability of the cumulative mean prevalence of two outcomes of interest (CMD and poverty) by number of simulation runs (Figure B). This confirmed that completing 1000 runs of our simulations was likely to achieve sufficient stability of outputs, as relative stability was achieved at around 250 runs.

Figure B: Cumulative mean prevalence of common mental disorder and poverty by number of model iterations

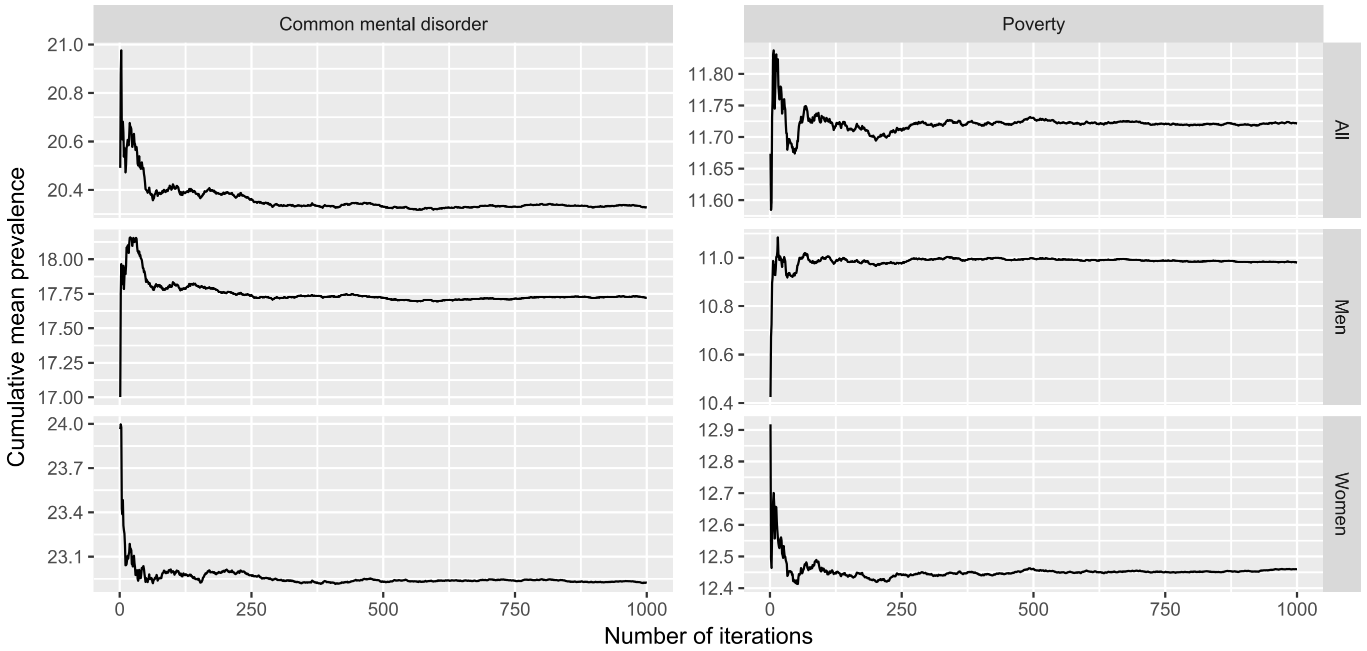

On **external validity**, we wished to compare the outputs of the model to data which were not used in the generation of either the model itself or any of its components, to ensure that both the level and trend of CMD we were predicting seemed broadly appropriate. We selected the Health Survey for England, a large representative cross-sectional survey which includes the GHQ-12 in every second year of data collection [21]. For years without data collection, we calculated an average between the preceding and following year. To ensure comparability, we excluded residents of

Northern Ireland, Scotland, and Wales from our simulated population in the comparison dataset from SimPaths. Results are shown in Figure C.

Figure C: Prevalence of common mental disorder (CMD) in SimPaths versus the Health Survey for England from 2012-2018

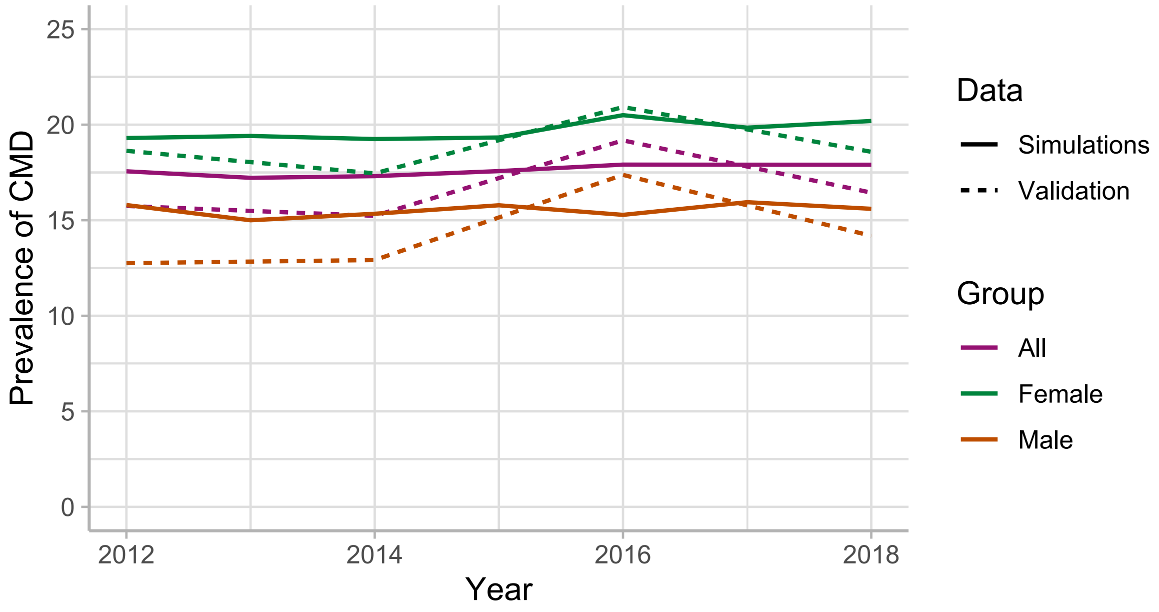

SimPaths' predictions of CMD prevalence were relatively close to the observed data from the Health Survey for England, particularly for women. The mean difference in prevalence between SimPaths and the Health Survey for the whole population across the time period was 0.89% (minimum -1.27%, maximum 2.08%); for women mean difference was 0.75% (min. -0.42%, max. 1.79%), and for men mean difference was higher at 1.11% (min. -2.09%, max. 3.04%). The trends within SimPaths were generally flatter than those in the Health Survey for England, though sample sizes for these surveys were smaller than those in our simulations (approx. 4500 in total per year).

Finally, for our modelled scenarios assessment of **predictive validity** is challenging, as there have to date been no trials of a true UBI. In our Discussion we draw some comparisons with existing evidence from reviews. However, to truly assess predictive validity outputs from our model would need to be compared with those from larger trials. The ongoing Welsh trial of a UBI for care-leavers may provide a useful comparison; while it focuses on a small and select group, the UBI is generous and at a level close to those modelled in our Full UBI scenarios [22].

Fiscal and distributive impacts of UBI policies modelled

The fiscal and distributive impacts of the policies as modelled in UKMOD are shown in Table E and Figures D-F. All three policies result in large fiscal deficits, with the largest being for Partial UBI (due to tax rises being more limited in this scenario). Partial UBI would increase UK Government spending on social security 1.8 times; Full UBI would increase it 3.6 times, and Full+ UBI 3.7 times.

As shown in Figure D (which is reproduced from UKMOD output), the groups which benefit most from Partial UBI in a relative sense are middle earners, with around 50% of the lowest earners actually seeing their incomes reduce in this policy scenario. In contrast, Full UBI scenarios are more redistributive, particularly when accompanied by means-tested benefits in the Full+ scenario (Figures E-F).

Table E: Population-level economic impacts of Universal Basic Income (UBI) policies modelled in UKMOD

	Baseline	Partial UBI	Full UBI	Full+ UBI
**UK Govt. income tax revenue**	£451.7bn	£543.3bn (+£91.6bn)	£1,061.2bn (+£609.6bn)	£1,062.4bn (+£610.8bn)
**UK Govt. benefit expenditure**	£251.3bn	£438.5bn (+£187.2bn)	£891.8bn (+£640.5bn)	£927.2bn (+£676.0bn)
**UBI-related deficit**	n/a	£95.6bn	£30.9bn	£65.2bn
**Gini coefficient**	0.3052	0.2664 (-0.0388)	0.1578 (-0.1473)	0.1443 (-0.1609)
**Share of income by decile (before housing costs)**				
**Decile 1**	3.43%	4.29% (+1.86%)	7.06% (+3.62%)	7.03% (+3.60%)
**Decile 2**	4.92%	5.52% (+0.94%)	7.28% (+2.36%)	7.34% (+2.42%)
**Decile 3**	6.05%	6.25% (+0.85%)	7.95% (+1.90%)	8.17% (+2.13%)
**Decile 4**	6.92%	7.40% (+0.87%)	8.59% (+1.67%)	8.81% (+1.89%)
**Decile 5**	7.88%	8.53% (+0.72%)	9.28% (+1.40%)	9.45% (+1.56%)
**Decile 6**	9.10%	9.60% (+0.67%)	10.05% (+0.95%)	10.17% (+1.06%)
**Decile 7**	10.35%	10.84% (+0.38%)	10.86% (+0.51%)	10.83% (+0.48%)
**Decile 8**	11.86%	12.18% (-0.08%)	11.65% (-0.21%)	11.49% (-0.37%)
**Decile 9**	14.66%	14.33% (-0.96%)	12.69% (-1.97%)	12.44% (-2.22%)
**Decile 10**	24.82%	21.07% (-5.26%)	14.59% (-10.24%)	14.26% (-10.56%)
**Share of income by household type (before housing costs)**				
**With Children**	34.03%	35.58% (+1.55%)	37.76% (+3.73%)	37.10% (+3.07%)
**No Earners**	21.05%	19.64% (-1.41%)	21.0% (-0.06%)	22.47% (+1.42%)
**One Earner**	24.24%	24.42% (+0.19%)	25.76% (+1.53%)	25.50% (+1.27%)
**Two+ Earners**	54.71%	55.93% (+1.22%)	53.24% (-1.47%)	52.02% (-2.69%)
**Three+ Children**	4.83%	5.41% (+0.58%)	6.65% (+1.83%)	6.55% (+1.72%)
**Lone Parent**	4.93%	4.67% (-0.26%)	6.06% (+1.13%)	6.05% (+1.12%)
**Disabled**	9.63%	9.20% (-0.43%)	9.37% (-0.27%)	11.16% (+1.53%)

Baseline = planned tax/benefit policies for United Kingdom. Partial UBI = UBI set at level of existing benefits. Full UBI = UBI set at level of Minimum Income Standard. Full+ UBI = Minimum Income Standard plus means-tested benefits for caring, childcare, disability, housing, limited capability for work.

Figure D: Gainers and losers by household income decile (before housing costs) ranging from low to high, with Partial UBI compared with baseline tax/benefit policies in 2023 (Scenario 2)

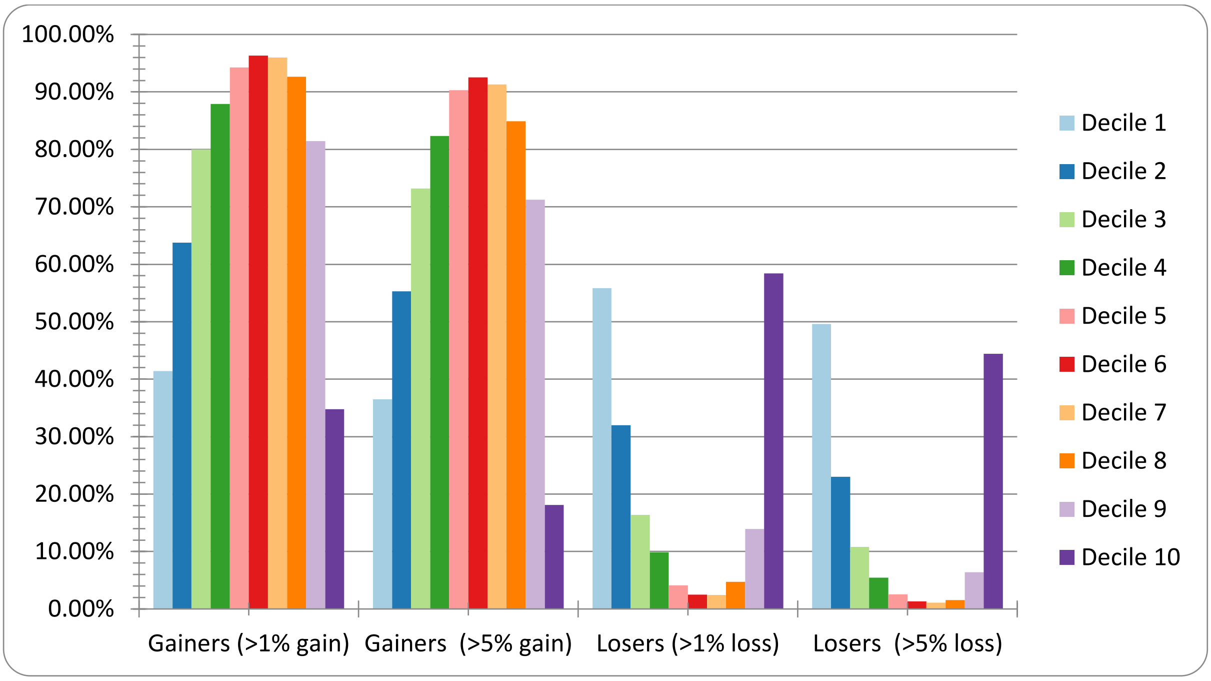

Figure E: Gainers and losers by household income decile (before housing costs) ranging from low to high, with Full UBI compared with baseline tax/benefit policies in 2023 (Scenario 3)

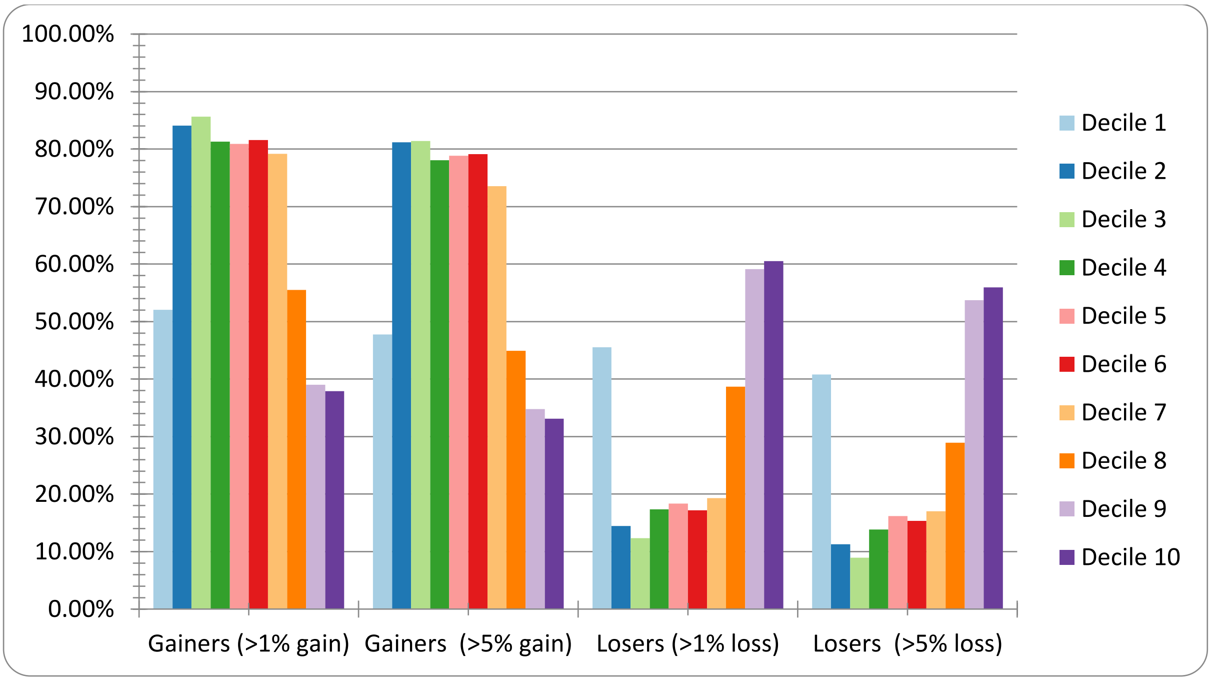

Figure F: Gainers and losers by household income decile (before housing costs) ranging from low to high, with Full+ UBI compared with baseline tax/benefit policies in 2023 (Scenario 4)

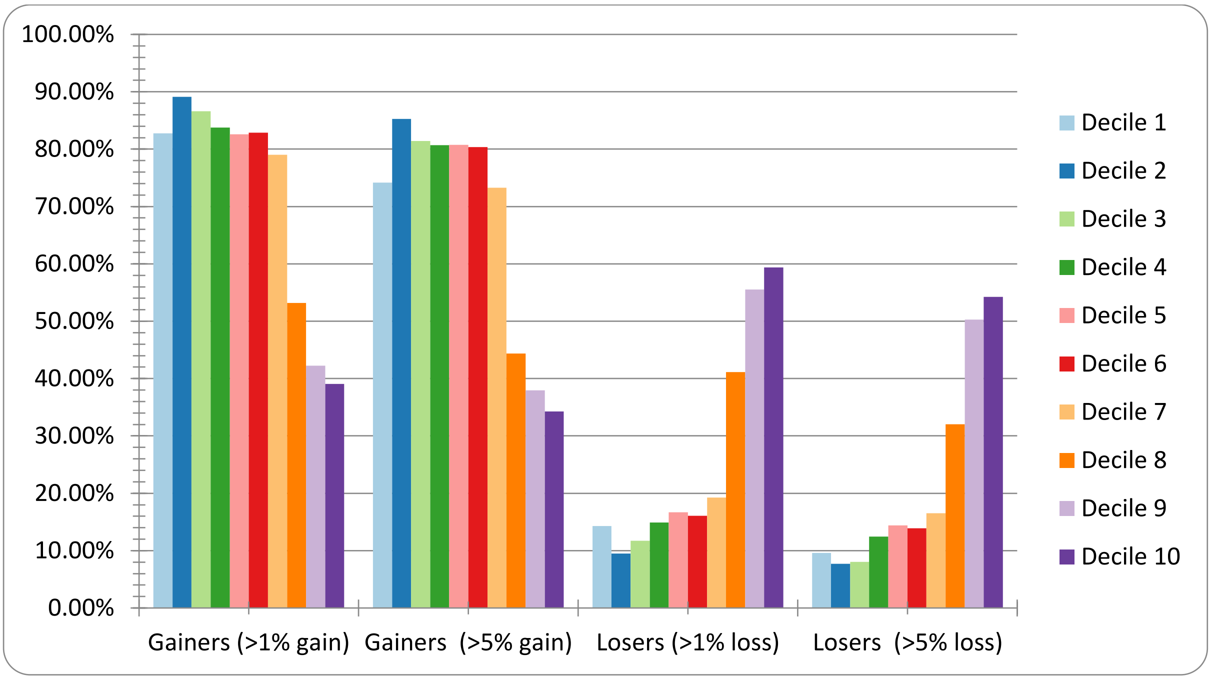

Additional results from primary analysis

Table F: Median income, prevalence of poverty, employment rate and mean hours worked in baseline scenario and three simulated Universal Basic Income (UBI) scenarios from 2022-2026 (95% uncertainty intervals)

Variable	Scenario	2022	2023	2024	2025	2026
**Median annual income (£)**	Baseline	21345.25 (20927.66, 21736.35)	22577.72 (22110.97, 23014.91)	23211.57 (22779.28, 23754.39)	24057.68 (23530.69, 24644.04)	25017.80 (24488.96, 25533.61)
	Partial	21345.25 (20927.66, 21736.35)	27172.90 (26599.29, 27652.72)	27992.75 (27536.77, 28513.33)	28936.35 (28455.66, 29497.71)	30028.81 (29445.47, 30586.48)
	Full	21345.25 (20927.66, 21736.35)	27420.63 (27190.69, 27629.56)	27980.31 (27763.91, 28219.95)	28668.42 (28440.86, 28924.84)	29409.85 (29142.28, 29642.18)
	Full+	21345.25 (20927.66, 21736.35)	27719.13 (27474.30, 27922.25)	28289.46 (28059.58, 28488.10)	28947.66 (28753.55, 29145.77)	29675.30 (29472.07, 29904.67)
**Poverty (%)**	Baseline	11.72 (11.09, 12.41)	9.10 (8.47, 9.71)	9.30 (8.70, 9.93)	9.38 (8.86, 10.05)	9.60 (8.97, 10.23)
	Partial	11.72 (11.09, 12.41)	7.13 (6.62, 7.66)	6.86 (6.32, 7.44)	6.62 (6.07, 7.19)	6.52 (5.99, 7.10)
	Full	11.72 (11.09, 12.41)	0.02 (0.00, 0.05)	0.02 (0.00, 0.05)	0.03 (0.01, 0.07)	0.04 (0.01, 0.08)
	Full+	11.72 (11.09, 12.41)	0.01 (0.00, 0.03)	0.02 (0.00, 0.04)	0.02 (0.00, 0.05)	0.02 (0.00, 0.05)
**Employ-ment (%)**	Baseline	77.09 (76.13, 77.99)	78.93 (77.94, 79.86)	79.93 (78.96, 80.83)	80.78 (79.79, 81.65)	81.42 (80.48, 82.25)
	Partial	77.09 (76.13, 77.99)	79.38 (78.41, 80.24)	80.32 (79.32, 81.19)	81.01 (80.03, 81.91)	81.44 (80.44, 82.30)
	Full	77.09 (76.13, 77.99)	75.69 (74.44, 76.92)	76.47 (75.14, 77.79)	77.08 (75.76, 78.21)	77.42 (76.12, 78.69)
	Full+	77.09 (76.13, 77.99)	74.10 (72.62, 75.43)	74.84 (73.30, 76.21)	75.43 (73.86, 76.86)	75.81 (74.27, 77.26)
**Average weekly hrs worked**	Baseline	25.82 (25.43, 26.14)	26.35 (26.00, 26.71)	26.67 (26.28, 26.99)	26.92 (26.58, 27.25)	27.11 (26.78, 27.47)
	Partial	Difference:	-0.08 (-0.18, 0.03)	-0.09 (-0.45, 0.19)	-0.16 (-0.48, 0.13)	-0.25 (-0.59, 0.09)
	Full	Difference:	-1.76 (-2.06, -1.42)	-1.84 (-2.35, -1.41)	-1.92 (-2.42, -1.49)	-2.03 (-2.52, -1.53)
	Full+	Difference:	-2.30 (-2.70, -1.91)	-2.38 (-2.87, -1.92)	-2.45 (-3.06, -2.00)	-2.58 (-3.08, -2.04)
	n	12918 (12918, 12919)	12997 (12996, 12998)	13044 (13042, 13046)	13141 (13140, 13143)	13120 (13119, 13122)

Baseline = planned tax/benefit policies for United Kingdom. Partial UBI = UBI set at level of existing benefits. Full UBI = UBI set at level of Minimum Income Standard. Full+ UBI = Minimum Income Standard plus means-tested benefits for caring, childcare, disability, housing, limited capability for work.

Table G: Estimated prevalence of common mental disorders (CMD) and mental health inequalities in baseline scenario and three simulated Universal Basic Income (UBI) scenarios from 2022-2026 (95% uncertainty intervals)

Variable	Scenario	2022	2023	2024	2025	2026
**Prevalence of CMD (%)**	Baseline	20.33 (19.13, 21.53)	19.12 (17.91, 20.38)	18.71 (17.65, 19.96)	18.55 (17.52, 19.79)	18.54 (17.42, 19.79)
	Partial	20.33 (19.13, 21.53)	19.01 (17.83, 20.31)	18.70 (17.61, 19.89)	18.58 (17.53, 19.86)	18.58 (17.52, 19.86)
		Difference:	-0.10 (-0.24, 0.02)	-0.02 (-0.84, 0.74)	0.01 (-0.78, 0.90)	0.04 (-0.79, 0.82)
	Full	20.33 (19.13, 21.53)	19.32 (18.09, 20.61)	18.80 (17.66, 20.11)	18.63 (17.53, 19.85)	18.58 (17.40, 19.85)
		Difference:	0.19 (-0.03, 0.47)	0.09 (-0.76, 0.91)	0.06 (-0.77, 0.84)	0.05 (-0.87, 0.87)
	Full+	20.33 (19.13, 21.53)	19.51 (18.31, 20.84)	18.86 (17.74, 20.12)	18.63 (17.56, 19.93)	18.61 (17.46, 19.85)
		Difference:	0.38 (0.13, 0.69)	0.16 (-0.78, 0.94)	0.08 (-0.73, 0.89)	0.08 (-0.78, 0.94)
**Relative index of inequality**	Baseline	1.33 (1.14, 1.55)	1.33 (1.13, 1.56)	1.29 (1.09, 1.52)	1.23 (1.04, 1.46)	1.20 (1.01, 1.42)
	Partial	1.33 (1.14, 1.55)	1.32 (1.12, 1.55)	1.29 (1.09, 1.51)	1.23 (1.05, 1.47)	1.20 (1.01, 1.44)
		Difference:	-0.01 (-0.04, 0.03)	-0.00 (-0.24, 0.25)	0.01 (-0.25, 0.26)	0.00 (-0.24, 0.24)
	Full	1.33 (1.14, 1.55)	1.30 (1.10, 1.52)	1.28 (1.08, 1.52)	1.23 (1.04, 1.45)	1.19 (1.00, 1.41)
		Difference:	-0.03 (-0.08, 0.02)	-0.01 (-0.24, 0.23)	-0.00 (-0.25, 0.24)	-0.00 (-0.24, 0.23)
	Full+	1.33 (1.14, 1.55)	1.30 (1.10, 1.52)	1.28 (1.07, 1.50)	1.22 (1.04, 1.43)	1.19 (1.00, 1.40)
		Difference:	-0.03 (-0.09, 0.02)	-0.01 (-0.26, 0.22)	-0.01 (-0.27, 0.24)	-0.01 (-0.25, 0.24)
**Slope index of inequality**	Baseline	0.06 (0.03, 0.09)	0.05 (0.02, 0.09)	0.05 (0.02, 0.08)	0.04 (0.01, 0.07)	0.03 (0.00, 0.06)
	Partial	0.06 (0.03, 0.09)	0.05 (0.02, 0.08)	0.05 (0.02, 0.08)	0.04 (0.01, 0.07)	0.03 (0.00, 0.07)
		Difference:	-0.00 (-0.01, 0.00)	-0.00 (-0.04, 0.03)	0.00 (-0.04, 0.04)	0.00 (-0.04, 0.04)
	Full	0.06 (0.03, 0.09)	0.05 (0.02, 0.08)	0.05 (0.01, 0.08)	0.04 (0.01, 0.07)	0.03 (-0.00, 0.06)
		Difference:	-0.00 (-0.01, 0.00)	-0.00 (-0.03, 0.03)	-0.00 (-0.04, 0.04)	-0.00 (-0.04, 0.04)
	Full+	0.06 (0.03, 0.09)	0.05 (0.02, 0.08)	0.05 (0.01, 0.08)	0.04 (0.01, 0.07)	0.03 (0.00, 0.06)
		Difference:	-0.00 (-0.01, 0.00)	-0.00 (-0.04, 0.03)	-0.00 (-0.04, 0.04)	-0.00 (-0.04, 0.04)

Baseline = planned tax/benefit policies for United Kingdom. Partial UBI = UBI set at level of existing benefits. Full UBI = UBI set at level of Minimum Income Standard. Full+ UBI = Minimum Income Standard plus means-tested benefits for caring, childcare, disability, housing, limited capability for work.

Figure G: Estimated relative (left panel) and slope (right panel) indices of inequality by education for common mental disorder (CMD) in modelled Universal Basic Income (UBI) policies from 2022-2026

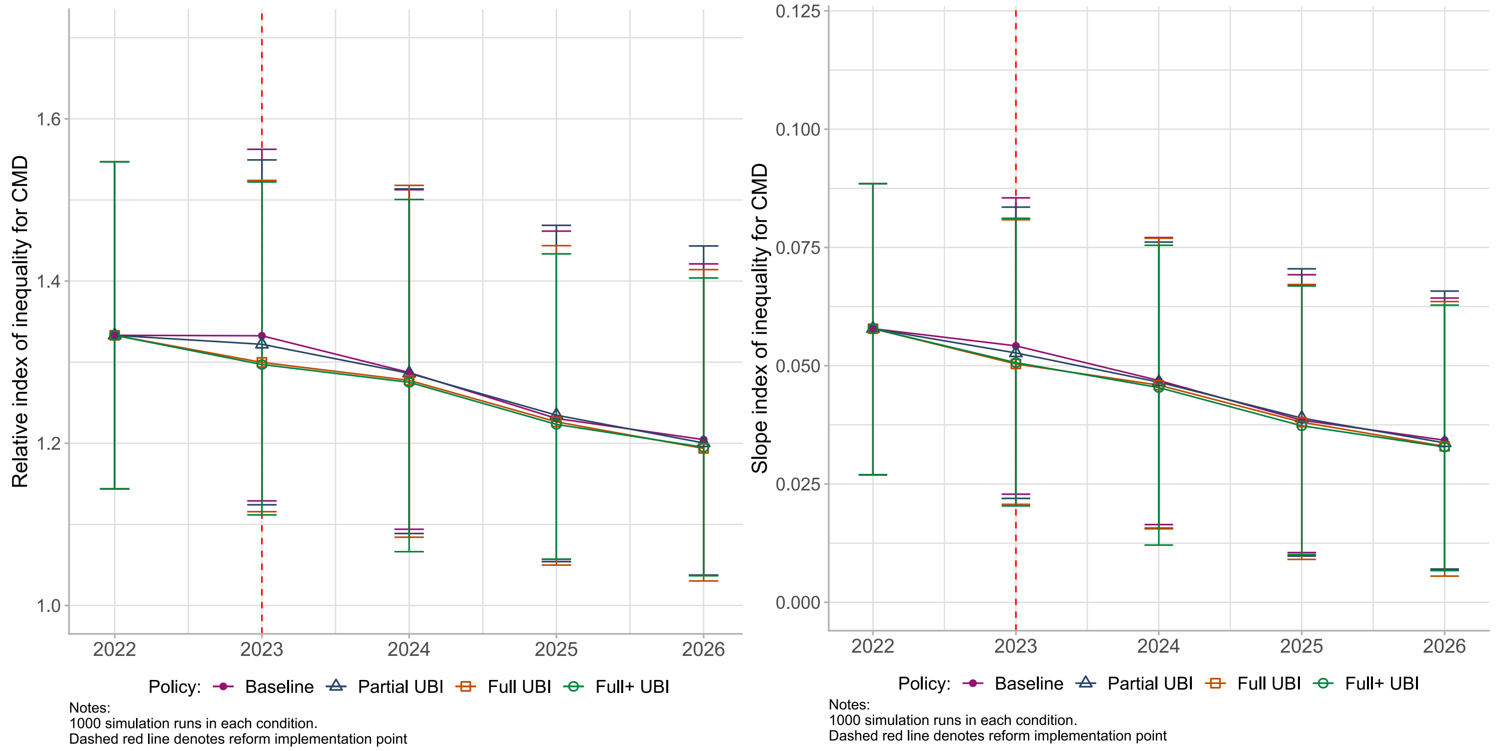

Baseline = planned tax/benefit policies for United Kingdom. Partial UBI = UBI set at level of existing benefits. Full UBI = UBI set at level of Minimum Income Standard. Full+ UBI = Minimum Income Standard plus means-tested benefits for caring, childcare, disability, housing, limited capability for work. Whiskers = 95% Uncertainty intervals.

Table H: Estimated prevalence of common mental disorders (%) in baseline scenario and three simulated Universal Basic Income (UBI) scenarios from 2022-2026 stratified by gender, education, age, and household structure (95% uncertainty intervals)

**Subgroup**	**Scenario**	**2022**	**2023**	**2024**	**2025**	**2026**
**Men**	Baseline	17.73 (16.33, 19.21)	16.35 (15.02, 17.81)	16.03 (14.67, 17.46)	15.92 (14.69, 17.33)	15.96 (14.65, 17.32)
	Partial	17.73 (16.33, 19.21)	16.28 (14.95, 17.68)	15.99 (14.69, 17.38)	15.97 (14.81, 17.50)	16.04 (14.68, 17.40)
		Difference:	-0.06 (-0.18, 0.05)	-0.03 (-1.19, 1.12)	0.05 (-1.13, 1.25)	0.05 (-1.11, 1.17)
	Full	17.73 (16.33, 19.21)	16.78 (15.38, 18.27)	16.26 (15.01, 17.73)	16.13 (14.89, 17.65)	16.17 (14.85, 17.64)
		Difference:	0.42 (0.12, 0.74)	0.23 (-0.93, 1.34)	0.23 (-1.02, 1.38)	0.18 (-0.94, 1.34)
	Full+	17.73 (16.33, 19.21)	16.98 (15.55, 18.53)	16.35 (15.08, 17.84)	16.21 (14.82, 17.76)	16.24 (14.91, 17.72)
		Difference:	0.63 (0.31, 1.01)	0.37 (-0.83, 1.50)	0.29 (-0.91, 1.41)	0.29 (-0.90, 1.41)
	n	6446 (6446, 6446)	6503 (6502, 6504)	6542 (6542, 6543)	6583 (6582, 6584)	6581 (6581, 6581)
**Women**	Baseline	22.93 (21.28, 24.60)	21.90 (20.37, 23.68)	21.44 (19.95, 23.09)	21.20 (19.79, 22.93)	21.12 (19.64, 22.79)
	Partial	22.93 (21.28, 24.60)	21.76 (20.26, 23.48)	21.44 (19.85, 23.08)	21.20 (19.64, 22.84)	21.13 (19.63, 22.82)

		Difference:	-0.14 (-0.38, 0.08)	-0.02 (-1.25, 1.14)	-0.02 (-1.30, 1.34)	0.02 (-1.32, 1.28)
	Full	22.93 (21.28, 24.60)	21.87 (20.30, 23.70)	21.35 (19.78, 23.17)	21.10 (19.62, 22.81)	21.02 (19.26, 22.62)
		Difference:	-0.03 (-0.37, 0.35)	-0.09 (-1.32, 1.14)	-0.11 (-1.46, 1.14)	-0.14 (-1.50, 1.13)
	Full+	22.93 (21.28, 24.60)	22.00 (20.47, 23.88)	21.36 (19.84, 23.08)	21.10 (19.58, 22.80)	20.97 (19.37, 22.68)
		Difference:	0.14 (-0.23, 0.54)	-0.05 (-1.32, 1.22)	-0.11 (-1.40, 1.10)	-0.15 (-1.53, 1.24)
	n	6472 (6472, 6473)	6494 (6494, 6494)	6502 (6500, 6503)	6558 (6558, 6559)	6539 (6538, 6541)
**Low education**	Baseline	23.23 (20.66, 25.83)	21.91 (19.39, 24.77)	21.09 (18.41, 23.76)	20.39 (17.84, 23.08)	20.23 (17.49, 23.02)
	Partial	23.23 (20.66, 25.83)	21.77 (19.20, 24.63)	20.90 (18.44, 23.52)	20.38 (17.83, 23.37)	20.27 (17.48, 23.32)
		Difference:	-0.15 (-0.58, 0.28)	-0.08 (-2.75, 2.89)	0.05 (-2.90, 2.96)	0.18 (-3.21, 3.42)
	Full	23.23 (20.66, 25.83)	21.92 (19.32, 24.83)	21.06 (18.59, 23.95)	20.43 (17.80, 23.34)	20.26 (17.45, 23.06)
		Difference:	0.00 (-0.63, 0.65)	0.08 (-3.00, 3.04)	0.12 (-3.05, 3.05)	0.10 (-3.09, 3.20)
	Full+	23.23 (20.66, 25.83)	22.16 (19.60, 25.14)	21.07 (18.40, 23.84)	20.47 (17.78, 23.33)	20.21 (17.65, 23.12)
		Difference:	0.23 (-0.36, 0.90)	0.08 (-2.80, 3.00)	0.10 (-3.10, 3.05)	0.12 (-3.01, 3.35)
	n	1424 (1342, 1509)	1360 (1278, 1445)	1288 (1205, 1376)	1220 (1139, 1307)	1130 (1048, 1215)
**Medium education**	Baseline	20.69 (19.21, 22.09)	19.34 (17.87, 20.78)	18.91 (17.53, 20.33)	18.72 (17.38, 20.16)	18.66 (17.38, 20.11)
	Partial	20.69 (19.21, 22.09)	19.22 (17.74, 20.68)	18.87 (17.62, 20.26)	18.76 (17.44, 20.16)	18.69 (17.46, 20.07)
		Difference:	-0.12 (-0.30, 0.06)	-0.04 (-1.17, 1.15)	0.06 (-1.13, 1.22)	0.03 (-1.14, 1.18)
	Full	20.69 (19.21, 22.09)	19.48 (18.00, 21.05)	18.96 (17.68, 20.46)	18.72 (17.44, 20.17)	18.66 (17.31, 20.06)
		Difference:	0.16 (-0.14, 0.48)	0.06 (-1.08, 1.26)	0.01 (-1.12, 1.21)	0.01 (-1.15, 1.22)
	Full+	20.69 (19.21, 22.09)	19.67 (18.17, 21.29)	19.03 (17.61, 20.46)	18.75 (17.44, 20.20)	18.70 (17.32, 20.16)
		Difference:	0.34 (0.01, 0.72)	0.14 (-1.06, 1.25)	0.07 (-1.19, 1.29)	0.05 (-1.15, 1.25)
	n	6841 (6701, 6991)	6906 (6763, 7052)	6998 (6853, 7143)	7075 (6924, 7224)	7107 (6949, 7254)
**High education**	Baseline	18.94 (17.40, 20.50)	17.99 (16.55, 19.63)	17.85 (16.40, 19.53)	17.89 (16.51, 19.44)	18.02 (16.50, 19.58)
	Partial	18.94 (17.40, 20.50)	17.95 (16.48, 19.56)	17.84 (16.32, 19.54)	17.93 (16.34, 19.46)	18.06 (16.59, 19.60)
		Difference:	-0.06 (-0.30, 0.15)	-0.02 (-1.46, 1.39)	-0.02 (-1.50, 1.50)	0.01 (-1.37, 1.47)
	Full	18.94 (17.40, 20.50)	18.32 (16.82, 20.01)	17.98 (16.48, 19.62)	18.02 (16.46, 19.60)	18.04 (16.55, 19.71)
		Difference:	0.31 (-0.04, 0.68)	0.13 (-1.29, 1.37)	0.09 (-1.34, 1.47)	0.07 (-1.46, 1.49)
	Full+	18.94 (17.40, 20.50)	18.50 (16.99, 20.18)	18.01 (16.49, 19.65)	18.02 (16.53, 19.60)	18.10 (16.62, 19.67)
		Difference:	0.48 (0.13, 0.91)	0.17 (-1.24, 1.58)	0.12 (-1.27, 1.57)	0.10 (-1.38, 1.57)

	n	4653 (4518, 4798)	4733 (4596, 4876)	4758 (4618, 4903)	4847 (4704, 4991)	4884 (4737, 5035)
**Age group: 25-44 years**	Baseline	20.59 (19.18, 21.98)	19.98 (18.48, 21.51)	19.76 (18.39, 21.28)	19.63 (18.32, 21.19)	19.69 (18.35, 21.20)
	Partial	20.59 (19.18, 21.98)	19.88 (18.45, 21.34)	19.79 (18.42, 21.27)	19.69 (18.43, 21.13)	19.70 (18.37, 21.13)
		Difference:	-0.09 (-0.26, 0.07)	0.00 (-1.12, 1.14)	0.06 (-1.14, 1.21)	0.07 (-1.18, 1.20)
	Full	20.59 (19.18, 21.98)	20.23 (18.79, 21.79)	19.89 (18.45, 21.40)	19.69 (18.30, 21.22)	19.71 (18.32, 21.22)
		Difference:	0.26 (-0.04, 0.60)	0.12 (-1.07, 1.34)	0.06 (-1.14, 1.25)	0.06 (-1.20, 1.20)
	Full+	20.59 (19.18, 21.98)	20.34 (18.92, 21.88)	19.95 (18.62, 21.52)	19.69 (18.42, 21.25)	19.75 (18.19, 21.22)
		Difference:	0.35 (0.03, 0.71)	0.18 (-1.02, 1.31)	0.12 (-1.10, 1.21)	0.06 (-1.18, 1.21)
	n	6721 (6721, 6721)	6801 (6801, 6801)	6855 (6853, 6856)	6933 (6933, 6935)	6928 (6927, 6930)
**Age group: 45-64 years**	Baseline	20.02 (18.70, 21.53)	18.17 (16.82, 19.61)	17.56 (16.29, 18.99)	17.41 (16.14, 18.70)	17.30 (16.00, 18.69)
	Partial	20.02 (18.70, 21.53)	18.06 (16.75, 19.47)	17.51 (16.22, 18.90)	17.40 (16.06, 18.65)	17.32 (16.10, 18.59)
		Difference:	-0.11 (-0.32, 0.08)	-0.06 (-1.23, 1.16)	0.00 (-1.29, 1.32)	0.02 (-1.16, 1.24)
	Full	20.02 (18.70, 21.53)	18.30 (16.92, 19.72)	17.58 (16.36, 19.02)	17.45 (16.11, 18.83)	17.38 (15.96, 18.67)
		Difference:	0.12 (-0.19, 0.44)	0.03 (-1.18, 1.28)	0.05 (-1.22, 1.22)	0.06 (-1.20, 1.32)
	Full+	20.02 (18.70, 21.53)	18.60 (17.19, 20.05)	17.71 (16.35, 19.11)	17.48 (16.11, 18.75)	17.36 (15.99, 18.78)
		Difference:	0.42 (0.08, 0.81)	0.10 (-1.10, 1.29)	0.06 (-1.24, 1.31)	0.05 (-1.11, 1.34)
	n	6197 (6197, 6198)	6196 (6195, 6197)	6189 (6189, 6190)	6208 (6207, 6208)	6192 (6192, 6192)
**Have children**	Baseline	20.28 (18.53, 21.85)	19.87 (18.10, 22.05)	19.71 (17.79, 22.02)	19.62 (17.67, 21.88)	19.55 (17.50, 21.78)
	Partial	20.28 (18.53, 21.85)	19.81 (17.98, 22.00)	19.71 (17.73, 22.03)	19.61 (17.59, 21.89)	19.53 (17.49, 21.90)
		Difference:	-0.07 (-0.32, 0.18)	-0.03 (-1.37, 1.43)	-0.00 (-1.61, 1.54)	0.04 (-1.45, 1.51)
	Full	20.28 (18.53, 21.85)	20.35 (18.51, 22.60)	19.90 (17.89, 22.26)	19.68 (17.64, 21.91)	19.51 (17.44, 21.82)
		Difference:	0.47 (0.04, 0.91)	0.16 (-1.28, 1.64)	0.07 (-1.59, 1.61)	0.04 (-1.54, 1.46)
	Full+	20.28 (18.53, 21.85)	20.51 (18.61, 22.81)	19.89 (17.97, 22.28)	19.74 (17.72, 21.96)	19.55 (17.63, 21.99)
		Difference:	0.64 (0.18, 1.14)	0.15 (-1.24, 1.68)	0.08 (-1.38, 1.55)	0.06 (-1.51, 1.49)
	n	4464 (4352, 4573)	4482 (4368, 4589)	4566 (4466, 4671)	4649 (4547, 4752)	4720 (4615, 4824)
**No children**	Baseline	20.36 (18.95, 21.69)	18.71 (17.50, 19.93)	18.19 (17.09, 19.44)	18.03 (16.84, 19.17)	18.00 (16.83, 19.24)
	Partial	20.36 (18.95, 21.69)	18.60 (17.38, 19.78)	18.15 (17.08, 19.39)	18.04 (16.92, 19.26)	18.03 (16.84, 19.21)
		Difference:	-0.13 (-0.28, 0.02)	-0.01 (-1.06, 1.01)	0.03 (-1.05, 1.07)	0.04 (-1.00, 1.02)
	Full	20.36 (18.95, 21.69)	18.77 (17.48, 19.98)	18.23 (17.05, 19.42)	18.05 (16.90, 19.26)	18.07 (16.85, 19.25)
		Difference:	0.06 (-0.21, 0.32)	0.04 (-1.00, 1.05)	0.06 (-1.07, 1.09)	0.05 (-1.07, 1.13)

	Full+	20.36 (18.95, 21.69)	18.97 (17.69, 20.23)	18.34 (17.14, 19.42)	18.09 (17.00, 19.29)	18.04 (16.92, 19.31)
		Difference:	0.26 (-0.04, 0.56)	0.14 (-0.92, 1.19)	0.08 (-0.91, 1.04)	0.07 (-0.91, 1.12)
	n	8454 (8345, 8566)	8515 (8407, 8628)	8478 (8372, 8578)	8491 (8390, 8593)	8400 (8295, 8504)
**Lone parent**	Baseline	29.83 (24.61, 35.03)	27.73 (22.64, 32.61)	27.08 (22.06, 32.22)	26.50 (21.36, 32.20)	25.95 (21.05, 31.22)
	Partial	29.83 (24.61, 35.03)	27.44 (22.39, 32.21)	27.03 (22.17, 32.39)	26.63 (21.93, 32.02)	26.14 (21.09, 31.45)
		Difference:	-0.24 (-1.27, 0.98)	-0.08 (-5.53, 5.27)	0.07 (-6.18, 6.32)	0.17 (-5.74, 6.12)
	Full	29.83 (24.61, 35.03)	27.71 (22.74, 32.56)	26.86 (22.17, 32.47)	26.17 (21.30, 31.30)	25.56 (20.84, 30.73)
		Difference:	0.00 (-1.05, 1.03)	-0.28 (-5.87, 5.39)	-0.30 (-6.74, 5.55)	-0.36 (-6.34, 5.70)
	Full+	29.83 (24.61, 35.03)	27.99 (22.89, 32.81)	26.96 (22.25, 32.27)	26.20 (21.55, 31.40)	25.61 (20.94, 31.14)
		Difference:	0.24 (-0.77, 1.30)	-0.04 (-5.90, 5.64)	-0.22 (-6.66, 6.24)	-0.23 (-6.82, 5.91)
	n	435 (391, 480)	411 (368, 461)	404 (359, 455)	392 (348, 445)	381 (333, 437)

Baseline = planned tax/benefit policies for United Kingdom. Partial UBI = UBI set at level of existing benefits. Full UBI = UBI set at level of Minimum Income Standard. Full+ UBI = Minimum Income Standard plus means-tested benefits for caring, childcare, disability, housing, limited capability for work. Low education = no formal qualifications; medium education = Higher/A-level/GCSE [General Certificate of Secondary Education] or equivalent; high education = degree or equivalent.

Results from structural sensitivity analyses

Figure H: Structural Sensitivity Analysis 1, relaxing employment assumptions – Estimates of poverty (left panel) and employment (right panel) for modelled Universal Basic Income scenarios from 2022-2026

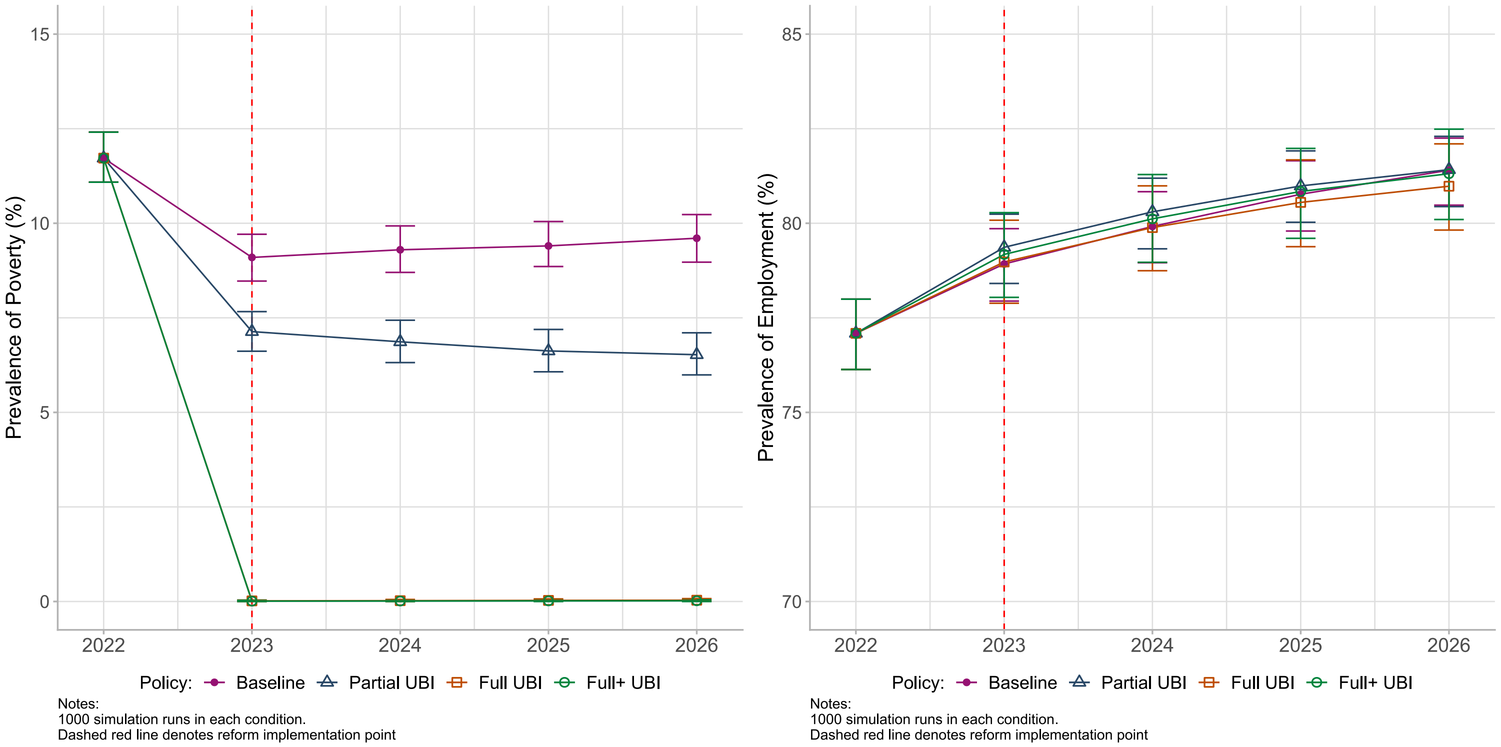

Baseline = planned tax/benefit policies for United Kingdom. Partial UBI = UBI set at level of existing benefits. Full UBI = UBI set at level of Minimum Income Standard. Full+ UBI = Minimum Income Standard plus means-tested benefits for caring, childcare, disability, housing, limited capability for work. Whiskers = 95% Uncertainty intervals.

Table I: Structural Sensitivity Analyses – Median income, prevalence of poverty, employment rate and mean hours worked in baseline scenario and three simulated Universal Basic Income (UBI) scenarios from 2022-2026 (95% uncertainty intervals)

Variable	Scenario	2022	2023	2024	2025	2026
**STRUCTURAL SENSITIVITY ANALYSIS 1: RELAXING EMPLOYMENT ASSUMPTIONS**						
**Median annual income (£)**	Baseline	21345.25 (20927.66, 21736.35)	22577.72 (22110.97, 23014.91)	23211.57 (22779.28, 23754.39)	24057.68 (23530.69, 24644.04)	25017.80 (24488.96, 25533.61)
	Partial	21345.25 (20927.66, 21736.35)	27172.90 (26599.29, 27652.72)	27992.75 (27536.77, 28513.33)	28936.35 (28455.66, 29497.71)	30028.81 (29445.47, 30586.48)
	Full	21345.25 (20927.66, 21736.35)	27843.91 (27626.30, 28042.66)	28426.52 (28212.66, 28635.21)	29105.64 (28904.20, 29330.95)	29862.92 (29635.19, 30124.47)
	Full+	21345.25 (20927.66, 21736.35)	28263.90 (28064.08, 28467.93)	28851.15 (28645.65, 29065.77)	29539.17 (29324.70, 29765.52)	30296.44 (30055.57, 30548.88)
**Poverty (%)**	Baseline	11.72 (11.09, 12.41)	9.10 (8.47, 9.71)	9.30 (8.70, 9.93)	9.38 (8.86, 10.05)	9.60 (8.97, 10.23)
	Partial	11.72 (11.09, 12.41)	7.13 (6.62, 7.66)	6.86 (6.32, 7.44)	6.62 (6.07, 7.19)	6.52 (5.99, 7.10)

	Full	11.72 (11.09, 12.41)	0.02 (0.00, 0.04)	0.02 (0.00, 0.05)	0.03 (0.01, 0.07)	0.03 (0.01, 0.08)
	Full+	11.72 (11.09, 12.41)	0.01 (0.00, 0.03)	0.01 (0.00, 0.03)	0.02 (0.00, 0.04)	0.02 (0.00, 0.05)
**Employ-ment (%)**	Baseline	77.09 (76.13, 77.99)	78.93 (77.94, 79.86)	79.93 (78.96, 80.83)	80.78 (79.79, 81.65)	81.42 (80.48, 82.25)
	Partial	77.09 (76.13, 77.99)	79.38 (78.41, 80.24)	80.32 (79.32, 81.19)	81.01 (80.03, 81.91)	81.44 (80.44, 82.30)
	Full	77.09 (76.13, 77.99)	78.99 (77.89, 80.08)	79.90 (78.75, 80.99)	80.58 (79.38, 81.68)	80.98 (79.82, 82.10)
	Full+	77.09 (76.13, 77.99)	79.19 (78.04, 80.28)	80.12 (78.97, 81.29)	80.86 (79.60, 81.98)	81.32 (80.10, 82.49)
**Average weekly hrs worked**	Baseline	25.82 (25.43, 26.14)	26.35 (26.00, 26.71)	26.67 (26.28, 26.99)	26.92 (26.58, 27.25)	27.11 (26.78, 27.47)
	Partial	Difference:	-0.08 (-0.18, 0.03)	-0.09 (-0.45, 0.19)	-0.16 (-0.48, 0.13)	-0.25 (-0.59, 0.09)
	Full	Difference:	-0.12 (-0.58, 0.29)	-0.15 (-0.72, 0.29)	-0.21 (-0.80, 0.33)	-0.26 (-0.88, 0.23)
	Full+	Difference:	0.18 (-0.31, 0.58)	0.16 (-0.39, 0.68)	0.14 (-0.49, 0.67)	0.07 (-0.54, 0.62)
	n	12918 (12918, 12919)	12997 (12996, 12998)	13044 (13042, 13046)	13141 (13140, 13143)	13120 (13119, 13122)

Note: Not shown for Structural Sensitivity Analysis 2, as these results are identical to primary analysis. Baseline = planned tax/benefit policies for United Kingdom. Partial UBI = UBI set at level of existing benefits. Full UBI = UBI set at level of Minimum Income Standard. Full+ UBI = Minimum Income Standard plus means-tested benefits for caring, childcare, disability, housing, limited capability for work.

Figure I: Structural Sensitivity Analysis 1, relaxing employment assumptions - Estimated prevalence of common mental disorder (CMD) for modelled Universal Basic Income (UBI) policies from 2022-2026

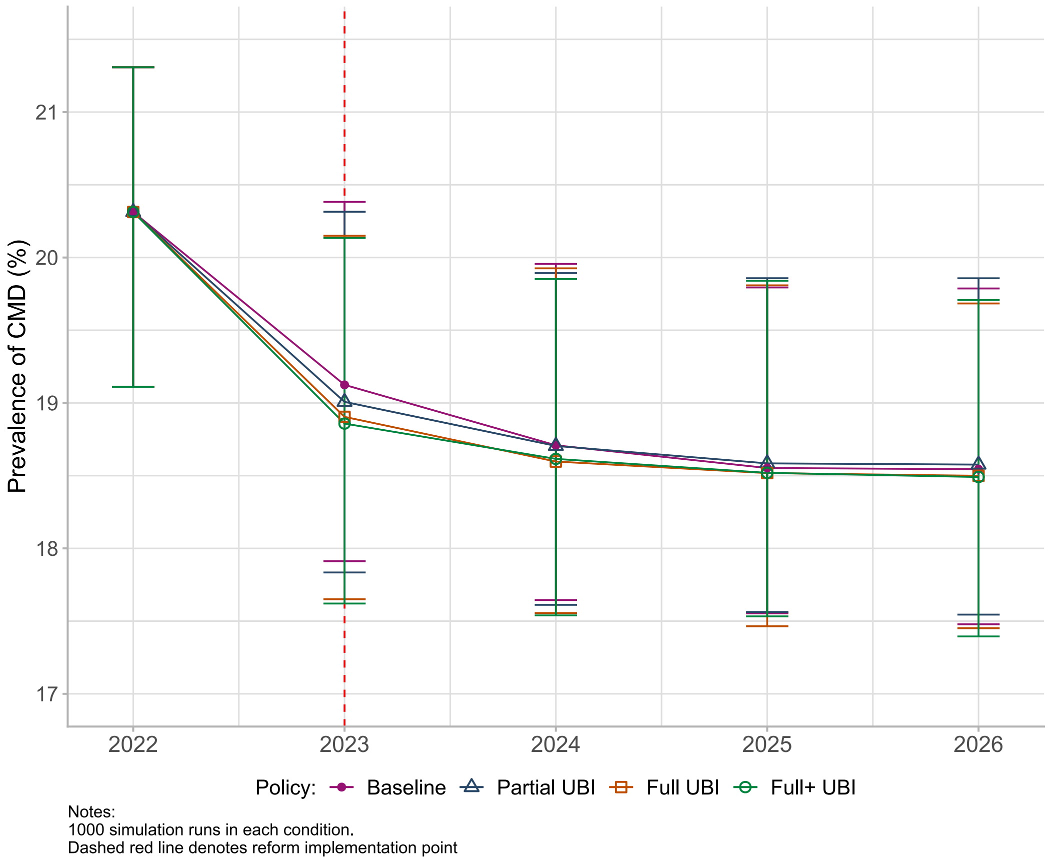

Baseline = planned tax/benefit policies for United Kingdom. Partial UBI = UBI set at level of existing benefits. Full UBI = UBI set at level of Minimum Income Standard. Full+ UBI = Minimum Income Standard plus means-tested benefits for caring, childcare, disability, housing, limited capability for work. Whiskers = 95% Uncertainty intervals.

<u>Figure J: Structural Sensitivity Analysis 2, using economic inactivity effects - Estimated prevalence of common mental disorder (CMD) for modelled Universal Basic Income (UBI) policies from 2022-2026</u>

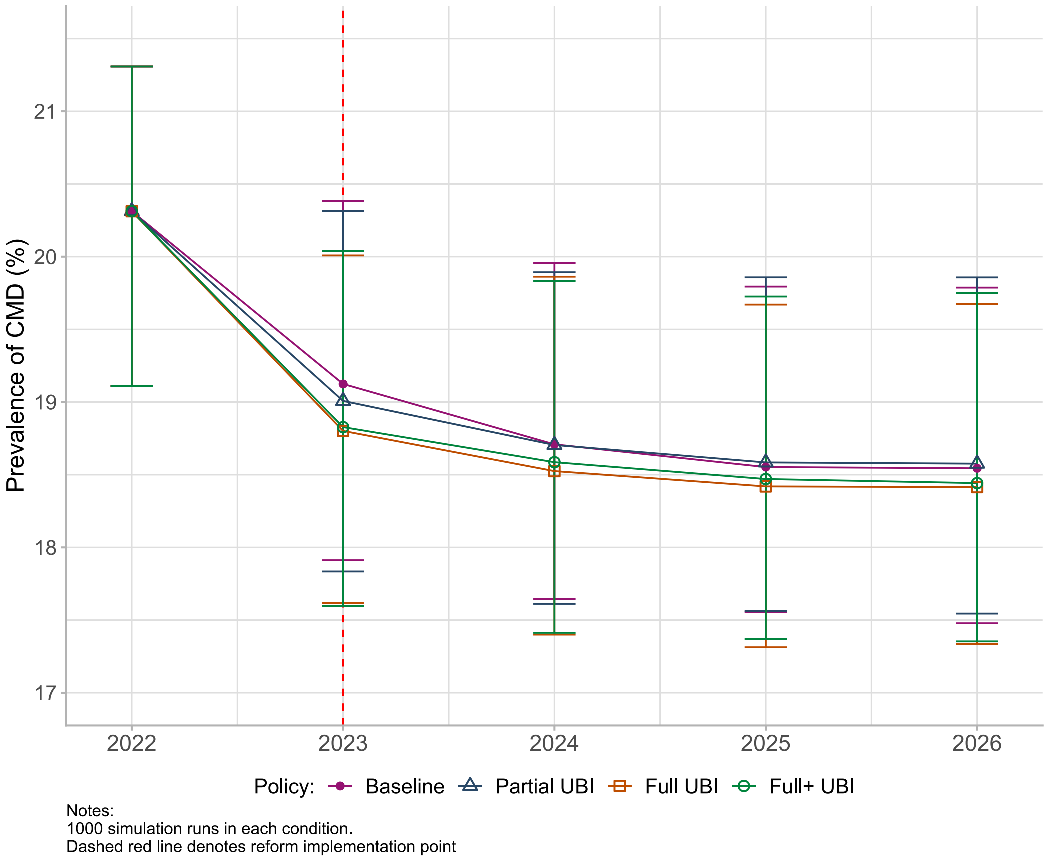

Baseline = planned tax/benefit policies for United Kingdom. Partial UBI = UBI set at level of existing benefits. Full UBI = UBI set at level of Minimum Income Standard. Full+ UBI = Minimum Income Standard plus means-tested benefits for caring, childcare, disability, housing, limited capability for work. Whiskers = 95% Uncertainty intervals.

<u>Table J: Structural Sensitivity Analyses – Estimated prevalence of common mental disorders and mental health inequalities in baseline scenario and three simulated Universal Basic Income (UBI) scenarios from 2022-2026 (95% uncertainty intervals)</u>

Variable	Scenario	2022	2023	2024	2025	2026
**STRUCTURAL SENSITIVITY ANALYSIS 1: RELAXING EMPLOYMENT ASSUMPTIONS**						
**Prevalence of CMD (%)**	Baseline	20.33 (19.13, 21.53)	19.12 (17.91, 20.38)	18.71 (17.65, 19.96)	18.55 (17.52, 19.79)	18.54 (17.42, 19.79)
	Partial	20.33 (19.13, 21.53)	19.01 (17.83, 20.31)	18.70 (17.61, 19.89)	18.58 (17.53, 19.86)	18.58 (17.52, 19.86)
		Difference:	-0.10 (-0.24, 0.02)	-0.02 (-0.84, 0.74)	0.01 (-0.78, 0.90)	0.04 (-0.79, 0.82)
	Full	20.33 (19.13, 21.53)	18.90 (17.65, 20.15)	18.60 (17.55, 19.93)	18.52 (17.45, 19.81)	18.50 (17.44, 19.68)
		Difference:	-0.23 (-0.45, -0.01)	-0.09 (-0.94, 0.70)	0.00 (-0.93, 0.81)	-0.06 (-0.92, 0.80)
	Full+	20.33 (19.13, 21.53)	18.86 (17.62, 20.13)	18.62 (17.54, 19.85)	18.52 (17.52, 19.84)	18.49 (17.39, 19.71)

		Difference:	-0.27 (-0.49, -0.05)	-0.11 (-0.88, 0.69)	-0.02 (-0.88, 0.72)	-0.06 (-0.95, 0.76)
**Relative index of inequality**	Baseline	1.33 (1.14, 1.55)	1.33 (1.13, 1.56)	1.29 (1.09, 1.52)	1.23 (1.04, 1.46)	1.20 (1.01, 1.42)
	Partial	1.33 (1.14, 1.55)	1.32 (1.12, 1.55)	1.29 (1.09, 1.51)	1.23 (1.05, 1.47)	1.20 (1.01, 1.44)
		Difference:	-0.01 (-0.04, 0.03)	-0.00 (-0.24, 0.25)	0.01 (-0.25, 0.26)	0.00 (-0.24, 0.24)
	Full	1.33 (1.14, 1.55)	1.31 (1.10, 1.54)	1.27 (1.08, 1.51)	1.23 (1.03, 1.45)	1.20 (1.01, 1.42)
		Difference:	-0.02 (-0.07, 0.03)	-0.01 (-0.24, 0.22)	-0.00 (-0.27, 0.24)	-0.00 (-0.24, 0.26)
	Full+	1.33 (1.14, 1.55)	1.31 (1.10, 1.53)	1.27 (1.08, 1.52)	1.23 (1.02, 1.46)	1.19 (1.01, 1.43)
		Difference:	-0.02 (-0.06, 0.03)	-0.01 (-0.25, 0.23)	-0.00 (-0.26, 0.25)	-0.01 (-0.24, 0.23)
**Slope index of inequality**	Baseline	0.06 (0.03, 0.09)	0.05 (0.02, 0.09)	0.05 (0.02, 0.08)	0.04 (0.01, 0.07)	0.03 (0.00, 0.06)
	Partial	0.06 (0.03, 0.09)	0.05 (0.02, 0.08)	0.05 (0.02, 0.08)	0.04 (0.01, 0.07)	0.03 (0.00, 0.07)
		Difference:	-0.00 (-0.01, 0.00)	-0.00 (-0.04, 0.03)	0.00 (-0.04, 0.04)	0.00 (-0.04, 0.04)
	Full	0.06 (0.03, 0.09)	0.05 (0.02, 0.08)	0.05 (0.01, 0.08)	0.04 (0.01, 0.07)	0.03 (0.00, 0.06)
		Difference:	-0.00 (-0.01, 0.00)	-0.00 (-0.04, 0.03)	-0.00 (-0.04, 0.04)	-0.00 (-0.04, 0.04)
	Full+	0.06 (0.03, 0.09)	0.05 (0.02, 0.08)	0.04 (0.01, 0.08)	0.04 (0.00, 0.07)	0.03 (0.00, 0.06)
		Difference:	-0.00 (-0.01, 0.00)	-0.00 (-0.04, 0.03)	-0.00 (-0.04, 0.04)	-0.00 (-0.04, 0.03)
**STRUCTURAL SENSITIVITY ANALYSIS 2: USING ECONOMIC INACTIVITY EFFECTS IN FULL UBI YEARS**						
**Prevalence of CMD (%)**	Baseline	20.33 (19.13, 21.53)	19.12 (17.91, 20.38)	18.71 (17.65, 19.96)	18.55 (17.52, 19.79)	18.54 (17.42, 19.79)
	Partial	20.33 (19.13, 21.53)	19.01 (17.83, 20.31)	18.70 (17.61, 19.89)	18.58 (17.53, 19.86)	18.58 (17.52, 19.86)
		Difference:	-0.10 (-0.24, 0.02)	-0.02 (-0.84, 0.74)	0.01 (-0.78, 0.90)	0.04 (-0.79, 0.82)
	Full	20.33 (19.13, 21.53)	18.80 (17.60, 20.04)	18.52 (17.33, 19.86)	18.42 (17.27, 19.67)	18.41 (17.26, 19.67)
		Difference:	-0.34 (-0.95, 0.32)	-0.20 (-1.26, 0.81)	-0.15 (-1.21, 0.93)	-0.14 (-1.27, 1.14)
	Full+	20.33 (19.13, 21.53)	18.83 (17.60, 20.07)	18.58 (17.39, 19.83)	18.46 (17.31, 19.72)	18.44 (17.19, 19.75)
		Difference:	-0.31 (-0.93, 0.35)	-0.16 (-1.23, 0.82)	-0.13 (-1.23, 1.03)	-0.11 (-1.33, 1.11)
**Relative index of inequality**	Baseline	1.33 (1.14, 1.55)	1.33 (1.13, 1.56)	1.29 (1.09, 1.52)	1.23 (1.04, 1.46)	1.20 (1.01, 1.42)
	Partial	1.33 (1.14, 1.55)	1.32 (1.12, 1.55)	1.29 (1.09, 1.51)	1.23 (1.05, 1.47)	1.20 (1.01, 1.44)
		Difference:	-0.01 (-0.04, 0.03)	-0.00 (-0.24, 0.25)	0.01 (-0.25, 0.26)	0.00 (-0.24, 0.24)
	Full	1.33 (1.14, 1.55)	1.32 (1.10, 1.55)	1.29 (1.09, 1.52)	1.24 (1.04, 1.46)	1.21 (1.01, 1.42)
		Difference:	-0.01 (-0.10, 0.07)	0.00 (-0.24, 0.25)	0.01 (-0.24, 0.25)	0.00 (-0.24, 0.24)
	Full+	1.33 (1.14, 1.55)	1.32 (1.11, 1.55)	1.28 (1.08, 1.53)	1.23 (1.05, 1.45)	1.20 (1.01, 1.42)
		Difference:	-0.01 (-0.09, 0.07)	-0.00 (-0.24, 0.24)	-0.00 (-0.25, 0.25)	0.00 (-0.24, 0.24)
**Slope index of inequality**	Baseline	0.06 (0.03, 0.09)	0.05 (0.02, 0.09)	0.05 (0.02, 0.08)	0.04 (0.01, 0.07)	0.03 (0.00, 0.06)
	Partial	0.06 (0.03, 0.09)	0.05 (0.02, 0.08)	0.05 (0.02, 0.08)	0.04 (0.01, 0.07)	0.03 (0.00, 0.07)
		Difference:	-0.00 (-0.01, 0.00)	-0.00 (-0.04, 0.03)	0.00 (-0.04, 0.04)	0.00 (-0.04, 0.04)
	Full	0.06 (0.03, 0.09)	0.05 (0.02, 0.08)	0.05 (0.02, 0.08)	0.04 (0.01, 0.07)	0.03 (0.00, 0.06)
		Difference:	-0.00 (-0.01, 0.01)	-0.00 (-0.03, 0.03)	0.00 (-0.04, 0.04)	-0.00 (-0.04, 0.04)
	Full+	0.06 (0.03, 0.09)	0.05 (0.02, 0.08)	0.05 (0.01, 0.08)	0.04 (0.01, 0.07)	0.03 (0.00, 0.06)
		Difference:	-0.00 (-0.01, 0.01)	-0.00 (-0.03, 0.03)	-0.00 (-0.04, 0.04)	-0.00 (-0.04, 0.04)

Baseline = planned tax/benefit policies for United Kingdom. Partial UBI = UBI set at level of existing benefits. Full UBI = UBI set at level of Minimum Income Standard. Full+ UBI = Minimum Income Standard plus means-tested benefits for caring, childcare, disability, housing, limited capability for work.

Figure K: Structural Sensitivity Analysis 1, relaxing employment assumptions – Estimated relative (left panel) and slope (right panel) indices of inequality by education for common mental disorder (CMD) in modelled Universal Basic Income (UBI) policies from 2022-2026

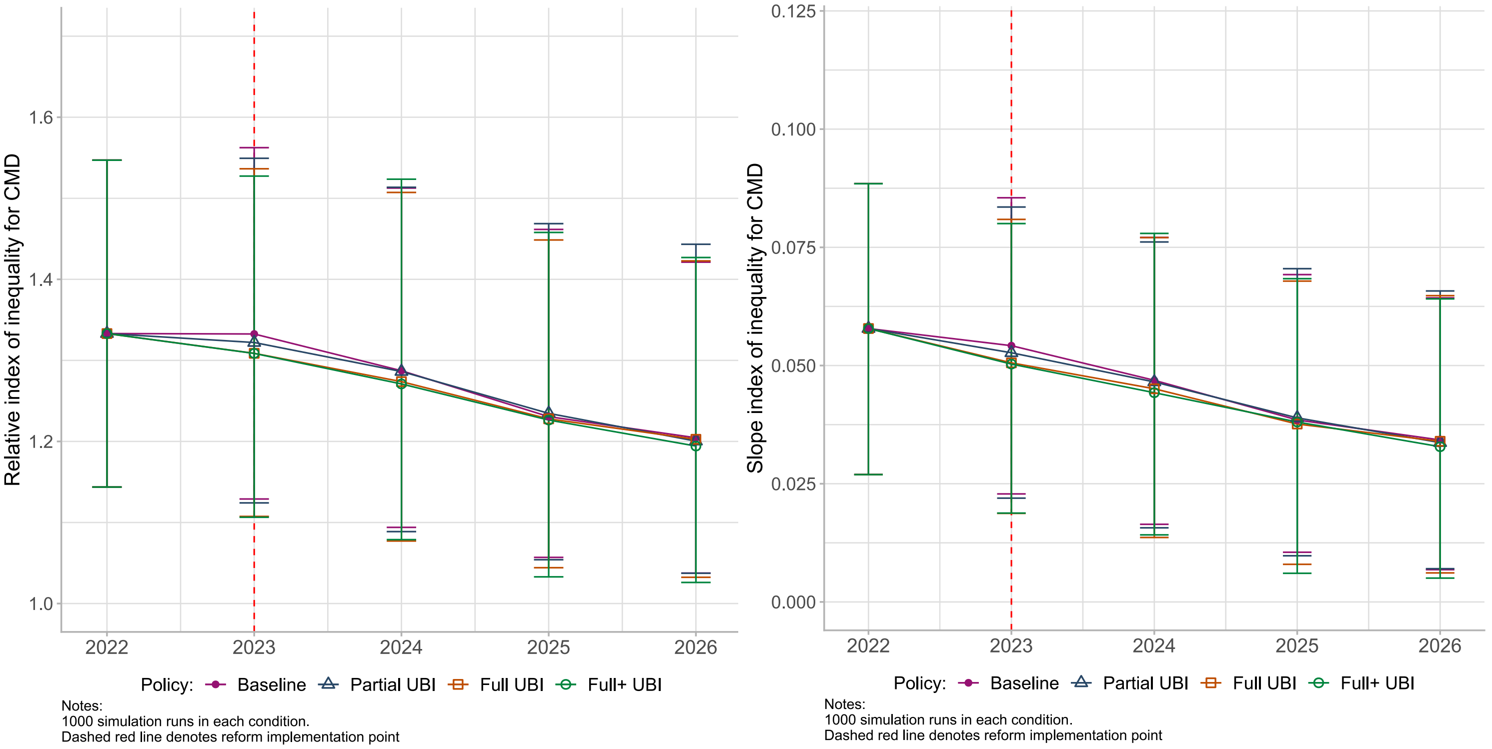

Baseline = planned tax/benefit policies for United Kingdom. Partial UBI = UBI set at level of existing benefits. Full UBI = UBI set at level of Minimum Income Standard. Full+ UBI = Minimum Income Standard plus means-tested benefits for caring, childcare, disability, housing, limited capability for work. Whiskers = 95% Uncertainty intervals.

Figure L: Structural Sensitivity Analysis 2, using economic inactivity effects – Estimated relative (left panel) and slope (right panel) indices of inequality by education for common mental disorder (CMD) in modelled Universal Basic Income (UBI) policies from 2022-2026

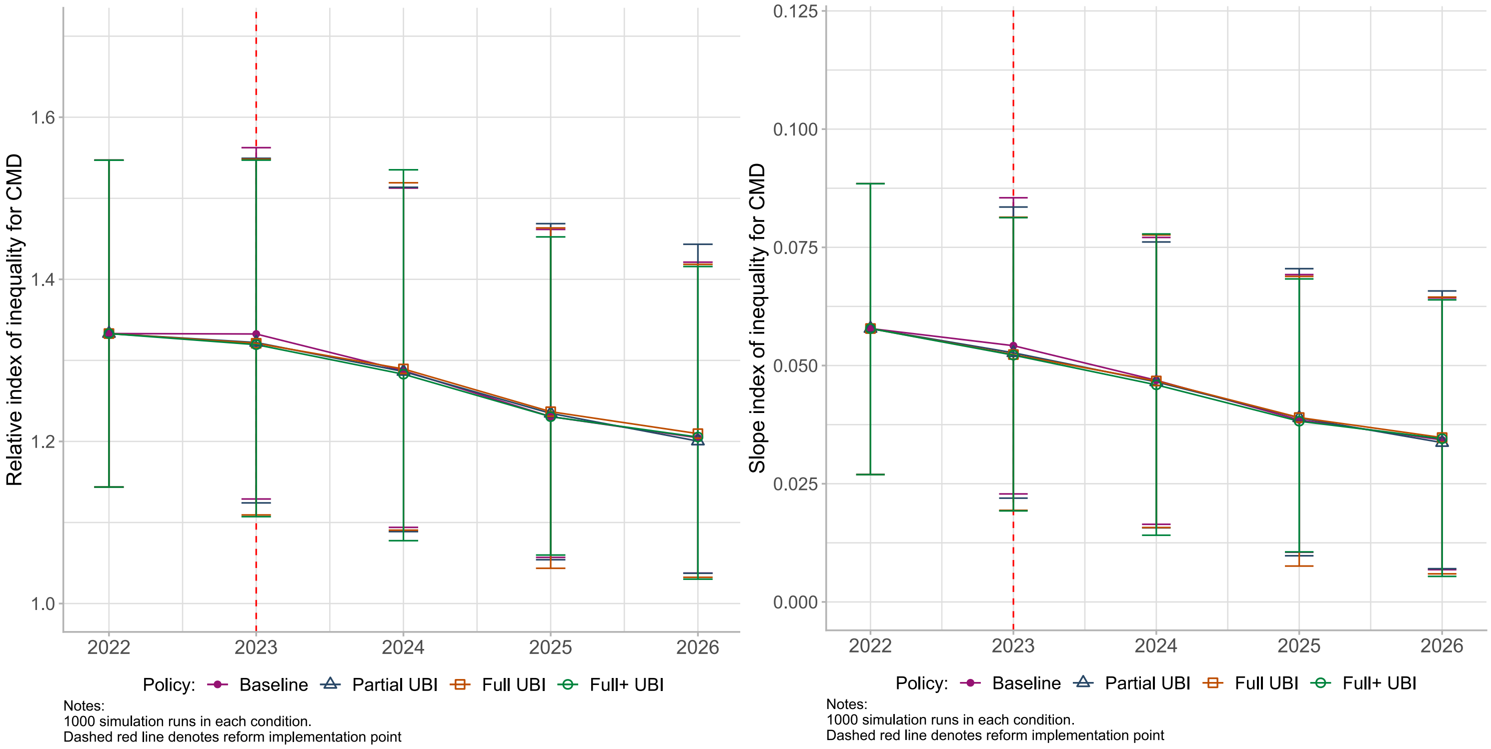

Baseline = planned tax/benefit policies for United Kingdom. Partial UBI = UBI set at level of existing benefits. Full UBI = UBI set at level of Minimum Income Standard. Full+ UBI = Minimum Income Standard plus means-tested benefits for caring, childcare, disability, housing, limited capability for work. Whiskers = 95% Uncertainty intervals.

Figure M: Structural Sensitivity Analysis 1, relaxing employment assumptions – Estimated prevalence of common mental disorder (CMD) for modelled Universal Basic Income (UBI) policies from 2022 to 2026 with 95% uncertainty intervals, stratified by gender (A), education (B), age (C), and household structure (D). Note different scales used for each stratification.

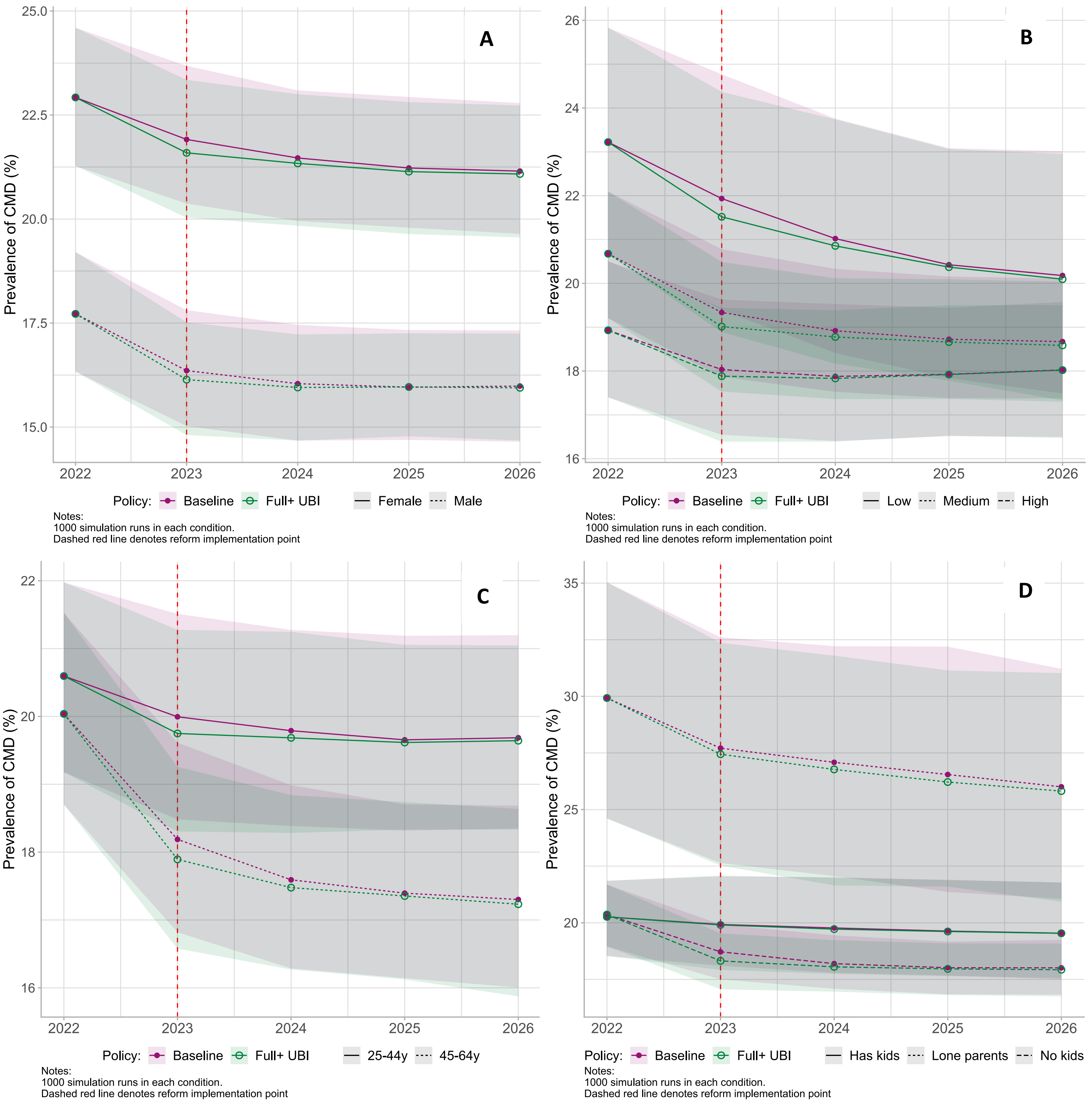

y = years. Baseline = planned tax/benefit policies for United Kingdom. Full+ UBI = UBI set at level of Minimum Income Standard plus means-tested benefits for caring, childcare, disability, housing, limited capability for work. Ribbons = 95% Uncertainty intervals. Low education = no formal qualifications; medium education = Higher/A-level/GCSE [General Certificate of Secondary Education] or equivalent; high education = degree or equivalent.

Figure N: Structural Sensitivity Analysis 2, using economic inactivity effects – Estimated prevalence of common mental disorder (CMD) for modelled Universal Basic Income (UBI) policies from 2022 to 2026 stratified by gender (A), education (B), age (C), and household structure (D). Note different scales used for each stratification.

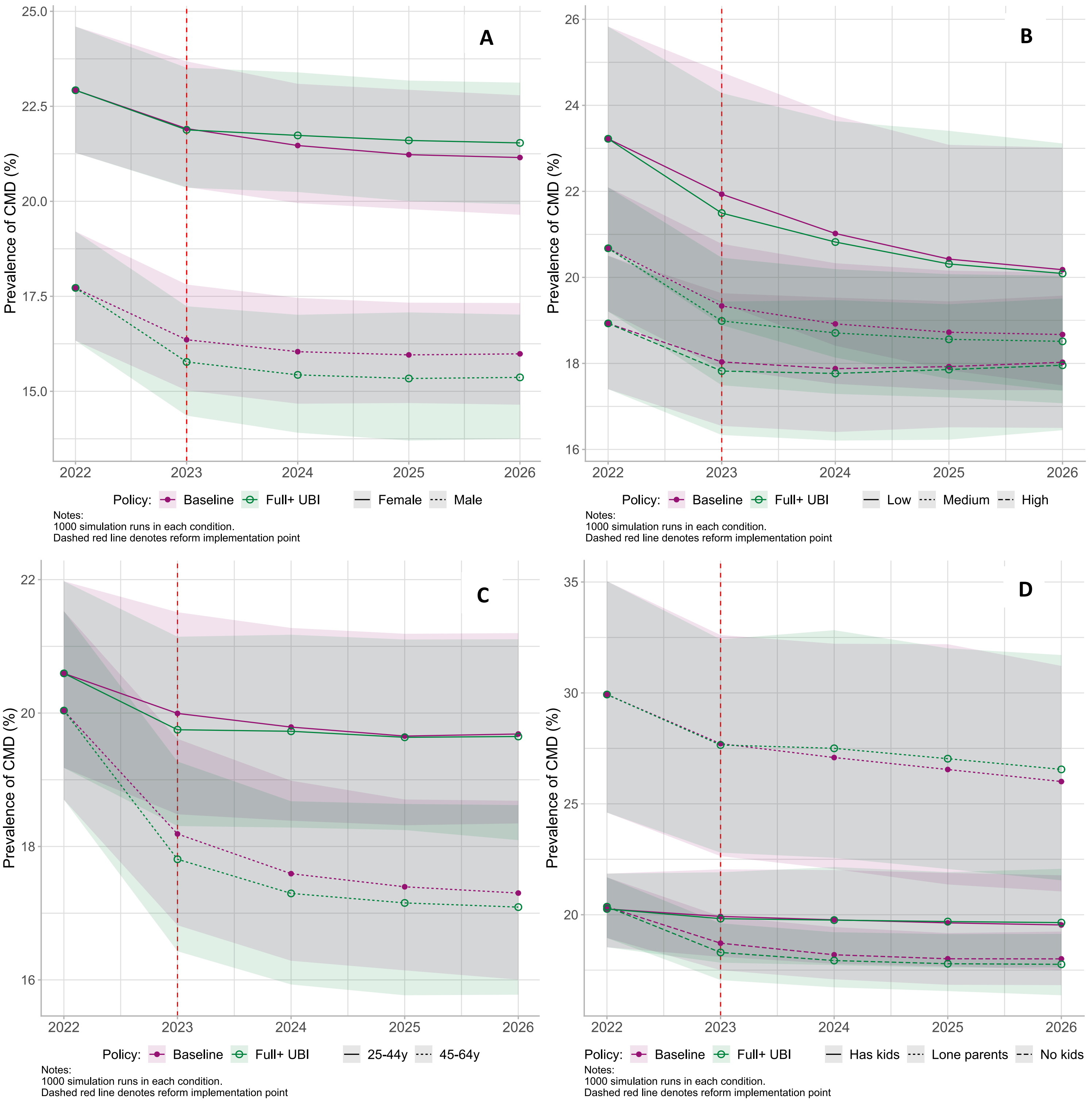

y = years. Baseline = planned tax/benefit policies for United Kingdom. Full+ UBI = UBI set at level of Minimum Income Standard plus means-tested benefits for caring, childcare, disability, housing, limited capability for work. Ribbons = 95% Uncertainty intervals. Low education = no formal qualifications; medium education = Higher/A-level/GCSE [General Certificate of Secondary Education] or equivalent; high education = degree or equivalent.

Table K: Structural Sensitivity Analyses – Estimated prevalence of common mental disorders in baseline scenario and three simulated Universal Basic Income (UBI) scenarios from 2022-2026 stratified by gender, education, age, previous poverty/employment status, and household structure (95% uncertainty intervals)

Subgroup	Scenario		2022	2023	2024	2025	2026
**STRUCTURAL SENSITIVITY ANALYSIS 1: RELAXING EMPLOYMENT ASSUMPTIONS**							
**Men**	Baseline		17.73 (16.33, 19.21)	16.35 (15.02, 17.81)	16.03 (14.67, 17.46)	15.92 (14.69, 17.33)	15.96 (14.65, 17.32)
	Partial		17.73 (16.33, 19.21)	16.28 (14.95, 17.68)	15.99 (14.69, 17.38)	15.97 (14.81, 17.50)	16.04 (14.68, 17.40)
		Difference:		-0.06 (-0.18, 0.05)	-0.03 (-1.19, 1.12)	0.05 (-1.13, 1.25)	0.05 (-1.11, 1.17)
	Full		17.73 (16.33, 19.21)	16.19 (14.86, 17.63)	15.96 (14.72, 17.38)	15.95 (14.72, 17.25)	15.98 (14.71, 17.28)
		Difference:		-0.17 (-0.46, 0.11)	-0.05 (-1.27, 1.09)	0.05 (-1.20, 1.12)	-0.02 (-1.22, 1.17)
	Full+		17.73 (16.33, 19.21)	16.15 (14.81, 17.52)	15.94 (14.67, 17.23)	15.93 (14.78, 17.25)	15.92 (14.68, 17.25)
		Difference:		-0.22 (-0.51, 0.06)	-0.09 (-1.22, 1.09)	0.00 (-1.19, 1.17)	-0.05 (-1.20, 1.06)
	n		6446 (6446, 6446)	6503 (6502, 6504)	6542 (6542, 6543)	6583 (6582, 6584)	6581 (6581, 6581)
**Women**	Baseline		22.93 (21.28, 24.60)	21.90 (20.37, 23.68)	21.44 (19.95, 23.09)	21.20 (19.79, 22.93)	21.12 (19.64, 22.79)
	Partial		22.93 (21.28, 24.60)	21.76 (20.26, 23.48)	21.44 (19.85, 23.08)	21.20 (19.64, 22.84)	21.13 (19.63, 22.82)
		Difference:		-0.14 (-0.38, 0.08)	-0.02 (-1.25, 1.14)	-0.02 (-1.30, 1.34)	0.02 (-1.32, 1.28)
	Full		22.93 (21.28, 24.60)	21.59 (20.08, 23.38)	21.28 (19.75, 22.97)	21.12 (19.72, 22.83)	21.02 (19.55, 22.70)
		Difference:		-0.29 (-0.65, 0.02)	-0.15 (-1.35, 1.08)	-0.02 (-1.40, 1.24)	-0.11 (-1.44, 1.27)
	Full+		22.93 (21.28, 24.60)	21.56 (20.03, 23.35)	21.34 (19.84, 23.00)	21.12 (19.64, 22.81)	21.05 (19.56, 22.73)
		Difference:		-0.32 (-0.65, 0.00)	-0.12 (-1.39, 1.11)	-0.09 (-1.37, 1.10)	-0.06 (-1.36, 1.13)
	n		6472 (6472, 6473)	6494 (6494, 6494)	6502 (6500, 6503)	6558 (6558, 6559)	6539 (6538, 6541)
**Low education**	Baseline		23.23 (20.66, 25.83)	21.91 (19.39, 24.77)	21.09 (18.41, 23.76)	20.39 (17.84, 23.08)	20.23 (17.49, 23.02)
	Partial		23.23 (20.66, 25.83)	21.77 (19.20, 24.63)	20.90 (18.44, 23.52)	20.38 (17.83, 23.37)	20.27 (17.48, 23.32)
		Difference:		-0.15 (-0.58, 0.28)	-0.08 (-2.75, 2.89)	0.05 (-2.90, 2.96)	0.18 (-3.21, 3.42)
	Full		23.23 (20.66, 25.83)	21.47 (18.85, 24.37)	20.89 (18.15, 23.73)	20.37 (17.63, 23.01)	20.07 (17.39, 23.06)
		Difference:		-0.43 (-0.98, 0.08)	-0.11 (-3.08, 2.93)	-0.02 (-3.20, 3.05)	-0.10 (-3.29, 3.48)
	Full+		23.23 (20.66, 25.83)	21.50 (18.89, 24.37)	20.85 (18.17, 23.74)	20.32 (17.78, 23.06)	20.07 (17.33, 22.95)

		Difference:	-0.42 (-0.97, 0.15)	-0.24 (-2.97, 2.66)	-0.01 (-3.19, 2.70)	-0.02 (-3.31, 3.31)
	n	1424 (1342, 1509)	1360 (1278, 1445)	1288 (1205, 1376)	1221 (1139, 1308)	1129 (1046, 1218)
**Medium education**	Baseline	20.69 (19.21, 22.09)	19.34 (17.87, 20.78)	18.91 (17.53, 20.33)	18.72 (17.38, 20.16)	18.66 (17.38, 20.11)
	Partial	20.69 (19.21, 22.09)	19.22 (17.74, 20.68)	18.87 (17.62, 20.26)	18.76 (17.44, 20.16)	18.69 (17.46, 20.07)
		Difference:	-0.12 (-0.30, 0.06)	-0.04 (-1.17, 1.15)	0.06 (-1.13, 1.22)	0.03 (-1.14, 1.18)
	Full	20.71 (19.11, 22.19)	19.05 (17.49, 20.71)	18.74 (17.28, 20.27)	18.68 (17.53, 19.91)	18.58 (17.44, 20.02)
		Difference:	-0.27 (-0.57, -0.03)	-0.21 (-1.45, 1.03)	-0.06 (-1.35, 1.13)	-0.07 (-1.27, 1.29)
	Full+	20.71 (19.11, 22.19)	19.04 (17.48, 20.69)	18.77 (17.29, 20.12)	18.62 (17.37, 20.13)	18.57 (17.46, 19.99)
		Difference:	-0.32 (-0.58, -0.08)	-0.19 (-1.29, 1.02)	-0.09 (-1.50, 1.03)	-0.11 (-1.27, 1.09)
	n	6845 (6702, 6992)	6914 (6764, 7053)	7001 (6859, 7144)	7086 (6932, 7224)	7115 (6968, 7262)
**High education**	Baseline	18.94 (17.40, 20.50)	17.99 (16.55, 19.63)	17.85 (16.40, 19.53)	17.89 (16.51, 19.44)	18.02 (16.50, 19.58)
	Partial	18.94 (17.40, 20.50)	17.95 (16.48, 19.56)	17.84 (16.32, 19.54)	17.93 (16.34, 19.46)	18.06 (16.59, 19.60)
		Difference:	-0.06 (-0.30, 0.15)	-0.02 (-1.46, 1.39)	-0.02 (-1.50, 1.50)	0.01 (-1.37, 1.47)
	Full	18.94 (17.40, 20.50)	17.92 (16.44, 19.56)	17.80 (16.30, 19.47)	17.94 (16.46, 19.53)	17.99 (16.44, 19.46)
		Difference:	-0.11 (-0.39, 0.19)	-0.08 (-1.44, 1.17)	-0.01 (-1.49, 1.48)	-0.03 (-1.55, 1.50)
	Full+	18.94 (17.40, 20.50)	17.87 (16.39, 19.44)	17.84 (16.39, 19.38)	17.87 (16.53, 19.50)	18.02 (16.47, 19.50)
		Difference:	-0.15 (-0.44, 0.17)	-0.02 (-1.36, 1.32)	0.01 (-1.57, 1.41)	-0.00 (-1.44, 1.46)
	n	4653 (4518, 4798)	4733 (4596, 4876)	4758 (4618, 4903)	4846 (4700, 4993)	4884 (4735, 5039)
**Age group: 25-44 years**	Baseline	20.59 (19.18, 21.98)	19.98 (18.48, 21.51)	19.76 (18.39, 21.28)	19.63 (18.32, 21.19)	19.69 (18.35, 21.20)
	Partial	20.59 (19.18, 21.98)	19.88 (18.45, 21.34)	19.79 (18.42, 21.27)	19.69 (18.43, 21.13)	19.70 (18.37, 21.13)
		Difference:	-0.09 (-0.26, 0.07)	0.00 (-1.12, 1.14)	0.06 (-1.14, 1.21)	0.07 (-1.18, 1.20)
	Full	20.59 (19.18, 21.98)	19.84 (18.38, 21.37)	19.70 (18.32, 21.29)	19.62 (18.27, 21.09)	19.66 (18.29, 21.18)
		Difference:	-0.13 (-0.41, 0.15)	-0.07 (-1.24, 1.07)	-0.01 (-1.30, 1.14)	-0.01 (-1.27, 1.31)
	Full+	20.59 (19.18, 21.98)	19.73 (18.31, 21.28)	19.66 (18.28, 21.25)	19.57 (18.33, 21.06)	19.62 (18.33, 21.05)
		Difference:	-0.25 (-0.53, 0.04)	-0.11 (-1.30, 1.09)	-0.03 (-1.34, 1.08)	-0.03 (-1.33, 1.13)
	n	6721 (6721, 6721)	6801 (6801, 6801)	6855 (6853, 6856)	6933 (6933, 6935)	6928 (6927, 6930)

**Age group: 45-64 years**	Baseline	20.02 (18.70, 21.53)	18.17 (16.82, 19.61)	17.56 (16.29, 18.99)	17.41 (16.14, 18.70)	17.30 (16.00, 18.69)
	Partial	20.02 (18.70, 21.53)	18.06 (16.75, 19.47)	17.51 (16.22, 18.90)	17.40 (16.06, 18.65)	17.32 (16.10, 18.59)
		Difference:	-0.11 (-0.32, 0.08)	-0.06 (-1.23, 1.16)	0.00 (-1.29, 1.32)	0.02 (-1.16, 1.24)
	Full	20.02 (18.70, 21.53)	17.85 (16.51, 19.24)	17.42 (16.24, 18.86)	17.34 (16.05, 18.67)	17.18 (15.99, 18.52)
		Difference:	-0.34 (-0.63, -0.03)	-0.13 (-1.37, 1.05)	-0.05 (-1.34, 1.22)	-0.11 (-1.31, 1.13)
	Full+	20.02 (18.70, 21.53)	17.89 (16.58, 19.26)	17.47 (16.27, 18.84)	17.35 (16.12, 18.74)	17.25 (15.87, 18.64)
		Difference:	-0.29 (-0.60, -0.02)	-0.13 (-1.36, 1.10)	-0.05 (-1.34, 1.21)	-0.07 (-1.34, 1.23)
	n	6197 (6197, 6198)	6196 (6195, 6197)	6189 (6189, 6190)	6208 (6207, 6208)	6192 (6192, 6192)
**Have children**	Baseline	20.28 (18.53, 21.85)	19.87 (18.10, 22.05)	19.71 (17.79, 22.02)	19.62 (17.67, 21.88)	19.55 (17.50, 21.78)
	Partial	20.28 (18.53, 21.85)	19.81 (17.98, 22.00)	19.71 (17.73, 22.03)	19.61 (17.59, 21.89)	19.53 (17.49, 21.90)
		Difference:	-0.07 (-0.32, 0.18)	-0.03 (-1.37, 1.43)	-0.00 (-1.61, 1.54)	0.04 (-1.45, 1.51)
	Full	20.28 (18.53, 21.85)	19.90 (18.01, 22.21)	19.67 (17.70, 22.21)	19.60 (17.65, 21.92)	19.50 (17.59, 21.77)
		Difference:	0.02 (-0.30, 0.40)	-0.07 (-1.56, 1.43)	0.02 (-1.52, 1.63)	0.04 (-1.53, 1.48)
	Full+	20.28 (18.53, 21.85)	19.84 (17.92, 22.09)	19.67 (17.74, 21.98)	19.60 (17.64, 21.90)	19.49 (17.56, 21.78)
		Difference:	-0.02 (-0.36, 0.31)	-0.04 (-1.51, 1.42)	-0.04 (-1.55, 1.53)	-0.01 (-1.55, 1.46)
	n	4464 (4352, 4573)	4482 (4368, 4589)	4564 (4466, 4677)	4648 (4552, 4753)	4719 (4617, 4831)
**No children**	Baseline	20.36 (18.95, 21.69)	18.71 (17.50, 19.93)	18.19 (17.09, 19.44)	18.03 (16.84, 19.17)	18.00 (16.83, 19.24)
	Partial	20.36 (18.95, 21.69)	18.60 (17.38, 19.78)	18.15 (17.08, 19.39)	18.04 (16.92, 19.26)	18.03 (16.84, 19.21)
		Difference:	-0.13 (-0.28, 0.02)	-0.01 (-1.06, 1.01)	0.03 (-1.05, 1.07)	0.04 (-1.00, 1.02)
	Full	20.36 (18.95, 21.69)	18.33 (17.10, 19.56)	18.03 (16.94, 19.25)	17.95 (16.81, 19.13)	17.93 (16.83, 19.10)
		Difference:	-0.37 (-0.65, -0.13)	-0.14 (-1.14, 0.83)	-0.03 (-1.08, 0.93)	-0.08 (-1.23, 1.03)
	Full+	20.36 (18.95, 21.69)	18.31 (17.06, 19.54)	18.04 (16.96, 19.23)	17.95 (16.81, 19.09)	17.92 (16.75, 19.08)
		Difference:	-0.40 (-0.68, -0.15)	-0.11 (-1.14, 0.81)	-0.04 (-1.09, 0.94)	-0.09 (-1.22, 1.01)
	n	8454 (8345, 8566)	8515 (8407, 8628)	8480 (8368, 8577)	8493 (8389, 8588)	8400 (8288, 8502)
**Lone parent**	Baseline	29.83 (24.61, 35.03)	27.73 (22.64, 32.61)	27.08 (22.06, 32.22)	26.50 (21.36, 32.20)	25.95 (21.05, 31.22)
	Partial	29.83 (24.61, 35.03)	27.44 (22.39, 32.21)	27.03 (22.17, 32.39)	26.63 (21.93, 32.02)	26.14 (21.09, 31.45)

		Difference:	-0.24 (-1.27, 0.98)	-0.08 (-5.53, 5.27)	0.07 (-6.18, 6.32)	0.17 (-5.74, 6.12)
	Full	29.83 (24.61, 35.03)	27.43 (22.41, 32.17)	26.67 (22.11, 31.62)	26.29 (21.46, 31.67)	25.95 (20.99, 31.07)
		Difference:	-0.26 (-1.49, 0.72)	-0.42 (-5.88, 5.65)	-0.26 (-6.48, 6.29)	-0.15 (-6.09, 5.63)
	Full+	29.83 (24.61, 35.03)	27.56 (22.52, 32.36)	26.81 (21.65, 31.80)	26.14 (21.59, 31.14)	25.81 (20.94, 31.02)
		Difference:	-0.25 (-1.44, 0.74)	-0.22 (-6.21, 5.08)	-0.36 (-6.53, 5.70)	-0.16 (-6.36, 5.82)
	n	435 (391, 480)	411 (368, 461)	406 (361, 454)	392 (346, 441)	381 (334, 432)
**STRUCTURAL SENSITIVITY ANALYSIS 2: USING ECONOMIC INACTIVITY EFFECTS IN FULL UBI YEARS**						
**Men**	Baseline	17.73 (16.33, 19.21)	16.35 (15.02, 17.81)	16.03 (14.67, 17.46)	15.92 (14.69, 17.33)	15.96 (14.65, 17.32)
	Partial	17.73 (16.33, 19.21)	16.28 (14.95, 17.68)	15.99 (14.69, 17.38)	15.97 (14.81, 17.50)	16.04 (14.68, 17.40)
		Difference:	-0.06 (-0.18, 0.05)	-0.03 (-1.19, 1.12)	0.05 (-1.13, 1.25)	0.05 (-1.11, 1.17)
	Full	17.73 (16.33, 19.21)	15.76 (14.37, 17.20)	15.39 (13.98, 17.03)	15.28 (13.83, 16.99)	15.29 (13.87, 17.00)
		Difference:	-0.62 (-1.54, 0.29)	-0.67 (-2.09, 0.90)	-0.66 (-2.14, 1.05)	-0.67 (-2.22, 1.06)
	Full+	17.73 (16.33, 19.21)	15.79 (14.36, 17.24)	15.41 (13.91, 17.01)	15.31 (13.70, 17.08)	15.32 (13.75, 17.02)
		Difference:	-0.58 (-1.52, 0.37)	-0.61 (-2.16, 0.93)	-0.64 (-2.22, 1.08)	-0.61 (-2.32, 1.06)
	n	6446 (6446, 6446)	6503 (6502, 6504)	6542 (6542, 6543)	6583 (6582, 6584)	6581 (6581, 6581)
**Women**	Baseline	22.93 (21.28, 24.60)	21.90 (20.37, 23.68)	21.44 (19.95, 23.09)	21.20 (19.79, 22.93)	21.12 (19.64, 22.79)
	Partial	22.93 (21.28, 24.60)	21.76 (20.26, 23.48)	21.44 (19.85, 23.08)	21.20 (19.64, 22.84)	21.13 (19.63, 22.82)
		Difference:	-0.14 (-0.38, 0.08)	-0.02 (-1.25, 1.14)	-0.02 (-1.30, 1.34)	0.02 (-1.32, 1.28)
	Full	22.93 (21.28, 24.60)	21.84 (20.34, 23.44)	21.71 (20.03, 23.33)	21.57 (19.91, 23.21)	21.49 (19.94, 23.16)
		Difference:	-0.05 (-0.99, 0.77)	0.26 (-1.34, 1.77)	0.37 (-1.45, 1.88)	0.40 (-1.32, 2.08)
	Full+	22.93 (21.28, 24.60)	21.85 (20.36, 23.51)	21.69 (20.25, 23.39)	21.58 (20.01, 23.18)	21.54 (19.92, 23.12)
		Difference:	-0.03 (-0.97, 0.82)	0.25 (-1.17, 1.78)	0.38 (-1.37, 1.97)	0.38 (-1.38, 2.02)
	n	6472 (6472, 6473)	6494 (6494, 6494)	6502 (6500, 6503)	6558 (6558, 6559)	6539 (6538, 6541)
**Low education**	Baseline	23.23 (20.66, 25.83)	21.91 (19.39, 24.77)	21.09 (18.41, 23.76)	20.39 (17.84, 23.08)	20.23 (17.49, 23.02)
	Partial	23.23 (20.66, 25.83)	21.77 (19.20, 24.63)	20.90 (18.44, 23.52)	20.38 (17.83, 23.37)	20.27 (17.48, 23.32)
		Difference:	-0.15 (-0.58, 0.28)	-0.08 (-2.75, 2.89)	0.05 (-2.90, 2.96)	0.18 (-3.21, 3.42)
	Full	23.23 (20.66, 25.83)	21.49 (18.85, 24.23)	20.74 (18.13, 23.59)	20.30 (17.60, 23.22)	20.08 (17.18, 22.90)

		Difference:	-0.46 (-1.61, 0.62)	-0.30 (-3.25, 2.84)	-0.08 (-3.24, 3.03)	-0.03 (-3.33, 3.04)
	Full+	23.23 (20.66, 25.83)	21.53 (18.89, 24.29)	20.77 (18.13, 23.64)	20.24 (17.64, 23.41)	20.09 (17.36, 23.11)
		Difference:	-0.43 (-1.59, 0.68)	-0.24 (-3.19, 2.89)	-0.17 (-3.35, 3.08)	-0.08 (-3.57, 3.18)
	n	1424 (1342, 1509)	1360 (1278, 1445)	1288 (1205, 1376)	1220 (1139, 1307)	1130 (1048, 1215)
**Medium education**	Baseline	20.69 (19.21, 22.09)	19.34 (17.87, 20.78)	18.91 (17.53, 20.33)	18.72 (17.38, 20.16)	18.66 (17.38, 20.11)
	Partial	20.69 (19.21, 22.09)	19.22 (17.74, 20.68)	18.87 (17.62, 20.26)	18.76 (17.44, 20.16)	18.69 (17.46, 20.07)
		Difference:	-0.12 (-0.30, 0.06)	-0.04 (-1.17, 1.15)	0.06 (-1.13, 1.22)	0.03 (-1.14, 1.18)
	Full	20.69 (19.21, 22.09)	18.97 (17.50, 20.41)	18.65 (17.39, 20.10)	18.51 (17.20, 19.97)	18.46 (17.13, 19.89)
		Difference:	-0.38 (-1.06, 0.31)	-0.24 (-1.64, 1.23)	-0.18 (-1.57, 1.20)	-0.21 (-1.62, 1.39)
	Full+	20.69 (19.21, 22.09)	19.00 (17.49, 20.46)	18.68 (17.29, 20.19)	18.55 (17.21, 20.07)	18.49 (17.07, 20.04)
		Difference:	-0.35 (-1.04, 0.35)	-0.21 (-1.59, 1.27)	-0.20 (-1.66, 1.38)	-0.17 (-1.54, 1.32)
	n	6841 (6701, 6991)	6906 (6763, 7052)	6998 (6853, 7143)	7075 (6924, 7224)	7107 (6949, 7254)
**High education**	Baseline	18.94 (17.40, 20.50)	17.99 (16.55, 19.63)	17.85 (16.40, 19.53)	17.89 (16.51, 19.44)	18.02 (16.50, 19.58)
	Partial	18.94 (17.40, 20.50)	17.95 (16.48, 19.56)	17.84 (16.32, 19.54)	17.93 (16.34, 19.46)	18.06 (16.59, 19.60)
		Difference:	-0.06 (-0.30, 0.15)	-0.02 (-1.46, 1.39)	-0.02 (-1.50, 1.50)	0.01 (-1.37, 1.47)
	Full	18.94 (17.40, 20.50)	17.81 (16.30, 19.42)	17.77 (16.16, 19.36)	17.83 (16.23, 19.42)	17.92 (16.32, 19.67)
		Difference:	-0.25 (-0.96, 0.53)	-0.15 (-1.71, 1.24)	-0.08 (-1.72, 1.40)	-0.08 (-1.72, 1.67)
	Full+	18.94 (17.40, 20.50)	17.83 (16.34, 19.44)	17.75 (16.21, 19.47)	17.87 (16.23, 19.38)	17.95 (16.45, 19.51)
		Difference:	-0.23 (-0.93, 0.56)	-0.06 (-1.68, 1.29)	-0.06 (-1.67, 1.51)	-0.08 (-1.78, 1.63)
	n	4653 (4518, 4798)	4733 (4596, 4876)	4758 (4618, 4903)	4847 (4704, 4991)	4884 (4737, 5035)
**Age group: 25-44 years**	Baseline	20.59 (19.18, 21.98)	19.98 (18.48, 21.51)	19.76 (18.39, 21.28)	19.63 (18.32, 21.19)	19.69 (18.35, 21.20)
	Partial	20.59 (19.18, 21.98)	19.88 (18.45, 21.34)	19.79 (18.42, 21.27)	19.69 (18.43, 21.13)	19.70 (18.37, 21.13)
		Difference:	-0.09 (-0.26, 0.07)	0.00 (-1.12, 1.14)	0.06 (-1.14, 1.21)	0.07 (-1.18, 1.20)
	Full	20.59 (19.18, 21.98)	19.70 (18.28, 21.16)	19.66 (18.19, 21.21)	19.60 (18.15, 21.20)	19.65 (18.18, 21.08)
		Difference:	-0.26 (-0.94, 0.38)	-0.12 (-1.37, 1.31)	-0.03 (-1.44, 1.36)	-0.04 (-1.55, 1.49)
	Full+	20.59 (19.18, 21.98)	19.73 (18.31, 21.14)	19.73 (18.28, 21.17)	19.62 (18.25, 21.10)	19.66 (18.10, 21.11)

		Difference:	-0.25 (-0.91, 0.41)	-0.04 (-1.36, 1.24)	-0.01 (-1.44, 1.31)	-0.04 (-1.49, 1.39)
	n	6721 (6721, 6721)	6801 (6801, 6801)	6855 (6853, 6856)	6933 (6933, 6935)	6928 (6927, 6930)
**Age group: 45-64 years**	Baseline	20.02 (18.70, 21.53)	18.17 (16.82, 19.61)	17.56 (16.29, 18.99)	17.41 (16.14, 18.70)	17.30 (16.00, 18.69)
	Partial	20.02 (18.70, 21.53)	18.06 (16.75, 19.47)	17.51 (16.22, 18.90)	17.40 (16.06, 18.65)	17.32 (16.10, 18.59)
		Difference:	-0.11 (-0.32, 0.08)	-0.06 (-1.23, 1.16)	0.00 (-1.29, 1.32)	0.02 (-1.16, 1.24)
	Full	20.02 (18.70, 21.53)	17.76 (16.43, 19.19)	17.26 (15.90, 18.74)	17.12 (15.74, 18.51)	17.07 (15.67, 18.49)
		Difference:	-0.44 (-1.15, 0.32)	-0.32 (-1.73, 1.15)	-0.29 (-1.72, 1.18)	-0.27 (-1.74, 1.29)
	Full+	20.02 (18.70, 21.53)	17.80 (16.43, 19.27)	17.26 (15.93, 18.68)	17.13 (15.77, 18.64)	17.04 (15.78, 18.62)
		Difference:	-0.39 (-1.10, 0.37)	-0.29 (-1.78, 1.08)	-0.27 (-1.66, 1.27)	-0.21 (-1.66, 1.38)
	n	6197 (6197, 6198)	6196 (6195, 6197)	6189 (6189, 6190)	6208 (6207, 6208)	6192 (6192, 6192)
**Have children**	Baseline	20.28 (18.53, 21.85)	19.87 (18.10, 22.05)	19.71 (17.79, 22.02)	19.62 (17.67, 21.88)	19.55 (17.50, 21.78)
	Partial	20.28 (18.53, 21.85)	19.81 (17.98, 22.00)	19.71 (17.73, 22.03)	19.61 (17.59, 21.89)	19.53 (17.49, 21.90)
		Difference:	-0.07 (-0.32, 0.18)	-0.03 (-1.37, 1.43)	-0.00 (-1.61, 1.54)	0.04 (-1.45, 1.51)
	Full	20.28 (18.53, 21.85)	19.77 (17.87, 21.84)	19.72 (17.51, 21.95)	19.64 (17.49, 22.04)	19.57 (17.50, 21.91)
		Difference:	-0.11 (-0.87, 0.62)	-0.00 (-1.64, 1.57)	0.05 (-1.71, 1.82)	0.07 (-1.65, 1.82)
	Full+	20.28 (18.53, 21.85)	19.81 (17.84, 21.91)	19.72 (17.73, 22.15)	19.68 (17.62, 21.92)	19.61 (17.63, 22.07)
		Difference:	-0.09 (-0.82, 0.69)	-0.00 (-1.54, 1.57)	0.05 (-1.75, 1.83)	0.12 (-1.63, 1.82)
	n	4464 (4352, 4573)	4482 (4368, 4589)	4566 (4466, 4671)	4649 (4547, 4752)	4720 (4615, 4824)
**No children**	Baseline	20.36 (18.95, 21.69)	18.71 (17.50, 19.93)	18.19 (17.09, 19.44)	18.03 (16.84, 19.17)	18.00 (16.83, 19.24)
	Partial	20.36 (18.95, 21.69)	18.60 (17.38, 19.78)	18.15 (17.08, 19.39)	18.04 (16.92, 19.26)	18.03 (16.84, 19.21)
		Difference:	-0.13 (-0.28, 0.02)	-0.01 (-1.06, 1.01)	0.03 (-1.05, 1.07)	0.04 (-1.00, 1.02)
	Full	20.36 (18.95, 21.69)	18.28 (17.05, 19.56)	17.87 (16.64, 19.17)	17.75 (16.62, 18.96)	17.76 (16.55, 18.98)
		Difference:	-0.46 (-1.13, 0.26)	-0.27 (-1.58, 0.94)	-0.26 (-1.42, 0.89)	-0.24 (-1.59, 1.22)
	Full+	20.36 (18.95, 21.69)	18.30 (17.06, 19.61)	17.91 (16.73, 19.21)	17.76 (16.56, 19.13)	17.76 (16.37, 19.14)
		Difference:	-0.42 (-1.11, 0.29)	-0.26 (-1.55, 1.01)	-0.26 (-1.45, 1.09)	-0.27 (-1.65, 1.15)
	n	8454 (8345, 8566)	8515 (8407, 8628)	8478 (8372, 8578)	8491 (8390, 8593)	8400 (8295, 8504)

**Lone parent**	Baseline	29.83 (24.61, 35.03)	27.73 (22.64, 32.61)	27.08 (22.06, 32.22)	26.50 (21.36, 32.20)	25.95 (21.05, 31.22)
	Partial	29.83 (24.61, 35.03)	27.44 (22.39, 32.21)	27.03 (22.17, 32.39)	26.63 (21.93, 32.02)	26.14 (21.09, 31.45)
		Difference:	-0.24 (-1.27, 0.98)	-0.08 (-5.53, 5.27)	0.07 (-6.18, 6.32)	0.17 (-5.74, 6.12)
	Full	29.83 (24.61, 35.03)	27.71 (22.68, 32.33)	27.27 (22.39, 32.87)	26.82 (21.89, 32.16)	26.40 (21.66, 32.02)
		Difference:	0.00 (-2.24, 1.93)	0.38 (-5.40, 6.38)	0.43 (-6.19, 6.58)	0.53 (-5.55, 6.80)
	Full+	29.83 (24.61, 35.03)	27.76 (22.80, 32.41)	27.54 (22.56, 32.83)	27.02 (22.06, 32.01)	26.56 (21.55, 31.71)
		Difference:	0.00 (-2.19, 2.04)	0.45 (-5.55, 6.20)	0.48 (-5.78, 6.75)	0.57 (-5.87, 6.89)
	n	435 (391, 480)	411 (368, 461)	404 (359, 455)	392 (348, 445)	381 (333, 437)

Baseline = planned tax/benefit policies for United Kingdom. Partial UBI = UBI set at level of existing benefits. Full UBI = UBI set at level of Minimum Income Standard. Full+ UBI = Minimum Income Standard plus means-tested benefits for caring, childcare, disability, housing, limited capability for work. Low education = no formal qualifications; medium education = Higher/A-level/GCSE [General Certificate of Secondary Education] or equivalent; high education = degree or equivalent.

Results from analytical sensitivity analysis

Table L: Analytical Sensitivity Analyses – Median income, prevalence of poverty, and prevalence of unemployment in baseline scenario and three simulated Universal Basic Income (UBI) scenarios from 2022-2026 (95% uncertainty intervals)

Variable	Scenario	2022	2023	2024	2025	2026
**Median annual income (£)**	Baseline	21345.25 (20927.66, 21736.35)	22577.72 (22110.97, 23014.91)	23211.57 (22779.28, 23754.39)	24057.68 (23530.69, 24644.04)	25017.80 (24488.96, 25533.61)
	Partial	21345.25 (20927.66, 21736.35)	27172.90 (26599.29, 27652.72)	27992.75 (27536.77, 28513.33)	28936.35 (28455.66, 29497.71)	30028.81 (29445.47, 30586.48)
	Full	21345.25 (20927.66, 21736.35)	27420.63 (27190.69, 27629.56)	27980.31 (27763.91, 28219.95)	28668.42 (28440.86, 28924.84)	29409.85 (29142.28, 29642.18)
	Full+	21345.25 (20927.66, 21736.35)	27719.13 (27474.30, 27922.25)	28289.46 (28059.58, 28488.10)	28947.66 (28753.55, 29145.77)	29675.30 (29472.07, 29904.67)
**Poverty (%)**	Baseline	11.72 (11.09, 12.41)	9.10 (8.47, 9.71)	9.30 (8.70, 9.93)	9.38 (8.86, 10.05)	9.60 (8.97, 10.23)
	Partial	11.72 (11.09, 12.41)	7.13 (6.62, 7.66)	6.86 (6.32, 7.44)	6.62 (6.07, 7.19)	6.52 (5.99, 7.10)
	Full	11.72 (11.09, 12.41)	0.02 (0.00, 0.05)	0.02 (0.00, 0.05)	0.03 (0.01, 0.07)	0.04 (0.01, 0.08)
	Full+	11.72 (11.09, 12.41)	0.01 (0.00, 0.03)	0.02 (0.00, 0.04)	0.02 (0.00, 0.05)	0.02 (0.00, 0.05)
**Employ-ment (%)**	Baseline	77.09 (76.13, 77.99)	78.93 (77.94, 79.86)	79.93 (78.96, 80.83)	80.78 (79.79, 81.65)	81.42 (80.48, 82.25)
	Partial	77.09 (76.13, 77.99)	79.38 (78.41, 80.24)	80.32 (79.32, 81.19)	81.01 (80.03, 81.91)	81.44 (80.44, 82.30)
	Full	77.09 (76.13, 77.99)	75.69 (74.44, 76.92)	76.47 (75.14, 77.79)	77.08 (75.76, 78.21)	77.42 (76.12, 78.69)
	Full+	77.09 (76.13, 77.99)	74.10 (72.62, 75.43)	74.84 (73.30, 76.21)	75.43 (73.86, 76.86)	75.81 (74.27, 77.26)
	n	12918 (12918, 12919)	12997 (12996, 12998)	13044 (13042, 13046)	13141 (13140, 13143)	13120 (13119, 13122)

Baseline = planned tax/benefit policies for United Kingdom. Partial UBI = UBI set at level of existing benefits. Full UBI = UBI set at level of Minimum Income Standard. Full+ UBI = Minimum Income Standard plus means-tested benefits for caring, childcare, disability, housing, limited capability for work.

Figure O: Analytical Sensitivity Analysis, using alternative estimates from systematic reviews - Estimated prevalence of common mental disorder (CMD) for modelled Universal Basic Income (UBI) policies from 2022-2026

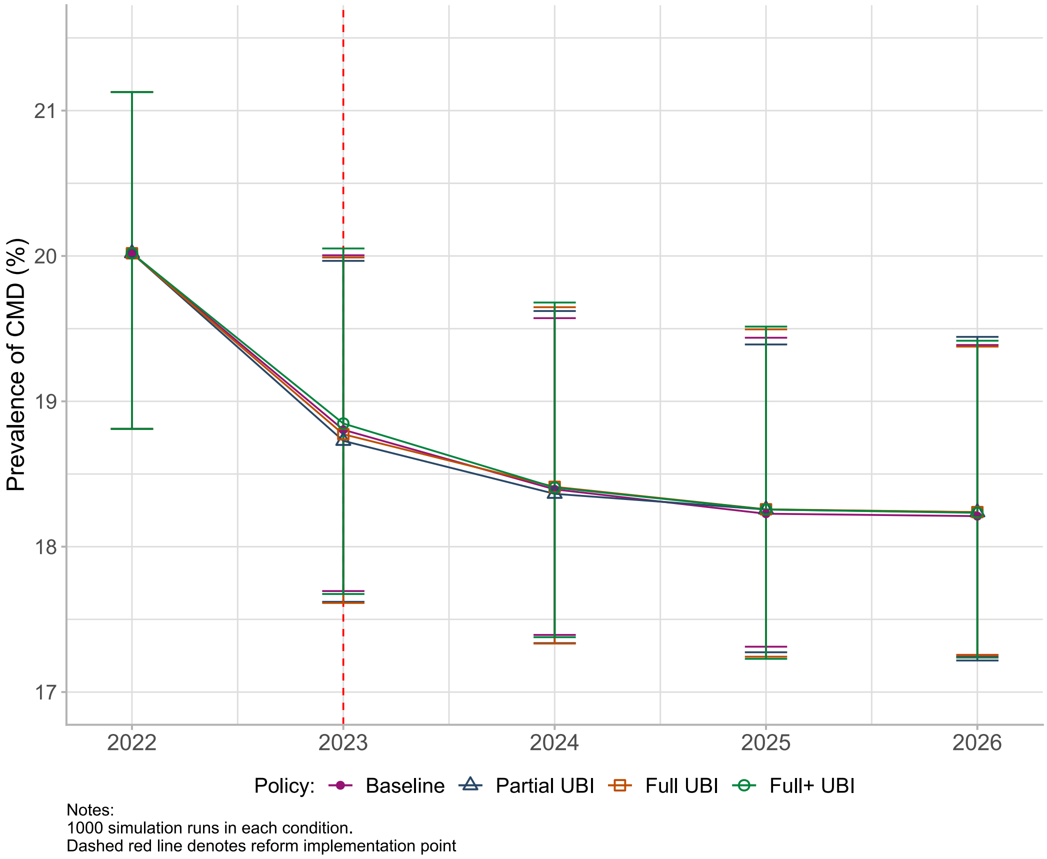

Baseline = planned tax/benefit policies for United Kingdom. Partial UBI = UBI set at level of existing benefits. Full UBI = UBI set at level of Minimum Income Standard. Full+ UBI = Minimum Income Standard plus means-tested benefits for caring, childcare, disability, housing, limited capability for work. Whiskers = 95% Uncertainty intervals.

Table M: Analytical Sensitivity Analyses - Prevalence of common mental disorders and mental health inequalities in baseline scenario and three simulated Universal Basic Income (UBI) scenarios from 2022-2026 (95% uncertainty intervals)

Variable	Scenario	2022	2023	2024	2025	2026
**Prevalence of CMD (%)**	Baseline	20.03 (18.81, 21.18)	18.80 (17.67, 20.00)	18.39 (17.38, 19.57)	18.22 (17.19, 19.44)	18.19 (17.11, 19.38)
	Partial	20.03 (18.81, 21.18)	18.73 (17.60, 19.97)	18.36 (17.28, 19.62)	18.25 (17.19, 19.39)	18.23 (17.17, 19.43)
		Difference:	-0.07 (-0.18, 0.02)	-0.02 (-0.80, 0.75)	-0.01 (-0.83, 0.86)	0.02 (-0.80, 0.78)
	Full	20.03 (18.81, 21.18)	18.77 (17.61, 19.99)	18.41 (17.31, 19.65)	18.26 (17.20, 19.49)	18.23 (17.15, 19.38)
		Difference:	-0.03 (-0.21, 0.13)	0.00 (-0.80, 0.81)	0.03 (-0.83, 0.86)	0.03 (-0.85, 0.88)
	Full+	20.03 (18.81, 21.18)	18.85 (17.67, 20.05)	18.40 (17.36, 19.68)	18.25 (17.18, 19.51)	18.23 (17.15, 19.41)
		Difference:	0.05 (-0.14, 0.22)	0.04 (-0.74, 0.84)	0.03 (-0.82, 0.88)	0.04 (-0.80, 0.87)
**Relative index of inequality**	Baseline	1.44 (1.24, 1.66)	1.37 (1.15, 1.61)	1.30 (1.10, 1.53)	1.24 (1.05, 1.47)	1.21 (1.02, 1.43)
	Partial	1.44 (1.24, 1.66)	1.36 (1.15, 1.60)	1.30 (1.10, 1.54)	1.24 (1.04, 1.48)	1.20 (1.01, 1.44)
		Difference:	-0.00 (-0.04, 0.02)	-0.00 (-0.25, 0.24)	0.01 (-0.25, 0.26)	0.00 (-0.24, 0.24)
	Full	1.44 (1.24, 1.66)	1.35 (1.14, 1.59)	1.30 (1.11, 1.53)	1.24 (1.04, 1.46)	1.20 (1.00, 1.42)

		Difference:	-0.02 (-0.06, 0.02)	-0.01 (-0.25, 0.25)	0.01 (-0.24, 0.25)	-0.00 (-0.25, 0.23)
	Full+	1.44 (1.24, 1.66)	1.35 (1.14, 1.59)	1.30 (1.09, 1.54)	1.24 (1.05, 1.45)	1.20 (1.00, 1.42)
		Difference:	-0.02 (-0.06, 0.02)	-0.01 (-0.27, 0.24)	-0.00 (-0.25, 0.25)	-0.01 (-0.25, 0.25)
**Slope index of inequality**	Baseline	0.07 (0.04, 0.10)	0.06 (0.03, 0.09)	0.05 (0.02, 0.08)	0.04 (0.01, 0.07)	0.03 (0.00, 0.06)
	Partial	0.07 (0.04, 0.10)	0.06 (0.03, 0.09)	0.05 (0.02, 0.08)	0.04 (0.01, 0.07)	0.03 (0.00, 0.07)
		Difference:	-0.00 (-0.01, 0.00)	0.00 (-0.03, 0.03)	0.00 (-0.04, 0.04)	0.00 (-0.04, 0.04)
	Full	0.07 (0.04, 0.10)	0.06 (0.02, 0.09)	0.05 (0.02, 0.08)	0.04 (0.01, 0.07)	0.03 (0.00, 0.06)
		Difference:	-0.00 (-0.01, 0.00)	-0.00 (-0.03, 0.03)	0.00 (-0.03, 0.04)	0.00 (-0.04, 0.04)
	Full+	0.07 (0.04, 0.10)	0.06 (0.02, 0.08)	0.05 (0.02, 0.08)	0.04 (0.01, 0.07)	0.03 (0.00, 0.06)
		Difference:	-0.00 (-0.01, 0.00)	-0.00 (-0.04, 0.03)	-0.00 (-0.04, 0.03)	-0.00 (-0.04, 0.04)

Baseline = planned tax/benefit policies for United Kingdom. Partial UBI = UBI set at level of existing benefits. Full UBI = UBI set at level of Minimum Income Standard. Full+ UBI = Minimum Income Standard plus means-tested benefits for caring, childcare, disability, housing, limited capability for work.

Figure P: Analytical Sensitivity Analysis, using alternative estimates from systematic reviews – Estimated relative (left panel) and slope (right panel) indices of inequality by education for common mental disorder (CMD) in modelled Universal Basic Income (UBI) policies from 2022-2026

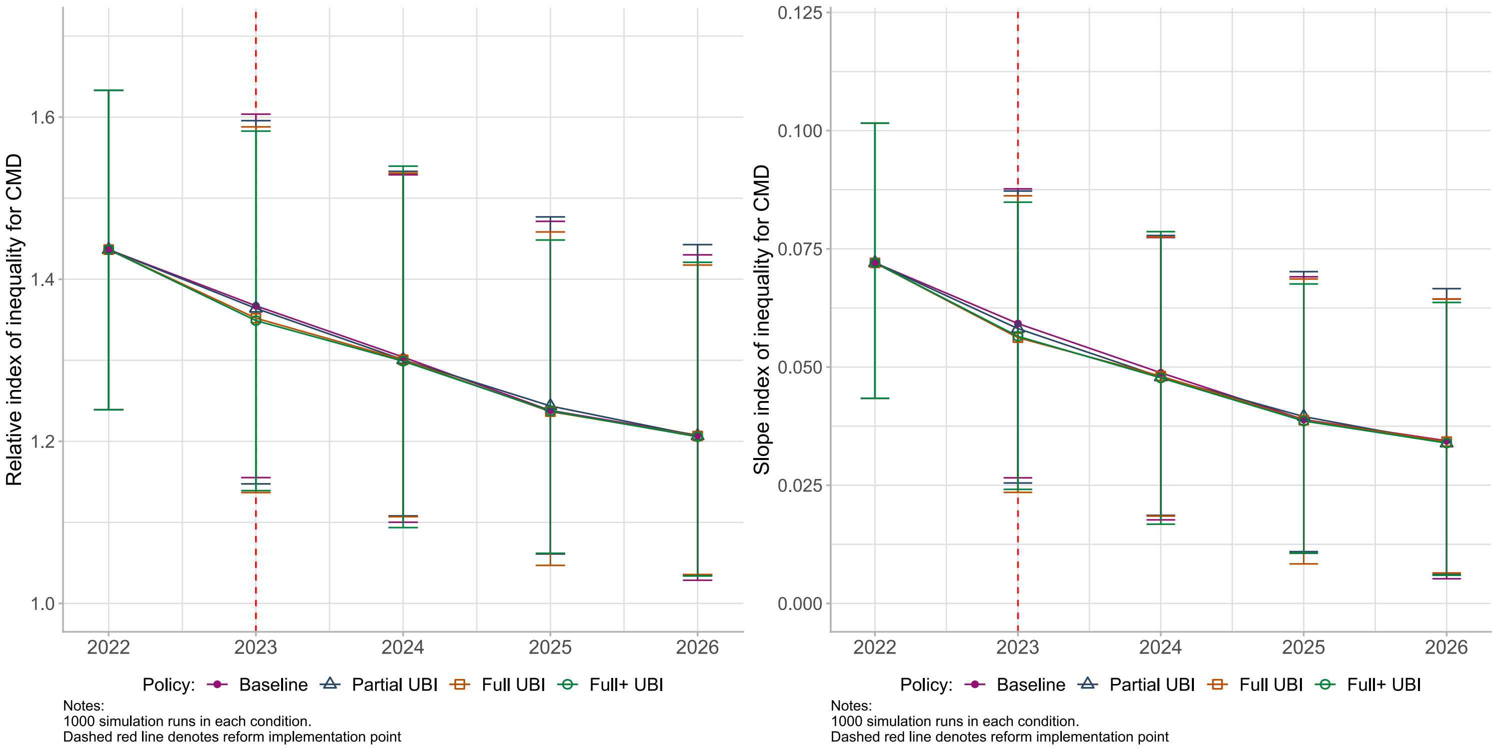

Baseline = planned tax/benefit policies for United Kingdom. Partial UBI = UBI set at level of existing benefits. Full UBI = UBI set at level of Minimum Income Standard. Full+ UBI = Minimum Income Standard plus means-tested benefits for caring, childcare, disability, housing, limited capability for work. Whiskers = 95% Uncertainty intervals.

Results for secondary outcome (GHQ Likert score)

Table N: Estimated GHQ Likert score in baseline scenario and three simulated Universal Basic Income (UBI) scenarios from 2022-2026 (95% uncertainty intervals)

Variable	Scenario	2022	2023	2024	2025	2026
Primary analysis	Baseline	10.37 (9.73, 11.04)	10.73 (10.37, 11.10)	10.93 (10.66, 11.18)	11.00 (10.77, 11.22)	11.05 (10.84, 11.25)
	Partial	10.37 (9.73, 11.04)	10.70 (10.33, 11.08)	10.91 (10.65, 11.16)	11.00 (10.78, 11.21)	11.06 (10.85, 11.27)
		Difference:	-0.03 (-0.04, -0.02)	-0.01 (-0.11, 0.08)	0.00 (-0.11, 0.10)	0.00 (-0.10, 0.12)
	Full	10.37 (9.73, 11.04)	10.74 (10.37, 11.13)	10.91 (10.64, 11.18)	10.99 (10.74, 11.21)	11.02 (10.81, 11.24)
		Difference:	0.01 (-0.01, 0.03)	-0.01 (-0.10, 0.09)	-0.01 (-0.12, 0.09)	-0.02 (-0.14, 0.09)
	Full+	10.37 (9.73, 11.04)	10.76 (10.39, 11.16)	10.91 (10.64, 11.17)	10.97 (10.74, 11.21)	11.01 (10.79, 11.24)
		Difference:	0.03 (0.00, 0.06)	-0.01 (-0.11, 0.08)	-0.03 (-0.14, 0.08)	-0.04 (-0.16, 0.07)
Structural sensitivity analysis: Relaxing employment assumptions	Baseline	10.37 (9.73, 11.04)	10.73 (10.37, 11.10)	10.93 (10.66, 11.18)	11.00 (10.77, 11.22)	11.05 (10.84, 11.25)
	Partial	10.37 (9.73, 11.04)	10.70 (10.33, 11.08)	10.91 (10.65, 11.16)	11.00 (10.78, 11.21)	11.06 (10.85, 11.27)
		Difference:	-0.03 (-0.04, -0.02)	-0.01 (-0.11, 0.08)	0.00 (-0.11, 0.10)	0.00 (-0.10, 0.12)
	Full	10.37 (9.73, 11.04)	10.68 (10.32, 11.07)	10.91 (10.63, 11.15)	11.00 (10.76, 11.23)	11.06 (10.85, 11.26)
		Difference:	-0.05 (-0.07, -0.03)	-0.02 (-0.11, 0.08)	0.00 (-0.11, 0.11)	0.01 (-0.10, 0.12)
	Full+	10.37 (9.73, 11.04)	10.67 (10.31, 11.05)	10.90 (10.64, 11.17)	11.00 (10.79, 11.23)	11.06 (10.84, 11.28)
		Difference:	-0.06 (-0.08, -0.04)	-0.02 (-0.11, 0.08)	0.00 (-0.10, 0.11)	0.01 (-0.10, 0.10)

Baseline = planned tax/benefit policies for United Kingdom. Partial UBI = UBI set at level of existing benefits. Full UBI = UBI set at level of Minimum Income Standard. Full+ UBI = Minimum Income Standard plus means-tested benefits for caring, childcare, disability, housing, limited capability for work.

Table O: Estimated GHQ Likert score in baseline scenario and three simulated Universal Basic Income (UBI) scenarios from 2022-2026 stratified by gender, education, age, previous poverty/employment status, and household structure (95% uncertainty intervals)

Subgroup	Scenario	2022	2023	2024	2025	2026
PRIMARY ANALYSIS						
Men	Baseline	10.46 (9.59, 11.37)	10.57 (10.07, 11.09)	10.66 (10.32, 10.99)	10.70 (10.43, 10.98)	10.74 (10.47, 11.01)
	Partial	10.46 (9.59, 11.37)	10.55 (10.04, 11.08)	10.65 (10.32, 10.98)	10.71 (10.43, 10.98)	10.73 (10.47, 10.98)
		Difference:	-0.02 (-0.04, -0.01)	-0.01 (-0.14, 0.14)	0.00 (-0.18, 0.16)	0.00 (-0.17, 0.16)
	Full	10.46 (9.59, 11.37)	10.63 (10.11, 11.14)	10.68 (10.35, 11.03)	10.71 (10.41, 11.00)	10.72 (10.45, 11.01)
		Difference:	0.05 (0.02, 0.08)	0.02 (-0.13, 0.15)	0.00 (-0.16, 0.16)	-0.01 (-0.17, 0.15)
	Full+	10.46 (9.59, 11.37)	10.65 (10.14, 11.17)	10.68 (10.34, 11.01)	10.70 (10.40, 10.98)	10.71 (10.42, 10.99)
		Difference:	0.08 (0.04, 0.12)	0.02 (-0.12, 0.16)	-0.01 (-0.16, 0.14)	-0.02 (-0.20, 0.14)
Women	Baseline	10.30 (9.35, 11.30)	10.88 (10.35, 11.43)	11.19 (10.81, 11.54)	11.30 (10.98, 11.61)	11.38 (11.07, 11.65)
	Partial	10.30 (9.35, 11.30)	10.84 (10.30, 11.40)	11.17 (10.79, 11.54)	11.30 (11.00, 11.62)	11.38 (11.08, 11.68)

		Difference:	-0.04 (-0.06, -0.03)	-0.02 (-0.15, 0.12)	-0.00 (-0.16, 0.17)	0.00 (-0.16, 0.17)
	Full	10.30 (9.35, 11.30)	10.85 (10.31, 11.41)	11.15 (10.78, 11.54)	11.27 (10.94, 11.60)	11.34 (11.02, 11.67)
		Difference:	-0.03 (-0.06, -0.00)	-0.03 (-0.17, 0.11)	-0.04 (-0.19, 0.12)	-0.04 (-0.20, 0.14)
	Full+	10.30 (9.35, 11.30)	10.87 (10.32, 11.43)	11.15 (10.75, 11.54)	11.25 (10.93, 11.59)	11.31 (10.98, 11.65)
		Difference:	-0.02 (-0.05, 0.02)	-0.04 (-0.19, 0.10)	-0.05 (-0.21, 0.11)	-0.06 (-0.24, 0.11)
**Low education**	Baseline	11.00 (10.31, 11.78)	11.32 (10.89, 11.81)	11.46 (11.09, 11.89)	11.48 (11.10, 11.87)	11.50 (11.08, 11.93)
	Partial	11.00 (10.31, 11.78)	11.28 (10.85, 11.78)	11.44 (11.04, 11.84)	11.48 (11.11, 11.86)	11.50 (11.13, 11.90)
		Difference:	-0.04 (-0.06, -0.02)	-0.02 (-0.35, 0.30)	-0.00 (-0.39, 0.41)	0.01 (-0.44, 0.44)
	Full	11.00 (10.31, 11.78)	11.29 (10.85, 11.80)	11.45 (11.02, 11.84)	11.47 (11.10, 11.88)	11.48 (11.09, 11.88)
		Difference:	-0.03 (-0.06, 0.01)	-0.02 (-0.38, 0.33)	-0.01 (-0.38, 0.38)	-0.02 (-0.47, 0.41)
	Full+	11.00 (10.31, 11.78)	11.31 (10.88, 11.83)	11.43 (11.02, 11.85)	11.45 (11.06, 11.86)	11.45 (11.05, 11.89)
		Difference:	-0.00 (-0.04, 0.04)	-0.04 (-0.37, 0.32)	-0.03 (-0.44, 0.35)	-0.03 (-0.50, 0.43)
**Medium education**	Baseline	10.52 (9.85, 11.23)	10.85 (10.47, 11.23)	11.02 (10.74, 11.29)	11.09 (10.86, 11.32)	11.13 (10.90, 11.36)
	Partial	10.52 (9.85, 11.23)	10.81 (10.43, 11.21)	11.01 (10.72, 11.27)	11.09 (10.84, 11.33)	11.14 (10.91, 11.37)
		Difference:	-0.04 (-0.05, -0.03)	-0.01 (-0.15, 0.12)	0.00 (-0.15, 0.15)	0.01 (-0.15, 0.17)
	Full	10.52 (9.85, 11.23)	10.84 (10.45, 11.24)	11.01 (10.72, 11.30)	11.08 (10.80, 11.32)	11.11 (10.87, 11.35)
		Difference:	-0.01 (-0.03, 0.02)	-0.01 (-0.16, 0.12)	-0.02 (-0.18, 0.14)	-0.02 (-0.18, 0.14)
	Full+	10.52 (9.85, 11.23)	10.86 (10.47, 11.26)	11.00 (10.71, 11.30)	11.06 (10.80, 11.30)	11.09 (10.84, 11.34)
		Difference:	0.02 (-0.02, 0.05)	-0.01 (-0.16, 0.12)	-0.03 (-0.18, 0.13)	-0.04 (-0.21, 0.12)
**High education**	Baseline	9.95 (9.29, 10.62)	10.38 (10.01, 10.79)	10.64 (10.33, 10.92)	10.75 (10.48, 11.01)	10.83 (10.56, 11.08)
	Partial	9.95 (9.29, 10.62)	10.36 (9.98, 10.77)	10.63 (10.34, 10.92)	10.75 (10.49, 11.01)	10.82 (10.57, 11.09)
		Difference:	-0.03 (-0.04, -0.01)	-0.01 (-0.17, 0.16)	0.00 (-0.19, 0.18)	-0.00 (-0.20, 0.20)
	Full	9.95 (9.29, 10.62)	10.42 (10.04, 10.84)	10.64 (10.35, 10.94)	10.74 (10.46, 11.02)	10.80 (10.54, 11.08)
		Difference:	0.04 (0.02, 0.07)	0.00 (-0.17, 0.17)	-0.01 (-0.20, 0.18)	-0.02 (-0.22, 0.19)
	Full+	9.95 (9.29, 10.62)	10.44 (10.06, 10.85)	10.63 (10.33, 10.94)	10.72 (10.46, 11.00)	10.78 (10.52, 11.06)
		Difference:	0.06 (0.04, 0.09)	-0.00 (-0.17, 0.17)	-0.02 (-0.21, 0.16)	-0.04 (-0.23, 0.16)
**Age group: 25-44 years**	Baseline	10.33 (9.71, 11.04)	10.77 (10.39, 11.15)	10.99 (10.69, 11.26)	11.07 (10.82, 11.30)	11.14 (10.92, 11.37)
	Partial	10.33 (9.71, 11.04)	10.73 (10.35, 11.12)	10.97 (10.70, 11.26)	11.07 (10.83, 11.29)	11.14 (10.92, 11.36)
		Difference:	-0.04 (-0.05, -0.02)	-0.01 (-0.15, 0.11)	0.00 (-0.16, 0.15)	0.00 (-0.15, 0.15)
	Full	10.33 (9.71, 11.04)	10.77 (10.39, 11.18)	10.98 (10.69, 11.26)	11.05 (10.80, 11.30)	11.11 (10.88, 11.35)
		Difference:	0.01 (-0.02, 0.04)	-0.01 (-0.15, 0.13)	-0.02 (-0.16, 0.14)	-0.03 (-0.19, 0.13)
	Full+	10.33 (9.71, 11.04)	10.78 (10.40, 11.19)	10.98 (10.69, 11.25)	11.05 (10.81, 11.29)	11.10 (10.86, 11.34)
		Difference:	0.02 (-0.01, 0.05)	-0.01 (-0.14, 0.12)	-0.02 (-0.18, 0.14)	-0.03 (-0.20, 0.14)
**Age group: 45-64 years**	Baseline	10.41 (9.77, 11.09)	10.68 (10.31, 11.08)	10.86 (10.56, 11.12)	10.92 (10.67, 11.16)	10.96 (10.72, 11.19)
	Partial	10.41 (9.77, 11.09)	10.65 (10.27, 11.06)	10.84 (10.56, 11.13)	10.93 (10.67, 11.17)	10.96 (10.72, 11.21)

		Difference:	-0.03 (-0.04, -0.02)	-0.01 (-0.15, 0.13)	-0.00 (-0.16, 0.16)	0.00 (-0.15, 0.17)
	Full	10.41 (9.77, 11.09)	10.69 (10.30, 11.10)	10.84 (10.56, 11.12)	10.92 (10.65, 11.16)	10.94 (10.68, 11.18)
		Difference:	0.01 (-0.02, 0.03)	-0.01 (-0.15, 0.14)	-0.01 (-0.18, 0.17)	-0.02 (-0.19, 0.15)
	Full+	10.41 (9.77, 11.09)	10.72 (10.34, 11.13)	10.84 (10.56, 11.13)	10.89 (10.60, 11.16)	10.90 (10.65, 11.17)
		Difference:	0.04 (0.01, 0.07)	-0.01 (-0.16, 0.14)	-0.04 (-0.20, 0.13)	-0.05 (-0.21, 0.13)
**Have children**	Baseline	10.20 (9.54, 10.89)	10.69 (10.26, 11.12)	10.94 (10.58, 11.29)	11.05 (10.71, 11.37)	11.11 (10.79, 11.44)
	Partial	10.20 (9.54, 10.89)	10.64 (10.20, 11.09)	10.92 (10.56, 11.28)	11.03 (10.70, 11.36)	11.10 (10.78, 11.45)
		Difference:	-0.04 (-0.06, -0.03)	-0.02 (-0.21, 0.15)	-0.00 (-0.21, 0.19)	-0.01 (-0.20, 0.20)
	Full	10.20 (9.54, 10.89)	10.71 (10.26, 11.15)	10.92 (10.55, 11.29)	11.01 (10.67, 11.36)	11.06 (10.73, 11.39)
		Difference:	0.02 (-0.01, 0.06)	-0.02 (-0.19, 0.16)	-0.04 (-0.23, 0.16)	-0.04 (-0.25, 0.15)
	Full+	10.20 (9.54, 10.89)	10.73 (10.29, 11.17)	10.92 (10.55, 11.27)	11.01 (10.67, 11.34)	11.04 (10.71, 11.37)
		Difference:	0.04 (0.00, 0.08)	-0.03 (-0.20, 0.16)	-0.05 (-0.24, 0.15)	-0.07 (-0.26, 0.13)
**No children**	Baseline	10.46 (9.83, 11.15)	10.75 (10.38, 11.14)	10.91 (10.63, 11.18)	10.98 (10.73, 11.21)	11.02 (10.80, 11.24)
	Partial	10.46 (9.83, 11.15)	10.72 (10.35, 11.11)	10.90 (10.65, 11.16)	10.98 (10.76, 11.20)	11.03 (10.81, 11.25)
		Difference:	-0.03 (-0.04, -0.02)	-0.01 (-0.12, 0.11)	0.00 (-0.14, 0.14)	0.01 (-0.12, 0.14)
	Full	10.46 (9.83, 11.15)	10.75 (10.38, 11.15)	10.91 (10.63, 11.18)	10.97 (10.72, 11.22)	11.01 (10.78, 11.24)
		Difference:	0.00 (-0.02, 0.02)	-0.00 (-0.12, 0.12)	-0.00 (-0.15, 0.14)	-0.01 (-0.15, 0.13)
	Full+	10.46 (9.83, 11.15)	10.77 (10.40, 11.18)	10.91 (10.64, 11.19)	10.95 (10.72, 11.22)	11.00 (10.75, 11.23)
		Difference:	0.02 (-0.00, 0.05)	-0.00 (-0.13, 0.11)	-0.02 (-0.16, 0.12)	-0.02 (-0.17, 0.12)
**Lone parent**	Baseline	11.47 (10.39, 12.55)	11.69 (10.99, 12.48)	11.91 (11.23, 12.61)	11.94 (11.20, 12.65)	11.94 (11.28, 12.63)
	Partial	11.47 (10.39, 12.55)	11.66 (10.95, 12.45)	11.91 (11.27, 12.58)	11.96 (11.28, 12.65)	11.98 (11.29, 12.65)
		Difference:	-0.03 (-0.08, 0.02)	-0.00 (-0.70, 0.64)	0.03 (-0.73, 0.81)	0.02 (-0.72, 0.76)
	Full	11.47 (10.39, 12.55)	11.65 (10.93, 12.44)	11.87 (11.15, 12.53)	11.89 (11.20, 12.55)	11.89 (11.21, 12.54)
		Difference:	-0.04 (-0.10, 0.01)	-0.06 (-0.70, 0.60)	-0.05 (-0.82, 0.70)	-0.05 (-0.78, 0.71)
	Full+	11.47 (10.39, 12.55)	11.68 (10.95, 12.46)	11.84 (11.14, 12.56)	11.86 (11.19, 12.53)	11.85 (11.17, 12.51)
		Difference:	-0.02 (-0.08, 0.04)	-0.08 (-0.72, 0.57)	-0.09 (-0.80, 0.66)	-0.10 (-0.85, 0.62)
**STRUCTURAL SENSITIVITY ANALYSIS 1: RELAXING EMPLOYMENT ASSUMPTIONS**						
**Men**	Baseline	10.46 (9.59, 11.37)	10.57 (10.07, 11.09)	10.66 (10.32, 10.99)	10.70 (10.43, 10.98)	10.74 (10.47, 11.01)
	Partial	10.46 (9.59, 11.37)	10.55 (10.04, 11.08)	10.65 (10.32, 10.98)	10.71 (10.43, 10.98)	10.73 (10.47, 10.98)
		Difference:	-0.02 (-0.04, -0.01)	-0.01 (-0.14, 0.14)	0.00 (-0.18, 0.16)	0.00 (-0.17, 0.16)
	Full	10.46 (9.59, 11.37)	10.55 (10.02, 11.07)	10.66 (10.32, 10.99)	10.71 (10.44, 10.97)	10.74 (10.49, 11.00)
		Difference:	-0.03 (-0.06, -0.00)	-0.00 (-0.14, 0.13)	0.01 (-0.15, 0.16)	0.01 (-0.16, 0.18)
	Full+	10.46 (9.59, 11.37)	10.54 (10.01, 11.06)	10.65 (10.31, 10.99)	10.71 (10.44, 10.99)	10.74 (10.47, 11.02)
		Difference:	-0.04 (-0.07, -0.01)	-0.01 (-0.14, 0.13)	0.01 (-0.15, 0.16)	0.01 (-0.15, 0.17)
**Women**	Baseline	10.30 (9.35, 11.30)	10.88 (10.35, 11.43)	11.19 (10.81, 11.54)	11.30 (10.98, 11.61)	11.38 (11.07, 11.65)

	Partial	10.30 (9.35, 11.30)	10.84 (10.30, 11.40)	11.17 (10.79, 11.54)	11.30 (11.00, 11.62)	11.38 (11.08, 11.68)
		Difference:	-0.04 (-0.06, -0.03)	-0.02 (-0.15, 0.12)	-0.00 (-0.16, 0.17)	0.00 (-0.16, 0.17)
	Full	10.30 (9.35, 11.30)	10.81 (10.27, 11.38)	11.15 (10.80, 11.51)	11.30 (10.97, 11.62)	11.37 (11.08, 11.69)
		Difference:	-0.07 (-0.11, -0.04)	-0.03 (-0.17, 0.11)	-0.01 (-0.16, 0.17)	0.00 (-0.16, 0.18)
	Full+	10.30 (9.35, 11.30)	10.80 (10.26, 11.37)	11.16 (10.79, 11.54)	11.30 (10.99, 11.63)	11.39 (11.07, 11.69)
		Difference:	-0.08 (-0.12, -0.05)	-0.03 (-0.17, 0.12)	0.00 (-0.16, 0.17)	0.01 (-0.16, 0.17)
**Low education**	Baseline	11.00 (10.31, 11.78)	11.32 (10.89, 11.81)	11.46 (11.09, 11.89)	11.48 (11.10, 11.87)	11.50 (11.08, 11.93)
	Partial	11.00 (10.31, 11.78)	11.28 (10.85, 11.78)	11.44 (11.04, 11.84)	11.48 (11.11, 11.86)	11.50 (11.13, 11.90)
		Difference:	-0.04 (-0.06, -0.02)	-0.02 (-0.35, 0.30)	-0.00 (-0.39, 0.41)	0.01 (-0.44, 0.44)
	Full	11.00 (10.31, 11.78)	11.23 (10.78, 11.73)	11.42 (11.02, 11.85)	11.47 (11.09, 11.88)	11.52 (11.11, 11.91)
		Difference:	-0.09 (-0.12, -0.05)	-0.04 (-0.37, 0.32)	-0.00 (-0.38, 0.39)	0.01 (-0.43, 0.43)
	Full+	11.00 (10.31, 11.78)	11.23 (10.77, 11.73)	11.42 (11.02, 11.85)	11.47 (11.10, 11.86)	11.52 (11.09, 11.92)
		Difference:	-0.09 (-0.13, -0.06)	-0.04 (-0.38, 0.30)	0.00 (-0.37, 0.36)	0.02 (-0.41, 0.43)
**Medium education**	Baseline	10.52 (9.85, 11.23)	10.85 (10.47, 11.23)	11.02 (10.74, 11.29)	11.09 (10.86, 11.32)	11.13 (10.90, 11.36)
	Partial	10.52 (9.85, 11.23)	10.81 (10.43, 11.21)	11.01 (10.72, 11.27)	11.09 (10.84, 11.33)	11.14 (10.91, 11.37)
		Difference:	-0.04 (-0.05, -0.03)	-0.01 (-0.15, 0.12)	0.00 (-0.15, 0.15)	0.01 (-0.15, 0.17)
	Full	10.52 (9.85, 11.23)	10.78 (10.39, 11.18)	11.00 (10.71, 11.26)	11.10 (10.84, 11.33)	11.14 (10.88, 11.38)
		Difference:	-0.07 (-0.09, -0.04)	-0.02 (-0.16, 0.11)	0.00 (-0.16, 0.17)	0.01 (-0.15, 0.17)
	Full+	10.52 (9.85, 11.23)	10.77 (10.38, 11.17)	11.00 (10.72, 11.29)	11.09 (10.83, 11.34)	11.15 (10.92, 11.38)
		Difference:	-0.08 (-0.10, -0.05)	-0.02 (-0.17, 0.12)	0.00 (-0.15, 0.15)	0.01 (-0.15, 0.16)
**High education**	Baseline	9.95 (9.29, 10.62)	10.38 (10.01, 10.79)	10.64 (10.33, 10.92)	10.75 (10.48, 11.01)	10.83 (10.56, 11.08)
	Partial	9.95 (9.29, 10.62)	10.36 (9.98, 10.77)	10.63 (10.34, 10.92)	10.75 (10.49, 11.01)	10.82 (10.57, 11.09)
		Difference:	-0.03 (-0.04, -0.01)	-0.01 (-0.17, 0.16)	0.00 (-0.19, 0.18)	-0.00 (-0.20, 0.20)
	Full	9.95 (9.29, 10.62)	10.36 (9.99, 10.77)	10.63 (10.33, 10.92)	10.75 (10.48, 11.02)	10.83 (10.57, 11.07)
		Difference:	-0.02 (-0.04, 0.01)	-0.00 (-0.17, 0.17)	0.00 (-0.19, 0.20)	0.01 (-0.19, 0.20)
	Full+	9.95 (9.29, 10.62)	10.35 (9.98, 10.76)	10.63 (10.33, 10.93)	10.75 (10.51, 11.03)	10.83 (10.57, 11.08)
		Difference:	-0.03 (-0.05, -0.00)	-0.00 (-0.19, 0.17)	0.00 (-0.18, 0.19)	0.01 (-0.19, 0.20)
**Age group: 25-44 years**	Baseline	10.33 (9.71, 11.04)	10.77 (10.39, 11.15)	10.99 (10.69, 11.26)	11.07 (10.82, 11.30)	11.14 (10.92, 11.37)
	Partial	10.33 (9.71, 11.04)	10.73 (10.35, 11.12)	10.97 (10.70, 11.26)	11.07 (10.83, 11.29)	11.14 (10.92, 11.36)
		Difference:	-0.04 (-0.05, -0.02)	-0.01 (-0.15, 0.11)	0.00 (-0.16, 0.15)	0.00 (-0.15, 0.15)
	Full	10.33 (9.71, 11.04)	10.72 (10.34, 11.12)	10.97 (10.69, 11.25)	11.07 (10.81, 11.31)	11.14 (10.90, 11.36)
		Difference:	-0.04 (-0.07, -0.02)	-0.01 (-0.15, 0.11)	0.00 (-0.15, 0.15)	0.01 (-0.16, 0.16)
	Full+	10.33 (9.71, 11.04)	10.71 (10.32, 11.11)	10.97 (10.69, 11.25)	11.07 (10.83, 11.33)	11.15 (10.91, 11.37)
		Difference:	-0.06 (-0.08, -0.03)	-0.02 (-0.16, 0.12)	0.01 (-0.15, 0.15)	0.02 (-0.14, 0.16)
	Baseline	10.41 (9.77, 11.09)	10.68 (10.31, 11.08)	10.86 (10.56, 11.12)	10.92 (10.67, 11.16)	10.96 (10.72, 11.19)

**Age group: 45-64 years**	Partial	10.41 (9.77, 11.09)	10.65 (10.27, 11.06)	10.84 (10.56, 11.13)	10.93 (10.67, 11.17)	10.96 (10.72, 11.21)
		Difference:	-0.03 (-0.04, -0.02)	-0.01 (-0.15, 0.13)	-0.00 (-0.16, 0.16)	0.00 (-0.15, 0.17)
	Full	10.41 (9.77, 11.09)	10.62 (10.24, 11.03)	10.84 (10.54, 11.10)	10.93 (10.67, 11.18)	10.97 (10.72, 11.20)
		Difference:	-0.06 (-0.09, -0.03)	-0.02 (-0.15, 0.13)	0.00 (-0.16, 0.17)	0.02 (-0.16, 0.17)
	Full+	10.41 (9.77, 11.09)	10.62 (10.24, 11.03)	10.84 (10.53, 11.12)	10.93 (10.67, 11.16)	10.97 (10.71, 11.22)
		Difference:	-0.06 (-0.09, -0.03)	-0.02 (-0.16, 0.12)	-0.00 (-0.17, 0.17)	0.01 (-0.16, 0.18)
**Have children**	Baseline	10.20 (9.54, 10.89)	10.69 (10.26, 11.12)	10.94 (10.58, 11.29)	11.05 (10.71, 11.37)	11.11 (10.79, 11.44)
	Partial	10.20 (9.54, 10.89)	10.64 (10.20, 11.09)	10.92 (10.56, 11.28)	11.03 (10.70, 11.36)	11.10 (10.78, 11.45)
		Difference:	-0.04 (-0.06, -0.03)	-0.02 (-0.21, 0.15)	-0.00 (-0.21, 0.19)	-0.01 (-0.20, 0.20)
	Full	10.20 (9.54, 10.89)	10.65 (10.21, 11.10)	10.92 (10.54, 11.26)	11.03 (10.71, 11.38)	11.10 (10.77, 11.43)
		Difference:	-0.03 (-0.07, -0.00)	-0.03 (-0.20, 0.15)	-0.01 (-0.19, 0.18)	-0.01 (-0.20, 0.19)
	Full+	10.20 (9.54, 10.89)	10.64 (10.20, 11.09)	10.91 (10.57, 11.27)	11.03 (10.70, 11.38)	11.10 (10.77, 11.45)
		Difference:	-0.04 (-0.08, -0.01)	-0.03 (-0.19, 0.16)	-0.01 (-0.19, 0.17)	-0.01 (-0.20, 0.17)
**No children**	Baseline	10.46 (9.83, 11.15)	10.75 (10.38, 11.14)	10.91 (10.63, 11.18)	10.98 (10.73, 11.21)	11.02 (10.80, 11.24)
	Partial	10.46 (9.83, 11.15)	10.72 (10.35, 11.11)	10.90 (10.65, 11.16)	10.98 (10.76, 11.20)	11.03 (10.81, 11.25)
		Difference:	-0.03 (-0.04, -0.02)	-0.01 (-0.12, 0.11)	0.00 (-0.14, 0.14)	0.01 (-0.12, 0.14)
	Full	10.46 (9.83, 11.15)	10.69 (10.31, 11.08)	10.91 (10.62, 11.17)	10.99 (10.75, 11.21)	11.04 (10.82, 11.26)
		Difference:	-0.06 (-0.08, -0.04)	-0.01 (-0.13, 0.11)	0.01 (-0.13, 0.15)	0.02 (-0.13, 0.16)
	Full+	10.46 (9.83, 11.15)	10.68 (10.30, 11.08)	10.90 (10.63, 11.18)	10.99 (10.75, 11.23)	11.04 (10.82, 11.26)
		Difference:	-0.07 (-0.09, -0.04)	-0.01 (-0.13, 0.11)	0.01 (-0.14, 0.16)	0.03 (-0.12, 0.16)
**Lone parent**	Baseline	11.47 (10.39, 12.55)	11.69 (10.99, 12.48)	11.91 (11.23, 12.61)	11.94 (11.20, 12.65)	11.94 (11.28, 12.63)
	Partial	11.47 (10.39, 12.55)	11.66 (10.95, 12.45)	11.91 (11.27, 12.58)	11.96 (11.28, 12.65)	11.98 (11.29, 12.65)
		Difference:	-0.03 (-0.08, 0.02)	-0.00 (-0.70, 0.64)	0.03 (-0.73, 0.81)	0.02 (-0.72, 0.76)
	Full	11.47 (10.39, 12.55)	11.61 (10.89, 12.39)	11.83 (11.16, 12.50)	11.90 (11.22, 12.60)	11.92 (11.27, 12.61)
		Difference:	-0.08 (-0.14, -0.02)	-0.08 (-0.70, 0.55)	-0.03 (-0.73, 0.73)	-0.02 (-0.73, 0.67)
	Full+	11.47 (10.39, 12.55)	11.61 (10.89, 12.39)	11.83 (11.16, 12.53)	11.91 (11.24, 12.57)	11.90 (11.23, 12.61)
		Difference:	-0.08 (-0.15, -0.01)	-0.07 (-0.71, 0.54)	-0.04 (-0.71, 0.67)	-0.04 (-0.78, 0.67)

Baseline = planned tax/benefit policies for United Kingdom. Partial UBI = UBI set at level of existing benefits. Full UBI = UBI set at level of Minimum Income Standard. Full+ UBI = Minimum Income Standard plus means-tested benefits for caring, childcare, disability, housing, limited capability for work. Low education = no formal qualifications; medium education = Higher/A-level/GCSE [General Certificate of Secondary Education] or equivalent; high education = degree or equivalent.

References

1. Richiardi M, Collado D, Popova D. UKMOD – A new tax-benefit model for the four nations of the UK. International Journal of Microsimulation. 2021;14(1):92-101.
2. Bronka P, van de Ven J, Kopasker D, Katikireddi SV, Richiardi M. SimPaths: An open-source microsimulation model for life course analysis (No. CEMPA6/23). Centre for Microsimulation and Policy Analysis (CeMPA) at the Institute for Social and Economic Research, University of Essex, 2023.
3. Katikireddi SV, Kopasker D, Pearce A, Leyland AH, Rostila M, Richiardi M. Health Equity and Its Economic Determinants (HEED): protocol for a pan-European microsimulation model for health impacts of income and social security policies. BMJ open. 2022;12(7):e062405. doi: 10.1136/bmjopen-2022-062405.
4. Sutherland H, Figari F. EUROMOD: the European Union tax-benefit microsimulation model. International Journal of Microsimulation. 2013;6(1):4-26.
5. van de Ven J, Popova D. UKMOD â€"United Kingdom (UK) country report 2020-2026. Centre for Microsimulation and Policy Analysis at the Institute for Social …, 2023.
6. Centre for Microsimulation and Policy Analysis. Accessing UKMOD [Online] 2023 [cited 2023 February]. Available from: https://www.microsimulation.ac.uk/ukmod/access/.
7. Centre for Microsimulation and Policy Analysis. LABSim Model Description [Online] 2023 [February 2023]. Available from: https://www.microsimulation.ac.uk/jas-mine/demo/labsim/1-labsim-description/.
8. Richiardi MG, Richardson RE. JAS-mine: A new platform for microsimulation and agent-based modelling. International Journal of Microsimulation. 2017;10(1):106-34.
9. Goldberg DP, Williams, P. A user's guide to the General Health Questionnaire. Windsor: NFER-Nelson; 1988.
10. Thomson RM, Kopasker D, Leyland A, Pearce A, Katikireddi SV. Effects of poverty on mental health in the UK working-age population: causal analyses of the UK Household Longitudinal Study. International Journal of Epidemiology. 2023;52(2):512-22. doi: 10.1093/ije/dyac226.
11. Thomson RM, Kopasker D, Leyland A, Pearce A, Katikireddi SV. To what extent does income explain the effect of unemployment on mental health? Mediation analysis in the UK Household Longitudinal Study. Psychol Med. 2022:1-9. Epub 2022/12/01. doi: 10.1017/S0033291722003580.
12. International Labour Organization. ILOSTAT Database Description: Labour Force Statistics: ILOSTAT; 2023 [March 2023]. Available from: https://ilostat.ilo.org/resources/concepts-and-definitions/description-labour-force-statistics/.
13. Thomson RM, Igelström E, Purba AK, Shimonovich M, Thomson H, McCartney G, et al. How do income changes impact on mental health and wellbeing for working-age adults? A systematic review and meta-analysis. The Lancet Public Health. 2022;7(6):e515-e28. doi: 10.1016/S2468-2667(22)00058-5.
14. Hearty W, McCartney G, Paterson M, Adams C, Barclay C, Craig N, et al. Assessing the Feasibility of Citizens' Basic Income Pilots in Scotland: Final Report. Edinburgh: Citizens' Basic Income Feasibility Study Steering Group, 2020.
15. Davis A, Stone J, Blackwell C, Padley M, Shepherd C, Hirsch D. A Minimum Income Standard for the United Kingdom in 2022. York: Joseph Rowntree Foundation; Centre for Research in Social Policy, University of Loughborough, 2022.
16. Gibson M, Hearty W, Craig P. The public health effects of interventions similar to basic income: a scoping review. The Lancet Public Health. 2020;5(3):e165-e76. doi: 10.1016/S2468-2667(20)30005-0.
17. Paul KI, Moser K. Unemployment impairs mental health: Meta-analyses. Journal of Vocational Behavior. 2009;74(3):264-82.
18. Higgins J, Thomas J, Chandler J, Cumpston M, Li T, Page MJ, et al. Cochrane Handbook for Systematic Reviews of Interventions version 6.3 (updated February 2022). United Kingdom: Cochrane; 2022.
19. Eddy DM, Hollingworth W, Caro JJ, Tsevat J, McDonald KM, Wong JB. Model Transparency and Validation: A Report of the ISPOR-SMDM Modeling Good Research Practices Task Force-7. Value in Health. 2012;15(6):843-50. doi: https://doi.org/10.1016/j.jval.2012.04.012.
20. Katikireddi SV. Grant Funding - Understanding the impacts of income and welfare policy responses to COVID-19 on inequalities in mental health: a microsimulation model: The Health Foundation; 2020 [March 2023]. Available from: http://alumni.health.org.uk/funding-and-partnerships/programmes/understanding-the-impacts-of-income-and-welfare-policy-response-covid.
21. NatCen Social Research, University College London, Department of Epidemiology and Public Health. Health Survey for England, 2012-2018 [data collection]. 2nd ed. United Kingdom: UK Data Service; 2018.
22. Welsh Government. Basic income pilot for care leavers: overview of the scheme [Online] 2022 [cited 2023 February]. Available from: https://www.gov.wales/basic-income-pilot-care-leavers-overview-scheme
