# Supplementary material for: Short-term impacts of Universal Basic Income on population mental health inequalities in the UK: A microsimulation modelling study
Source: PLoS Med. 2024 Mar 4;21(3):e1004358. doi: 10.1371/journal.pmed.1004358 (PMC10947674; doi:10.1371/journal.pmed.1004358)
Supplement: Attachment — Submitted filename: PM UBI microsim revision - response letter v2.docx [file pmed.1004358.s002.docx]

*Dear Editors,*

*We would like to extend our sincere thanks to you and the reviewers for the time spent reviewing our paper, your positive comments on the strengths of the study, and your valuable and constructive suggestions. We have revised the manuscript, including the addition of a new secondary outcome as suggested by Reviewer #3, and feel that it is much improved as a result. We provide a point-by-point response to the reviewer and editorial comments below, with our responses in italics throughout.*

**COMMENTS FROM THE REVIEWERS:**

Reviewer #1:

R1.1: This is a well-written paper on an interesting issue of public health and policy salience - UBI is currently of great political interest and the paper makes a contribution to this discourse. As the authors point out, in the absence of rigorous trials of UBI, microsimulation is a valuable tool for exploring the range of potential outcomes that might follow the introduction of a UBI. But, of course, any microsimulation rests heavily on the assumptions being made and this study, although articulating and discussing these limitations, is perhaps less forthright about the resultant very conservative nature of their findings than it might be.

*A1.1: We thank you for your positive comments on the manuscript, and appreciate your concerns regarding the limitations. We believe in our revision we have more clearly discussed these issues, which we respond to directly in relation to your and the other reviewers’ comments below.*

R1.2: The study is well situated in the context of existing literature, although - perhaps because of the timing of submission - recent UK based microsimulation studies of basic income are not referenced:

* Parra-Mujica, F., Johnson, E., Reed, H., Cookson, R. & Johnson, M. (2023) Understanding the relationship between income and mental health among 16- to 24-year-olds: Analysis of 10 waves (2009-2020) of Understanding Society to enable modelling of income interventions A. Moretti (ed.). PLOS ONE. 18(2): e0279845. DOI: 10.1371/journal.pone.0279845

* Reed, H.R., Johnson, M.T., Lansley, S., Johnson, E.A., Stark, G. & Pickett, K.E. (2023) Universal Basic Income is affordable and feasible: evidence from UK economic microsimulation modelling. Journal of Poverty and Social Justice. 31(1): 146-162. DOI: 10.1332/175982721X16702368352393.).

*A1.2: Thank you, you are correct that these papers were not included due to the timing of our submission, though we had referenced the precursor grey literature version of the UBI modelling study. We have now amended the citation to include the more recent version, and included the paper by Parra-Mujica when noting that the effects of income-related policies are likely to be larger for children and young people than for older working-age adults. We have also added the following text to this section to note the concerns you have about our results being overly conservative by excluding this population:*

*“Future modelling work combining these two population groups would be of interest, and beneficial in determining to what extent our estimates may be conservative.”*

R1.3: More attention could also be given to the different pathways through which a UBI might be expected to influence mental health (see, for example, the models of UBI impact in Johnson, M.T., Johnson, E.A., Nettle, D. & Pickett, K.E. (2022) Designing trials of Universal Basic Income for health impact: identifying interdisciplinary questions to address. Journal of Public Health. 44(2): 408-416. DOI: 10.1093/pubmed/fdaa255 and Huss R. Can universal basic income reduce poverty and improve children's health?Archives of Disease in Childhood Published Online First: 30 March 2023. doi: 10.1136/archdischild-2022-324799). These models theorize that the ways in which UBI affects health go far beyond the material effects of income alone and need to be viewed within a broader social determinants of health framework.

*A1.3: We appreciate this comment and do note this as a limitation of our approach throughout the discussion. We have added this citation and also noted our intention to expand our model in future to include some of these additional pathways, while taking care to maintain a causal perspective on the included variables and relationships:*

*“Our model will not capture these mechanisms if they do not act solely through income levels or employment status, potentially underestimating some positive effects of UBI on mental health [53]. In our future work, we intend to expand the included causal pathways to reduce this limitation.”*

R1.4: The methods of the study are well-described. This is a useful case study application of the causally informed mental health module within HEED. The sensitivity analyses related to potential impacts on employment are a strength of the modelling in light of the findings related to employment in the Finnish UBI pilot.

*A1.4: We thank you for these positive comments on the robustness of our approach and inclusion of the sensitivity analyses.*

R1.5: The Discussion section does include some discussion of the two major assumptions of the modelling - that macroeconomic impacts of UBI have no effect on mental health and that income changes due to UBI will have the same mental health impact as other changes in income. These are clearly quite important assumptions, given relationships between income inequality and mental health and indeed the modelled effect on the Gini coefficient (see, for example, Ribeiro, Wagner Silva, et al. "Income inequality and mental illness-related morbidity and resilience: a systematic review and meta-analysis." The Lancet Psychiatry 4.7 (2017): 554-562 and Patel, V., Burns, J.K., Dhingra, M., Tarver, L., Kohrt, B.A. and Lund, C. (2018), Income inequality and depression: a systematic review and meta-analysis of the association and a scoping review of mechanisms. World Psychiatry, 17: 76-89. <https://onlinelibrary.wiley.com/doi/10.1002/wps.20492>) and the likely importance of income stability, increased levels of societal trust and other psycho-social benefits of a true UBI (as per models referenced above). The authors do recognise that their estimates are likely to be conservative, but perhaps don't give the weight to these assumptions in their inference that they might merit. A useful next step would be to incorporate at least the impact on income inequality into future modelling.

*A1.5: We appreciate this comment, and as noted in response to R1.3 above we do have plans to significantly increase the number of included pathways in future iterations of the model, including integration with a macroeconomic model (which has considerable additional complexity). We have therefore added the following text to make this limitation (and our future plans to address it) clearer:*

*“We cannot model interactions between our simulated population and the macroeconomy, and in reality changes to macroeconomic factors such as economic growth, public service provision, and income inequality are highly likely under UBI [20]. Adding these interactions via integration with macroeconomic models is complex [57], but we intend to explore this possibility in future work to overcome this limitation.”*

*We will certainly consider income inequality as one of the potentially important macroeconomic factors to include, though we note that the effect size in the Lancet Psychiatry paper cited here was relatively small, so even if causal this is hopefully unlikely to be a substantial omission from our current work. Of interest, some more recent analyses from our colleagues have suggested it may have a less important role for many health outcomes than initially thought (while other measures of social inequality such as social mobility may be better indicators for health):*

[*https://epiresearch.org/wp-content/uploads/2022/06/2022-Abstract-Book.pdf*](https://epiresearch.org/wp-content/uploads/2022/06/2022-Abstract-Book.pdf) *(04/09 SP – paper in press)*

[*https://academic.oup.com/eurpub/article/33/Supplement_2/ckad160.245/7327988*](https://academic.oup.com/eurpub/article/33/Supplement_2/ckad160.245/7327988)

Reviewer #2:

R2.1: This paper is a microsimulation model of the effect of a universal basic income (UBI) on mental health. This is a relevant topic for PLOS Medicine as there is strong evidence for impact of income on mental health, but limited evidence for the effect of structural interventions that target income on mental health (or other population health outcomes). The methodological approach is appropriate given feasibility issues of implementing a UBI at the population-level, and this paper will complement results from smaller pilots globally that are targeted based on income or other population characteristics. Potential issues with the extent to which these scenarios may be grounded in practical application are addressed through the input of UBI leaders and health and government decision-makers in selecting the parameters of the four UBI scenarios. The assumptions of the model are important for microsimulation to make contributions in this field of research, and while the authors raise the issue of macroeconomic impacts, these were not included in sensitivity analysis. This is the paper’s major weakness and requires either inclusion in the microsimulation or more justification as to why this was not included. I recommend major revisions before this paper can be published with PLOS Medicine.

*A2.1: We thank you for your positive comments on the paper, and hopefully address your concerns in the response to your individual points below.*

Major revisions

R2.2: Methods:

-The authors identify several structural and theoretical assumptions of the model, including that any macroeconomic impacts of the policy intervention would not affect the outcomes. This is a significant weakness of the model that requires further justification and discussion, or to be addressed through an additional sensitivity analysis. It is reasonable to expect that mental health services and supports would be less available with the introduction of a UBI either owing to cut-backs related to UBI financing, or labour markets impacts of people leaving lower-paid jobs social care roles and making care less available. While this may not have impacts on people with more transitory or situational mental health concerns related to work or income stress that may be addressed through the UBI, it could worsen mental health for those who require more fulsome supports.

*A2.2: We entirely appreciate this perspective, and are clear in the paper that this is a key limitation of our current approach. However, the integration of microsimulation models with macroeconomic models (such as computerised general equilibrium [CGE] models) is fairly unusual within economic literature, and highly complex to operationalise – we are unaware of any models which aim to do this while including a health perspective. To give an indication of the extent of this complexity, while we do intend to develop our model in this direction in future, it is likely to take several years to get to this stage of development, meaning it would not be possible to include a sensitivity analysis exploring the topic at this time. We have however added a statement to the Limitations section to this effect which incorporates your point about potential impacts on public service provision, as also noted in response to Reviewer 1:*

*“We cannot model interactions between our simulated population and the macroeconomy, and in reality changes to macroeconomic factors such as economic growth, public service provision, and income inequality are highly likely under UBI [20]. Adding these interactions via integration with macroeconomic models is complex [57], but we intend to explore this possibility in future work to overcome this limitation.”*

R2.3: Minor revisions

Introduction

-The authors indicate that that mental health has been worsening over the past 15 years "against a background of economic crises, austerity policies, and COVID-19", but only one citation shows some empirical evidence for this relationship. Suggest providing more explanation of other trends that are attributable to the increase in mental health disorders (i.e., generational shift in mental health disorders among adolescents and young people, increased use of digital media, etc). It may otherwise be interpreted that greater economic security should result in a more significant decrease in mental disorders than was found through the microsimulation model.

*A2.3: Thank you, we have clarified that we are referring to adult mental health in our statement and that the cited empirical references relate to this:*

*“In the United Kingdom (UK), population mental health in working-age adults has deteriorated over the last fifteen years against a background of economic crises, austerity policies, and COVID-19 [12-15].”*

*We were not sure how to action your point regarding the citations not showing empirical evidence for this relationship, as three of the studies involve primary data analysis looking at the effect of recession, austerity and COVID on mental health, and one paper is a review of studies considering the impact of austerity on health outcomes including mental health. However, if you wish specific changes to the references at further revision stages, we would of course be happy to re-review this.*

*We elected to focus primarily on these economic contextual factors in the Introduction as this is the primary focus of our paper. We also note that evidence on the impact of digital media on mental health remains relatively unclear, with the evidence base primarily focused on adolescents and young people as the Reviewer notes. However, if the editors feel this is relevant to include we would be happy to add this.*

R2.4: -The authors use the definition of a UBI (versus a guaranteed income or basic income) which includes universality, however the examples provided (Finland, Ontario, and individuals leaving the care system) do not meet that criteria. Revise to indicate that these pilots are not a UBI, which would be consistent with the authors statement that "no fully universal UBI has been trialed".

*A2.4: Thank you for noting this, we agree this was an inconsistency. We have amended the wording describing these trials in the Introduction to make clear that they are ‘UBI-like’ policies, despite often being described as UBI by those delivering them:*

*“UBI has also been suggested as a potential approach to reducing health inequalities [8], and recent trials of UBI or UBI-like policies have been undertaken or planned in several high-income countries [10, 11].”*

*“There has been developing interest in the use of UBI-type approaches within the UK’s devolved administrations, with the Scottish Government developing detailed plans for a potential UBI pilot [20] (though this did not move forward to implementation) and the Welsh Government recently introducing a small trial of a basic income for individuals leaving the care system [21].”*

R2.5: -The statement that no fully universal UBI has been trialed is accurate for high-income countries, but some low-income country trials do meet the criteria of universality in smaller geographic areas (e.g., GiveDirectly in rural Kenya). Revise to indicate that no fully universal UBI has been trialed in a high-income context.

*A2.5: We have amended this statement to be limited to high-income countries only.*

R2.6: Methods

-The authors indicate that the mental health module was developed using empirical epidemiological estimates of the effects of economic transitions on mental health - where were estimates derived from?

*A2.6: The causal estimates used are from two recently published papers which we cite in the Methods section. The methodology is briefly described in the section ‘Model input data’, with more detail provided in the Appendix and the cited papers:*

*“The mental health module also uses data from Understanding Society (Table S2, Appendix). The Step 2 causal estimates for transitions between poverty and employment states in the working-age population aged 25-64 years were calculated using double-robust marginal structural modelling; we draw on our previous causal epidemiological analyses (with full methodological details including our causal framework and estimation approach published previously [38, 39]). As effects differed by gender (but not markedly by education level or age group), estimates in the mental health module were calculated separately for men and women.”*

R2.7: Results:

-The authors stratify results based on several sociodemographic factors (gender, education, age, and household structure) but there is no stratification based on level of taxation. Under the partial, full and full+ UBI scenarios, there is a significant change in taxation for those who are currently 'middle income', e.g., 70% from 30K and 85% from 50K for the full and full+ UBI (unless I have misunderstood the income tax rates in Table 1, in which case more explanation is required). I would expect some increase in CMDs is from people who were previously in a higher income bracket but whose income is now lower post-taxation, and so it would be interesting to see if the increases are higher in that group.

*A2.7: We thank you for this interesting comment. While it would certainly be of interest to explore this, unfortunately it was not something we were able to do in our current modelling due to the separation of the UBI modelling within UKMOD (where we would be able to identify someone’s taxation status) and the dynamic health modelling within SimPaths (which does not directly simulate taxes and benefits, but rather matches the gross:net income ratio from UKMOD output to similar simulated individuals and households). However, you raising this point has made us consider whether integrating this into future versions of the model would be useful for additional sensitivity analyses, which we sincerely appreciate.*

R2.8: Discussion:

-The authors state that "there is little evidence UBI-like interventions are associated with large increases in unemployment", however the evidence for minimal impacts is thin. Hum (1993) was published before current labour market trends (e.g., precarity, frictional employment) that could impact the behavioural response to a UBI, and de Paz-Banez (2020) indicates significant methodological weaknesses in what is used as proxies for a UBI. While there is still relatively good consensus that UBI would not impact unemployment, suggest citing more robust literature given how important increases in unemployment were to the sensitivity analysis. I suggest the authors draw from more recent literature (see Marinescu, Skandalis and Zhao; Scott, Altonji et al.) which concluded that COVID-19 benefits resulted in minimal work disincentives from the expansion of benefits during COVID-19. Although these benefits were temporary, they may be of more relevance to the behavioural response to a UBI than the older literature that is cited.

*A2.8: We thank you for drawing our attention to this more recent evidence on work incentives, which we agree is useful additional piece of supporting evidence for our point. We have added these references to the paper:*

*“There is little evidence UBI-like interventions are associated with large increases in unemployment [22], with existing reviews finding few and surprisingly small adverse labour supply effects [38, 50]. This is in keeping with more recent real-world evidence from the introduction of COVID-related benefits, which were found to result in minimal work disincentives in the United States despite considerable expansion compared with the pre-pandemic provision of welfare [51, 52].”*

Reviewer #3:

R3.1: Thank you to the authors for submitting this paper, I very much enjoyed reading it. I definitely think it has the potential for publication in this journal, but I have a number of comments that I think should be addressed or considered before publication. I have listed them in roughly the order they appear in the text and have indicated which comments I think are minor and which are major. I hope the comments are useful and potentially improve the paper.

*A3.1: We thank you for your positive comments on the paper.*

R3.2: Major comment - I think a significant amount of further justification needs to given regarding the choice to dichotomise the GHQ-12 and use this as your primary outcome measure. I completely understand that it is easier to interpret (i.e. you can estimate an approximate cost per common mental disorder avoided), but to me it feels as though you are just throwing away potentially useful information. Although the interpretation of the results would clearly be a little bit more difficult, I think looking of the relationship in a little bit more granular detail would be useful rather than a dummy variable. At the very least I think these results should be presented as part of the online appendix.

*A3.2: Thank you for raising this concern, on reflection we agree with both you and the Editor that we should include the continuous GHQ-36 Likert score in our presentation of the findings. We have gone back to our original modelling output and extracted these data, which we present in two new tables in the Appendix. We were able to present the findings for this secondary outcome for both the main analysis and our main structural sensitivity analysis relaxing employment assumptions, for the whole population and for all stratified groups. The results show very similar patterning to our main analysis, as outlined in this new paragraph in the Results:*

*“Results for our secondary outcome (continuous GHQ Likert score) are shown in Tables S14-S15, Appendix. These were in keeping with the findings for our primary outcome, with results from the main analysis showing higher GHQ Likert scores with Full+ UBI compared to the baseline scenario (0.03 [95% UI 0.00, 0.06]) and the structural sensitivity analysis with reduced employment assumptions showing instead a reduced GHQ score (-0.06 [‑0.08, ‑0.04]). On stratification, as with the primary outcome the positive effects of UBI in this sensitivity analysis were larger for women (-0.08 [-0.12, -0.05]) and those with least education (-0.09 [-0.13, -0.06]), and there were also larger effects for lone parents (-0.08 [‑0.15, -0.01]).”*

*We have also added additional text to the Methods outlining this addition to the methodology:*

*“Our secondary outcome was the 36-point GHQ Likert score, a continuous measure scored from the same questionnaire, with higher scores indicating higher levels of psychological distress.”*

R3.3: Major comment - I fully appreciate that it is a very complicated (and impressive) set of methods, but I really think that further detail in the main text is needed regarding the Simpaths model, especially the transitions between the different states (for example the probabilities and distributions used). I know some of this information is included in the online appendices, but I think it is really needed in the main text in order for the paper to flow. The model comes across as a bit of a black box at the moment.

*A3.3: We appreciate this perspective. In choosing what to include in each section we have tried to balance transparency with concision, to avoid overloading the main manuscript with large amounts of technical detail on the modelling and its validation. For those who wish to explore the model in its full detail, we provide both the Appendix and the online Working Paper describing SimPaths and its validation (https://www.microsimulation.ac.uk/publications/publication-557738/https://www.microsimulation.ac.uk/publications/publication-557738/*[*https://www.microsimulation.ac.uk/publications/publication-557738/*](https://www.microsimulation.ac.uk/publications/publication-557738/)*). However, in response to your concerns we have expanded some of the sections on SimPaths within the Methods section of the main manuscript, as outlined below:*

*“SimPaths evolves a representative sample of the UK population in one-year increments through six modules: Demography, Education, Health, Household composition, Non-labour market income, and Labour supply (Figure 1). It was developed using JAS-mine, an open-source, object-oriented Java-based platform specifically designed for discrete-event simulations. An open-source version of the model is available on GitHub (https://github.com/centreformicrosimulation/SimPaths). Each module includes several sub-processes estimated on longitudinal data external to the simulation [27]. The input data are described below. Alignment within the ageing and fertility processes ensures simulated population demographics do not deviate substantially from official projections from the Office for National Statistics.*

*In the Labour supply module, individuals within households (which may be made of up multiple benefit units) select the number of hours they wish to work based on a structural random utility model, with potential wages calculated using a Heckman-corrected wage equation. This is a non-forward-looking model of labour supply, in which employment decisions are made in order to maximise within-period utility of benefit units, given exogenous hourly wage rates predicted by the wage model. The model is unitary, which means that decision making is at the level of the benefit unit – for example, one single choice involving labour supply decisions for each partner is made by couples, in order to maximise their joint utility.”*

*“SimPaths’ simulated UK population and the parameter estimates for the modules are drawn from the UK Household Longitudinal Study (also called ‘Understanding Society’), a representative panel-based study which includes income, health, and sociodemographic measures [41]. The labour supply module uses utility functions estimated on the data from the Family Resources Survey [40] – these estimates inform the decisions individuals and benefit units take about how to prioritise labour time over leisure time.”*

*“Output produced by the model consists of an SQL database tables and/or CSV files at the individual, benefit unit, and household level, which can be linked through the unique identifiers. The output files contain the values of simulated variables for each individual unit in each year of the simulation, effectively producing a “synthetic” panel dataset.”*

*“SimPaths accounts for parameter uncertainty by including routines that facilitate bootstrapping parameter estimates, based on estimated point values and covariance matrices. This involves repeated simulations, each based on a different random draw for model parameters. Similarly, Montecarlo variation can be explored by conducting repeated simulations each based on fresh set of random draws or by arbitrarily scaling-up the simulated population size. These methods can be used to generate a distribution of model outcomes, around central projections.”*

R3.4: Major comment - I would also like some discussion of the 'causal' methods used in the main text, including the g-computation methods used. They are briefly referenced in the appendix (and weirdly the acknowledgements) but for me this isn't enough. They are an important aspect of the paper so should be in the main text somewhere. This is contributing to the 'black box' feel of the model as it currently reads. I would move Table S2 (and potentially Table S4) from the appendix into the main text.

*A3.4: We have reviewed this section of the manuscript again in response to your comment. Our causal modelling approach is laid out in detail in our two earlier papers referenced in this section, as well as being described in the Appendix. While we understand your perspective, we do not feel that reiterating this detail in the main text is necessary to provide understanding of the model’s processes, though we would be happy to revisit this if the Editor feels strongly about its inclusion. We have reworded the text in the section of the Methods on Model Input Data to signpost the link with the existing papers more explicitly:*

*“The mental health module also uses data from Understanding Society (Table S2, Appendix). The Step 2 causal estimates for transitions between poverty and employment states in the working-age population aged 25-64 years were calculated using double-robust marginal structural modelling; we draw on our previous causal epidemiological analyses (with full methodological details including our causal framework and estimation approach published previously [38, 39]).”*

R3.5: Major comment - The measures of inequality used in certain parts of your analyses (relative index of inequality, slope index of inequality, gini coefficient) need to be explained in the methods. A reader with no background quantitative measures of inequality such as these would have no idea what they mean.

*A3.5: We agree on reflection that this detail would be helpful to include, and have added the following explanations to the Methods section:*

*“Relative and slope indices of inequality (RII/SII) in CMD (by education) were calculated for the whole working-age population, and for men and women separately. These inequality measures regress the outcome for those in a particular socioeconomic group on the proportion of the population that has a higher position in the hierarchy, in this case those with higher education [43]. They can be interpreted as the ratio (RII) or absolute difference (SII) in CMD prevalence between those with the hypothetically least and the most education, taking into account population size.”*

*“The initial UKMOD output also included a calculation of the Gini coefficient for each policy scenario, a commonly used measure of income inequality from 0 (perfect equality) – 1 (perfect inequality) which expresses the expected absolute gap between people’s incomes relative to the mean population income [43].”*

R3.6: Major comment - I think you need to further emphasise the significant level of uncertainty present in the vast majority of the results you present. Looking at Table 2, it looks like your "Main Analysis" in 2023 is the only estimate where the confidence intervals do not contain 0. Can you comment further on the possible reasons for this significant level of uncertainty? Where in the model do you think it is coming from?

*A3.6: We agree that there is considerable uncertainty in many of our results, which reflects the fact that we observed relatively modest effects overall. We have aimed to draw our main inferences from those results which are less uncertain e.g., the findings from the primary analysis and the main structural sensitivity analysis in Table 2, neither of which cross 0. We believe some of the uncertainty likely stems from both the small effect sizes for income/poverty transitions, and the fact we focus on short-term effects of economic transitions, which we note as a limitation. In future versions of the model we hope to expand to consider the longer-term and lagged impacts of poverty and unemployment on health, which we suspect will decrease some of this uncertainty. In response to your comment we have added an acknowledgement of this uncertainty to the first paragraph of the Discussion, and expanded on the potential reasons in the Limitations section:*

*“In both situations impacts appear relatively short-lived, with no meaningful impact on mental health inequalities by education. There is also considerable uncertainty around many of our estimates, particularly after the first year of policy implementation.*

*These findings are driven by the relative importance of income, poverty, and employment for mental health [47].”*

*“We model only short-term (one-year) causal effects of economic transitions and deal with a relatively short time horizon (four years from implementation), potentially underestimating longer-term impacts of the policies and introducing the more marked uncertainty seen around the estimates in the years after the intervention.”*

R3.7: Minor comment - Is the Full UBI (orange) line missing from the left hand panel of Figure 2?

*A3.7: Thank you for noting that this is difficult to interpret. The second line is not actually missing, but the prevalence of poverty with both Full and Full+ UBI were extremely similar and these lines therefore overlap considerably on the graph. We have now added a footnote to make this clearer. We have also made accessibility-related changes to the graphs in response to editorial comments which include the use of different shapes for the point estimates of each policy scenario, which should also improve legibility for this issue.*

R3.8: Minor comment - I would use the same scale for each of the four panels in Figure 4 so it is easier for the reader to compare the different sub-groups. I also personally find the colour scheme difficult to read - that might just be me though!

*A3.8: We appreciate this comment and initially we had designed these graphs with the same scales. However, due to the considerable difference in CMD prevalence in some groups (particularly in those with low education and lone parents) this compression made the individual graphs extremely difficult to interpret at printable size. Therefore, we elected to use scales appropriate for making each panel legible, while being transparent about the fact that this was the case in the Figure title.*

*We have noted the concerns of yourself and the Editor in relation to the graph colours and have reviewed these from an accessibility perspective, with the revised graphs now using colours identified as being colour blind safe by the Adobe Color Accessibility tool online:* [*https://color.adobe.com/create/color-accessibility*](https://color.adobe.com/create/color-accessibility)*.*

R3.9: Minor Comment - In the sensitivity analysis section you note that you "modified the utility values... so employment rates remained constant". I think you need to explain this comment further, what utility values are you talking about? It isn't clear

*A3.9: We believe that the additional information we have added to the Methods section about the labour supply module in response to your earlier comment (R3.3), and the explanation of the data source used for these utility functions, hopefully makes this clearer.*

R3.10: Minor Comment - Is there any particular reason you used the median values in the stochastic uncertainty analysis? I personally have only ever seen the mean values used in these instances. One sentence explaining why you use the median values will probably do.

*A3.10: We decided a priori to use median values rather than the mean to avoid any potential influence of outlying values, and in case outcomes were not normally distributed.*

R3.11: Minor comment - Similar to a previous comment, but in the discussion can you comment on the possible reasons why the impacts on mental health are relatively short lived and don't sustain over the long term?

*A3.11: As noted in response to your previous comment A3.6, we believe this is likely due to the incorporation of short-term effects only in the current version of the model, and have added a comment to this effect in the Limitations.*

R3.12: Minor comment - Does "your recent systematic review" reference much of the economics literature which has used IV/RDD methods to identify a causal impact of income on health? If so I think it would be briefly worth mentioning here the difficulties of using these methods and the drawbacks in terms of interpretation of the results from such studies.

*A3.12: Our systematic review aimed to include all studies looking at the effect of income changes on mental health and wellbeing outcomes, from both epidemiological and economics literature. However, there were only a small number of studies which used an IV approach, and none which used an RDD approach, so we did not feel it was required to add discussion of this to the current paper. Of interest, we also did not find significant differences in effect sizes between RCTs and non-randomised studies on meta-regression, which suggests that our results were not driven by atypical IV findings or other methodological issues.*

R3.13: Minor comment - Are you aware of any applied economic literature which has looked at the willingness to pay of avoiding mental illness/disorders? If there are any papers regarding this it would be good to bring into your discussion to put your "£600,000 per case of CMD avoided" into some context.

*A3.13: Thank you for this very interesting suggestion. We have added a reference to literature on this topic to the sentence on cost effectiveness, and feel that this adds useful context:*

*“In our best-case scenario for mental health outcomes this equates to a cost of just under £600,000 per case of CMD avoided, substantially higher than the approx. £350/month individuals state they would be willing to pay to avoid depression [62], which might suggest such a policy approach should not be pursued based on its mental health benefits alone.”*

Reviewer #4:

R4.1: This is a really interesting and well written paper on the potential impact of implementing a universal basic income on mental health in the UK.

*A4.1: We thank you for your kind comments on the paper’s value.*

R4.2: I would like to commend the authors on their inclusion of PPIE in this study and the role they played in shaping the research and also on moving the focus away from fiscal neutrality. However, I would have liked to have seen fiscal neutrality as a scenario as they is likely needed from a political perspective.

*A4.2: We do appreciate this perspective, and can confirm that it was the strong wish of the project advisory group that the paper did not get drawn into discussions about the ‘best’ way to fund a UBI, but rather focused on examining the health impacts. For these reasons, as outlined in the Appendix, we did not feel it was appropriate to try to model this within our work. It is also worth noting that, within UKMOD, the only tax levers that were available to us to achieve fiscal neutrality were income tax, rather than the multitude of other potential sources that have been proposed to partially or fully fund UBI schemes – therefore, even if we had aimed for fiscal neutrality, this would only have been one (likely quite unrealistic) method of doing so.*

R4.3: I thought the choice of timeframe (5 years) and the introduction on UBI one year into the cycle will help with how the implementation of such a policy would work in reality in the UK. However, would it have been worthwhile looking at the implementation of the partial UBI and then perhaps a transition to a full UBI within the same model run but with a longer time horizon to allow this progression? I believe that any government would more likely follow this route rather than introduce full or full+ straight off. Furthermore, from a policy perspective, I would have also liked to have seen the reverse i.e. to model the successor government post-five years removing the policy and then seeing how quickly the impact is reversed.

*A4.3: This is a very interesting suggestion, and we will certainly bear in mind these more ‘realistic’ implementation scenarios for our future modelling work on UBI and related policies. We are less confident about extrapolating our current model structure to a longer time horizon beyond the initial five year period, but are planning future work (e.g., with integration of a macroeconomic CGE model) to allow this to be done.*

R4.4: Minor point - I would like to see some justification of the use of 1000 simulation runs for each analysis in your uncertainty analysis.

*A4.4: We have included the justification for this choice in the Appendix:*

*“Finally, to be sure that we were running at least the minimum number of simulations likely to appropriately account for parameter and stochastic uncertainty, we examined the variability of the cumulative mean prevalence of two outcomes of interest (CMD and poverty) by number of simulation runs (Figure S2). This confirmed that completing 1000 runs of our simulations was likely to achieve sufficient stability of outputs, as relative stability was achieved at around 250 runs.”*

R4.5: Minor point - table 2: I am not sure anyone finds values of -0.00 helpful - would it be possible to change to > unless the value is actually 0.

*A4.5: We appreciate this concern - we will defer to the Editor about preferences regarding the presentation of these values in tables.*

R4.6: I would also like to see reference to the review by Pinto et al on Exploring different methods to evaluate the impact of basic income interventions: a systematic review in your background prior to introducing the microsimulation approach employed in your paper.

*A4.6: Thank you for providing this citation, which we have now added to the Introduction of the paper.*

**ACADEMIC EDITOR COMMENTS**

A very interesting and relevant study. The authors have done a great job at conveying complex methods in an understandable form, although I support reviewer requests for some further explanations to come into the main paper. One reviewer raised an important concern about using a cut-off on the GHQ, which I agree with. All other review comments seemed pertinent and important.

*We thank you for your kind comments on our study, and agree that both your and the reviewer comments are important to address, which we have aimed to do in our responses above.*

FINANCIAL DISCLOSURE: The funding statement should include: specific grant numbers, initials of authors who received each award, URLs to sponsors’ websites. Also, please state whether any sponsors or funders (other than the named authors) played any role in study design, data collection and analysis, the decision to publish, or preparation of the manuscript. If they had no role in the research, include this sentence: “The funders had no role in study design, data collection and analysis, decision to publish, or preparation of the manuscript.”

*We have added the required sentence to the funding statement.*

COMPETING INTEREST: All authors must declare their relevant competing interests per the PLOS policy, which can be seen here: <https://journals.plos.org/plosmedicine/s/competing-interests>. For authors with ties to industry, please indicate whether any of the interests has a financial stake in the results of the current study.

*Apart from the funding acknowledged, we declare no other competing interests.*

DATA AVAILABILITY STATEMENT: The Data Availability Statement (DAS) requires revision. For each data source used in your study: a) If the data are freely or publicly available, note this and state the location of the data: within the paper, in Supporting Information files, or in a public repository (include the DOI or accession number). b) If the data are owned by a third party but freely available upon request, please note this and state the owner of the data set and contact information for data requests (web or email address). Note that a study author cannot be the contact person for the data.

c) If the data are not freely available, please describe briefly the ethical, legal, or contractual restriction that prevents you from sharing it. Please also include an appropriate contact (web or email address) for inquiries (again, this cannot be a study author).

*We have updated the data availability statement accordingly.*

ABSTRACT

1) PLOS Medicine requests that main results are quantified with 95% CIs as well as p values. When a p value is given, please specify the statistical test used to determine it. When reporting p values please report as p<0.001 and where higher as the exact p value p=0.002, for example. For the purposes of transparent data reporting, if not including the aforementioned please clearly state the reasons why not.

*As our results come from 1000 runs of our models, we report 95% uncertainty intervals rather than the results of a statistical test, so there is no associated p-value. We have now made this clearer in the abstract (lines 41 and 44).*

2) Throughout, suggest reporting statistical information as follows to improve clarity for the reader “22% (95% CI [13%,28%]; p</=)”. Please amend throughout the abstract and main manuscript. Please note the use of commas to separate upper and lower bounds, as opposed to hyphens as these can be confused with reporting of negative values.

*We have made this change throughout the abstract and manuscript.*

3) Please ensure that all numbers presented in the abstract are present and identical to numbers presented in the main manuscript text.

*We have checked that these numbers are consistent.*

4) Please include any important dependent variables that are adjusted for in the analyses.

*As this is a simulation study we run multiple regression analyses across many processes, which are outlined within the paper and supplementary appendix. It would unfortunately not be possible to describe all of these models in detail within the abstract of the paper. However, we do outline the key potential effect modifiers that we stratify our results by in the abstract.*

5) Please define ‘UK’ at first use.

*We have now done this.*

6) Please add a unit when discussing age (years, months etc.).

*We have now done this.*

7) Abstract Methods and Findings:

*Please include the number of participants and the length/time frame of follow up/microsimulation model (i.e. what means short-term effect?).

*In the last sentence of the Abstract Methods and Findings section, please describe the main limitation(s) of the study's methodology.

*We have now added this information to the abstract in lines 32 and 35.*

AUTHOR SUMMARY

At this stage, we ask that you include a short, non-technical Author Summary of your research to make findings accessible to a wide audience that includes both scientists and non-scientists. The Author Summary should immediately follow the Abstract in your revised manuscript. This text is subject to editorial change and should be distinct from the scientific abstract. Please see our author guidelines for more information: <https://journals.plos.org/plosmedicine/s/revising-your-manuscript#loc-author-summary> .

The summary should include 2-3 single sentence, individual bullet points under each of the questions. The last bullet under ‘What Do These Findings Mean?’ point should describe the main limitation of the study's methodology.

It may be helpful to review currently published articles for examples which can be found on our website here <https://journals.plos.org/plosmedicine/>

*We have now added the Author Summary on page 3.*

INTRODUCTION

1) Please define ‘UK’ at first use.

*We have now done this.*

2) Please expand your introduction about previous research (e.g., what did recent trials show?) and explain the need for and potential importance of your study.

*In the final paragraph of the Introduction we note that recent trial evidence suggests that UBI may be beneficial for mental health, but that none of these studies assess a ‘true’ UBI, and make the case that this is a situation where a policy modelling approach (such as ours) can be particularly useful. We have also added some additional points to the Introduction in response to reviewer comments 2.3, 2.4, and 4.6.*

METHODS AND RESULTS

1) Please change “(41.6m)” to “(41.6 million)” or define ‘m’ at first use.

*We have now done this.*

2) Please provide a complete list of model parameters, including clear and precise descriptions of the meaning of each parameter, together with the values or ranges for each, with justification or the primary source cited, and important caveats about the use of these values noted.

3) Please provide a clear statement about how the model was fitted to the data including goodness-of-fit measure, the numerical algorithm used, which parameter varied, constraints imposed on parameter values, and starting conditions.

4) For uncertainty analyses, please state the sources of uncertainties quantified and not quantified [can include parameter, data, and model structure].

5) Please provide sensitivity analyses to identify which parameter values are most important in the model. Uncertainty estimates seek to derive a range of credible results on the basis of an exploration of the range of reasonable parameter values. The choice of method should be presented and justified.

6) Please discuss the scientific rationale for this choice of model structure and identify points where this choice could influence conclusions drawn. Please also describe the strength of the scientific basis underlying the key model assumptions.

7) If you use the points above (2-6), indicate that these are derived from Geoffrey P Garnett, Simon Cousens, Timothy B Hallett, Richard Steketee, Neff Walker. Mathematical models in the evaluation of health programmes. (2011) Lancet DOI:10.1016/S0140-6736(10)61505-X.

*In response to points 2-7, we include all of this information on the validation of the model in the SimPaths working paper available online at* [*https://www.microsimulation.ac.uk/publications/publication-557738/*](https://www.microsimulation.ac.uk/publications/publication-557738/)*. We cite this paper at appropriate points throughout the manuscript and appendix. We do not feel that it would be valuable to repeat this information in the Appendix, but instead focus on the modifications and additional validation exercises required for this particular analysis, which are described in detail. We hope that this sufficiently addresses these points, but would be happy to further discuss if needed.*

8) Under the subheading ‚Sensitivity analysis‘ you write “…two structural sensitivity analyses for Scenarios 3 and 4 (Full UBI).”. Shouldn’t Scenario 4 be ‘Full+ UBI’? Please revise throughout the entire main manuscript. Also, please be consistent/clear in your description of the different scenarios, e.g., Scenario 3, Scenario 3 (Full UBI) or Full UBI.

*We appreciate this was unclear, and have revised accordingly. The scenarios are only referred to by number on initial introduction and in Table 1, after this we refer to them by name throughout the manuscript.*

9) Please define ‘bn’ at first use.

*We have now done this.*

10) Please define ‘RII’ and ‘SII’ at first use.

*We have now added this acronym at first use earlier in the Methods section:*

*“Relative and slope indices of inequality (RII/SII) in CMD (by education) were calculated for the whole working-age population, and for men and women separately.”*

11) “Under these conditions, in a reversal of the primary analysis findings, there was a small short-term reduction in prevalence of CMD in Full UBI scenarios: -0.27% (-0.49, -0.05) in 2023, equivalent to a reduction of 112,228 CMD cases (20,783-203,673) (Table 2).” – In this sentence and the following paragraphs, you switch between "Full UBI" and "Full+ UBI", while Table 2 only shows results for the Full+ UBI scenario, please revise.

*We apologise that this was unclear, we have revised the wording accordingly throughout this section of the manuscript to make our intention clearer.*

DISCUSSION

Please discuss what the study adds to existing research and where and why the results may differ from previous research. Please remove the ‘Conclusion’ heading as the one-paragraph conclusion should be part of the discussion.

*We have amended the Discussion as suggested by yourself and also the Reviewers, and have removed the Conclusions heading.*

TABLES

1) Please define abbreviations used in each table (including those in Supporting Information files).

*We have now done this.*

2) Table 1: Please define ‘pensioners’ (i.e. starting age). Please add definitions for the four different scenarios.

*We have now done this.*

3) Table 2: Please define ‘OECD’, ‘UK’, ‘N.’. Please state the meaning of the numbers in brackets/define all numerical values for the reader (e.g., “ABSOLUTE % DIFFERENCE IN CMD PREVALENCE (95% Uncertainty intervals)”).

*We have added the requested definitions, and added a statement to the table caption indicating that the 95% Uncertainty Intervals are in brackets.*

FIGURES

1) For all Figures, please ensure that you have complied with our figures requirements <https://journals.plos.org/plosmedicine/s/figures>.

*We have reviewed the figure requirements and believe that we adhere to these in our revision.*

2) Please consider avoiding the use of red and green in order to make your figure more accessible to those with colour blindness.

*We have amended the shading of the line colours in all graphs, using the Adobe Color Accessibility Tool to ensure the new choices were colourblind safe. We have also introduced different point shapes for each of the four policy scenarios to add redundancy from an accessibility perspective, and to ensure the scenarios can be differentiated even in black and white. Lastly, we have had the revised graphs reviewed by a member of our research team who is colourblind, to ensure that they were satisfied with the changes.*

3) Please in the figure legend/description, define abbreviations used in each figure (including those in Supporting Information files).

*We have now added this to all figures which include abbreviations, including in the Appendix.*

4) Please provide titles, legends and descriptions for all figures (including those in Supporting Information files).

*As above, we have now added figure descriptions where these were previously missing and are required to explain abbreviations or scenarios.*

5) Figure 2/Figure 3: Please add definitions for the four different scenarios. Please indicate in the figure caption the meaning of the whiskers. Please show the axis beginning at zero. If this is not possible, please show a break in the axis. Please add x-axis labels. The red line which denotes the reform implementation point should be described as dashed red line (also see the comment #2). For Figure 2, please introduce the abbreviation ‘UBI’ in the figure title (as done e.g., in Figure 3).

*We have added definitions of the policy scenarios and the meaning of the whiskers to the Figure captions. We have also amended the description of the dashed red line as requested, and have introduced the abbreviation UBI in the title. After trialling x-axis labels to indicate that the values 2022 etc. were years, we have not included this change in our revision – in practice it made the Figures less easy to read, since it added additional crowding under the x-axis where the legends are. However, we note that the title of the Figures makes clear that these values are a period of time from 2022 to 2025, which is hopefully sufficient. If not, we would be happy to reassess this.*

*We attempted a version of the graphs with the axis beginning at zero, but this severely impacted on legibility and interpretability. We therefore intended to add an axis break, but have unfortunately been able to do this as this is not possible in the ggplot package that we used to create the graphs.*

6) Figure 3: The color scheme for the different scenarios does not match the one used in Figure 2. Please use a consistent color scheme throughout the manuscript (including the figures in the supplementary materials).

*Please excuse this oversight, which was due to a previous change in the ordering of the colours for each policy scenario. This has now been corrected in the revision.*

7) Figure 4: Please add definitions for the two different scenarios. Please show the axis beginning at zero. If this is not possible, please show a break in the axis. Please add x-axis labels. The red line which denotes the reform implementation point should be described as dashed red line (also see the comment #2). Please define ‘y’ or write ‘years’ in full. Please mention that the shaded areas show the 95% uncertainty intervals (e.g., “…95% uncertainty intervals (shaded areas)…”).

*Please see our response to point 5) above for the general comments which apply to both sets of graphs. In addition, for Figure 4 we have also now defined years in the footnote, and have clarified the meaning of the shaded areas (ribbons).*

SUPPLEMENTARY MATERIAL

1) Please define abbreviations used in the supplementary text (at first use), in the supplementary figures and tables.

*All abbreviations in the supplementary figures and tables have now been defined within the captions or titles.*

2) For references in the supplementary material, please see REFERENCES.

*We have now amended the references in the Appendix to be in keeping with the guidance.*

3) For supplementary figures and tables, please see the general comments under TABLES and FIGURES (color, abbreviations, titles, descriptions, axis labels, units etc.) and amend accordingly.

*We have made the same changes as noted above.*

4) Figure S7-S16: Please see comments for Figure 2/3/4 and amend accordingly.

*We have made the same changes as noted above.*

5) For supplementary tables: Please ensure to state the unit of the numbers presented and the meaning of the numbers in brackets/define all numerical values (e.g., for Table S8 and S11 change to “Estimated prevalence of common mental disorders (%, 95% Uncertainty intervals)…”).

*We have now added this to the supplementary tables where it was missing.*

REFERENCES

1) PLOS uses the numbered citation (citation-sequence) method and first six authors, et al.

*We have amended our referencing in the manuscript to the PLOS format.*

2) Please ensure that journal name abbreviations match those found in the National Center for Biotechnology Information (NCBI) databases (<https://www.ncbi.nlm.nih.gov/nlmcatalog/journals>), and are appropriately formatted and capitalised.

*We have amended our reference style to fit the PLOS format and ensured appropriate capitalisation.*

3) Where website addresses are cited, please specify the date of access.

*We have now noted when any web pages were cited/accessed in the reference list.*

4) Please also see <https://journals.plos.org/plosmedicine/s/submission-guidelines#loc-references> for further details on reference formatting.

**GENERAL COMMENTS**

Please respond to all editor and reviewer comments.

1) Please cite the reference numbers in square brackets (e.g., “We used the techniques developed by our colleagues [19] to analyze the data”). Citations should be preceding punctuation.

*We have now done this.*

2) Please cite your Supporting Information as outlined here: <https://journals.plos.org/plosmedicine/s/supporting-information>

*We believe this is already done, but would be happy to make further changes if required.*

3) Please include page numbers and line numbers in the manuscript file. Use continuous line numbers (do not restart the numbering on each page).

*We have added this.*

4) Please report your study according to the relevant guideline, which can be found here: <https://www.equator-network.org/> . We suggest including the completed CHEERS (Consolidated Health Economic Evaluation Reporting Standards (CHEERS) 2022 statement) checklist as Supporting Information. When completing the checklist, please use section and paragraph numbers, rather than page numbers. If you do not feel that the checklist is appropriate, please feel free to include an alternative, or if there is not an appropriate option, please leave it out.

*As the CHEERS checklist is designed for health economic evaluations, not for prospective simulation modelling, it is not particularly well suited to our paper, and we have also been unable to identify any more appropriate checklist available for this purpose. However, in the development of our model and reporting of our work we referred to the paper series from the ISPOR-SMDM Modelling Good Research Practices Task Force and, where applicable, have integrated their recommendations into our process throughout:*

*Caro JJ, Briggs AH, Siebert U, Kuntz KM. Modeling Good Research Practices—Overview: A Report of the ISPOR-SMDM Modeling Good Research Practices Task Force–1. Medical Decision Making. 2012;32(5):667-677. doi:10.1177/0272989X12454577*

5) Please include the Ethics statement in the according section of the online submission form.

*We will do this, thank you.*

6) The terms gender and sex are not interchangeable (as discussed in <https://www.who.int/health-topics/gender#tab=tab_1> ); please use the appropriate term (including in the supplementary materials) and revise throughout the entire manuscript.

*We use the term gender throughout the manuscript, as the data we draw from include self-reported measures of (binary) gender which we feel is more likely to represent gender identity, as opposed to sex.*
