# Supplementary material for: Short-term impacts of Universal Basic Income on population mental health inequalities in the UK: A microsimulation modelling study
Source: PLoS Med. 2024 Mar 4;21(3):e1004358. doi: 10.1371/journal.pmed.1004358 (PMC10947674; doi:10.1371/journal.pmed.1004358)
Supplement: Attachment — Submitted filename: PM UBI microsim revision - second response letter v1.docx [file pmed.1004358.s003.docx]

*Dear Editors,*

*We would once again like to extend our sincere thanks to you and the reviewers for the time spent rereviewing our paper and for the decision to accept it pending our response to the remaining editorial comments. We provide a point-by-point response to the comments below, with our responses in italics throughout.*

**COMMENTS FROM THE REVIEWERS:**

Reviewer #1: The authors have responded in full to my points, and to the points raised by the other reviewers. The responses are detailed and thoughtful and I believe the paper will now make a significant and useful contribution to the literature.

*We thank the reviewer for their positive comments on the paper’s contribution, and are glad to have addressed their comments.*

Reviewer #2: Response to major comments: The authors have provided a detailed and comprehensive response to the major suggested revisions, specifically, the concerns raised by several reviewers on the macroeconomic impacts of the UBI on the outcomes of interest. The paper is now much clearer on the limitations posed by the key assumptions, and the justification of the considerable additional complexity of integrating a macroeconomic model at this stage are reasonable.

*We are glad that our revisions to the paper were sufficient to overcome the concerns about the macroeconomic impacts of UBI not being able to be included within the modelling at this stage, and appreciate the Reviewer’s positive response to our revisions.*

Response to minor comments:

R2.7: I appreciate the authors' response that stratification of results based on tax brackets is not possible due to the separation between the UBI modelling within UKMOD to the dynamic health modelling within SimPaths. While the UKMOD output includes a calculation of the Gini coefficient for each policy scenario, it may be interesting to include a comparison between current income distribution (by tax brackets) and income distribution under each of the scenarios as an appendix (in addition to Figure 2), with a very brief note in the discussion that references the current distribution of CMDs across the income distribution, and a note that the minimal change under the UBI scenarios may be due to changes in the proportions of people in each tax bracket.

*We appreciate this additional point, however, unfortunately we are not able to easily extract this information from our data to include it within the Appendix.*

Reviewer #3: Thank you to authors for their detailed response to each of my comments. I am now happy to approve this paper for publication.

*We thank the reviewer for their time and response to our revision.*

**REQUESTS FROM EDITORS:**

ABSTRACT

1) Please note that you can remove the units from the 95% UI values in parentheses. Since the corresponding unit is presented with the foregoing value, it is not necessary to repeat the units. Also, please ensure to add 95% UI before each set of parentheses and that the 95% UI values are presented in parentheses (please see comment 3) for an example).

*We have made these changes.*

2) ll.28-29: Please add "to our knowledge" or something similar.

*We have added this caveat.*

3) ll.45-46: It seems the second pair of parentheses should directly follow ’74.1%’. Please revise. Editorial suggestion: “(for Full+ from 78.9% (95% UI [77.9,79.9]) to 74.1% (95% UI [72.6, 75.4]))”

*We have amended the sentence as requested.*

4) In the last sentence of the Abstract Methods and Findings section, please describe the main limitation(s) of the study's methodology.

*We have added the following sentence to the end of the Methods and Findings section:*

*“The main limitations of our methods are the model’s short time horizon and focus on pathways from UBI to mental health solely via income, poverty, and employment, as well as the inability to integrate macroeconomic consequences of UBI; future iterations of the model will address these limitations.”*

AUTHOR SUMMARY

We suggest changing the last bullet under ‘What Do These Findings Mean?’ point to: The main limitation of our modelling study is that it looks at how UBI would influence mental health only through income and employment, while other pathways were not considered in the analysis.

*We have amended the bullet point to:*

*“The main limitation of our modelling study is that it looks at how UBI would influence mental health only through income and employment, and other pathways might also be important to include in future research.”*

INTRODUCTION

ll. 110-111/115-116: Please add “to our knowledge” or similar.

*We have added this caveat in lines 115-116 (now 120). In lines 110-111 (now 115-116) we are stating the conclusions of the systematic reviews cited in the preceding sentence, as none of the included studies within these did evaluate an actual UBI (which the reviews stated in their findings), so we did not feel that this caveat was necessary to add here – we have instead amended the statement slightly to make this clearer.*

METHODS AND RESULTS

1) l.248. Please define ‘OECD’ at first use.

*We have now added the full definition here.*

2) l.329 and ongoing: Please see comment 1) under ABSTRACT and adjust the statistical reporting accordingly (for example, line 329: 0.01% (95% UI [0.00, 0.03])).

*We have now made these changes to the results presented in the paper.*

3) ll.331-333: Please provide reference to the relevant graph and/or table.

*We have now noted that these results are found in Table S6 in the Appendix.*

4) ll.336-337: Please check whether Table S6, Appendix is the (only) appropriate reference here. It seems the relevant data of employment rates described here are also visible in Figure 2 (right panel).

*This information is indeed presented in Figure 2 as well as Table S6. However, we felt as this was a continuation of the results introduced in the previous sentence, where Figure 2 was referenced, it did not necessarily need to be repeated here? However, we would be happy for this to be added if the Editors prefer.*

5) ll.337-338: Please provide reference to the relevant graph and/or table.

*We have added another reference here to Table S6, Appendix.*

6) ll.338-340: You state that the employment effects of the Full UBI policies were sustained throughout the study period after implementation. However, when looking at Figure 2, one can see that while employment rates initially fell in both Full UBI scenarios after policy implementation, they rose steadily in the following years. You might consider modifying this conclusion accordingly.

*We have modified the wording of this as follows, which we hope makes our original point clearer:*

*“As with poverty, the differential trajectories of lower employment for the Full UBI policies compared with Baseline were sustained throughout the study period following implementation.”*

7) ll.443-450: We suggest briefly reiterating what the GHQ Likert scores reflect, as it may be easier for readers who are not as familiar with the content to follow this section.

*We agree, we have added the following information in brackets at this point to help clarify this for readers:*

*“Results for our secondary outcome (GHQ Likert score, which represents a continuous measure of psychological distress rather than a binary measure of likelihood of CMD) are shown in Tables S14-S15, Appendix”*

FIGURES

1) Please provide a figure description for Figure 1.

*We have added a figure description indicating the contents of the two panels in Figure 1.*

2) Figure 4: In the figure description, we suggest adding a definition of low, medium and high education.

*We have now added this to the Figure 4 caption.*

REFERENCES

1) Please thoroughly revise all references and ensure that journal name abbreviations match those found in the National Center for Biotechnology Information (NCBI) databases (<https://www.ncbi.nlm.nih.gov/nlmcatalog/journals>), and are appropriately formatted and capitalised (e.g., for reference [1] The Lancet Regional Health – Europe should be Lancet Reg Health Eur)

*We believe these have now all been corrected.*

2) When specifying the date of access, please write “accessed” instead of “cited”.

*We have revised the template accordingly.*

3) Please revise the format of reference [65].

*The formatting of the author list for this reference has now been corrected.*

SUPPLEMENTARY MATERIAL

1) p.7, please change to: “…considerable debate on the ‘best’ way to fund UBI.”

*We have made this correction.*

2) Figure S1/S13/S14 (and where applicable): Similar to Figure 4, we suggest adding a definition of low, medium and high education.

*We have now added this information to the relevant figure and table captions.*

3) Table S4: Please change "Full years UBI only" to "Full UBI years only".

*We have made this correction.*

4) Table S8/S11/S15: We suggest adding a definition of low, medium and high education and changing “Younger 25-44y” and “Older 45-64y” to “Age group: 25-44 years” and “Age group: 45-64 years”.

*We have made these requested changes.*

5) Table S6/S9: We wondered why you chose to present only the differences and not the actual values for average weekly hours worked, and why you chose not to present the differences for median annual income, poverty, and employment.

*The reason for presenting the difference in weekly hours is that this is of most interest rather than the absolute values of average hours worked – the inclusion of these figures is to see how many fewer hours individuals work under the UBI scenarios even if they did ‘choose’ to stay working in the model. In contrast, the overall/absolute levels of annual income, poverty, and employment were the more relevant and interesting values for these indicators. To include the change measures for all of these in addition to the absolute values was, we felt, unnecessary and risked adding too much detail to an already very full Appendix.*

6) Figure S12: Please note that in Figure S12 you use the term " economic inactivity causal effects" while in others you use the term " economic inactivity effects" - please try to be consistent.

*We have now amended the references to this so that all read ‘economic inactivity effects’ in both the appendix and the main paper.*

7) Please revise the references according to the comments under REFERENCES.

*We have now done this.*

SOCIAL MEDIA

To help us extend the reach of your research, please provide any X (formerly known as Twitter) handle(s) that would be appropriate to tag, including your own, your coauthors’, your institution, funder, or lab. Please respond to this email with any handles you wish to be included when we tweet this paper.

@Rachel_Thomson

@vkatikireddi

@igelstorm

@AlastairLeyland

@andybaxter

@theSPHSU

------------------------------------------------------------

General Editorial Requests

1) We ask every co-author listed on the manuscript to fill in a contributing author statement. If any of the co-authors have not filled in the statement, we will remind them to do so when the paper is revised. If all statements are not completed in a timely fashion this could hold up the re-review process. Should there be a problem getting one of your co-authors to fill in a statement we will be in contact. YOU MUST NOT ADD OR REMOVE AUTHORS UNLESS YOU HAVE ALERTED THE EDITOR HANDLING THE MANUSCRIPT TO THE CHANGE AND THEY SPECIFICALLY HAVE AGREED TO IT.

*We have included the contributing author statement and also uploaded individual ICJME forms for all included authors.*

2) Please ensure that the paper adheres to the PLOS Data Availability Policy (see https://eur03.safelinks.protection.outlook.com/?url=http%3A%2F%2Fjournals.plos.org%2Fplosmedicine%2Fs%2Fdata-availability&data=05%7C02%7CRachel.Thomson%40glasgow.ac.uk%7Cf0bc2e7b54fc4f77e75208dc18d61922%7C6e725c29763a4f5081f22e254f0133c8%7C1%7C0%7C638412555021769617%7CUnknown%7CTWFpbGZsb3d8eyJWIjoiMC4wLjAwMDAiLCJQIjoiV2luMzIiLCJBTiI6Ik1haWwiLCJXVCI6Mn0%3D%7C3000%7C%7C%7C&sdata=tkwAi8vIgUvvNjZdkiuLetneOfjPPUMZK%2BmfW1DAPNg%3D&reserved=0), which requires that all data underlying the study's findings be provided in a repository or as Supporting Information. For data residing with a third party, authors are required to provide instructions with contact information for obtaining the data. PLOS journals do not allow statements supported by "data not shown" or "unpublished results." For such statements, authors must provide supporting data or cite public sources that include it.

*We believe that we have adhered to this policy.*

3) Please review your reference list to ensure that it is complete and correct. If you have cited papers that have been retracted, please include the rationale for doing so in the manuscript text, or remove these references and replace them with relevant current references. Any changes to the reference list should be mentioned in the rebuttal letter that accompanies your revised manuscript*.*

*We have reviewed the reference list and believe it is complete and correct.*
